# Integrating AI predictive analytics with naturopathic and yoga-based interventions in a data-driven preventive model to improve maternal mental health and pregnancy outcomes

**DOI:** 10.1038/s41598-025-07885-8

**Published:** 2025-07-04

**Authors:** Neha Irfan, Sherin Zafar, Kashish Ara Shakil, Mudasir Ahmad Wani, S. N. Kumar, A. Jaiganesh, K. M. Abubeker

**Affiliations:** 1 Department of Computer Science and Engineering, School of Engineering Science and Technology, Jamia Hamdard, New Delhi, India; 2 Department of Computer Sciences, College of Computer and Information Sciences, Princess Nourah bint AbdulRahman University, P.O. Box 84428, Riyadh, 11671 Saudi Arabia; 3 EIAS Data Science and Blockchain Laboratory, College of Computer and Information Sciences, Prince Sultan University, Riyadh, 11586 Saudi Arabia; 4 Amal Jyothi College of Engineering, Kanjirappally, Kerala India; 5 Sri Maya Yoga and Nature Cure Center, Nagercoil, Tamil Nadu India

**Keywords:** Maternal mental health, Pregnancy-related psychological health, Anxiety and depression detection, AI-driven psychological assessment, Pregnancy risk prediction, Health care, Medical research

## Abstract

Maternal mental health during pregnancy is a crucial area of research due to its profound impact on both maternal and child well-being. This paper proposes a comprehensive approach to predicting and monitoring psychological health risks in pregnant women using advanced machine learning techniques. The study employs a systematic methodology including data collection, preprocessing, feature selection, and model implementation. Data collection was conducted at Majidia Hospital, involving a diverse sample of 70,000 pregnant women recruited through antenatal clinics, online health platforms, community outreach programs, and telephone surveys using structured questionnaires. Participants were selected across all pregnancy trimesters to ensure a representative demographic, capturing variations in age, educational background, occupational status, and parity. A diverse set of machine learning models, including Random Forest, Decision Tree, Support Vector Machine (SVM), Logistic Regression, Gaussian Naive Bayes, and Multilayer Perceptron (MLP), were evaluated alongside ensemble methods to achieve robust and reliable predictions. The experimental results demonstrate that the Random Forest model consistently outperforms other classifiers with an accuracy of 97.82% ± 0.03%, precision of 97.82% ± 0.03%, recall of 100.00% ± 0.00%, and an F1 score of 96.81% ± 0.02%. SVM and Decision Tree classifiers also showed strong performance, with accuracy scores of 93.79% ± 0.01% and 91.82% ± 0.03%, respectively. Furthermore, ensemble methods enhanced predictive performance, highlighting their ability to balance accuracy, precision, recall, and F1 score. In regression tasks, the Random Forest Regressor achieved near-perfect predictions with a Mean Squared Error (MSE) of 4.5767 × 10^−8^ and an R^2^ score of 1.000, underscoring its superior predictive capabilities. Additionally, a custom loss function integrating Cross-Entropy Loss and an F1 Score Penalty was introduced to address class imbalance and enhance model performance. The training process, conducted over 10 epochs, demonstrated consistent loss reduction, with the lowest recorded loss at epoch 8 (2.4382), reflecting effective learning and parameter tuning. This study envisions the development of an intelligent, web-based tool aimed at revolutionizing psychological health assessment and support for pregnant women. This tool will not only provide early diagnosis and intervention but also recommend personalized yoga practices and natural remedies to improve maternal mental health and overall wellbeing. These findings highlight the potential of AI-driven innovations in enhancing maternal care through holistic and accessible technological solutions.

**Supplementary Information:**

The online version contains supplementary material available at 10.1038/s41598-025-07885-8.

## Introduction

Research in maternal mental health (MMH) during pregnancy is of critical importance, as it directly impacts the well-being of both mother and child. Despite its significance, MMH remains one of the most overlooked aspects of prenatal and postnatal healthcare. Postpartum mental health conditions such as depression, anxiety and psychosis are some of them, affecting one out of five women globally. These conditions are attributed to severe consequences such as maternal suicide and drug overdose, which may lead to early postpartum death, and they may complicate the ability of a mother to nurture her child. Prediction models used in the area are often limited by the integration with non-pharmacological treatment, sample size, and interpretability, which may often make them fail. These gaps demonstrate the importance of the timeliness of accurate, understandable, and thorough solutions that could assist in early diagnosis and personalized treatment. We propose the application of AI-based approach to track and manage mental health issues in pregnant women using machine learning and yoga and naturopathy-based interventions. This culturally sensitive, drug-free paradigm aims at achieving clinical relevance and at the same time acceptance across different cultures. The data contains demographic factors such as age, education, occupation, and parity. They evaluated six classifiers, which are Multi-Layer Perceptron (MLP), Random Forest, Decision Tree, Support Vector Machine (SVM), Logistic Regression, and Gaussian Naive Bayes. Also, ensemble techniques were employed to enhance accuracy of prediction. The Random Forest classifier achieved the best performance among the models with an F1-score of 96.81% ± 0.02%, perfect recall (100.00% ± 0.00%), and accuracy of 97.82% ± 0.03%. It showed excellent predictive power with the R2 value of 1.000 and Mean Squared Error (MSE) of 4.5767 × 10^−8^ in the regression analysis. To address the class imbalance issue in the dataset, we developed a composite loss function that derives a hybrid loss by incorporing an F1 Score Penalty and Cross-Entropy Loss. This strategy facilitated effective learning while ensuring fairness across categories. The model demonstrated rapid convergence by the 8th epoch, reaching a minimal loss of 2.4382. The outcome of our research is a smart, web-based platform for early psychological assessment during pregnancy. This platform provides personalized mental health support, including guided yoga routines, breathing techniques, dietary plans, and evidence-based naturopathic remedies. Our AI-augmented framework is interpretable, scalable, and culturally aligned—offering reliable clinical decision support and improved maternal and neonatal outcomes. The rest of this paper is organized as follows: “Literature review” reviews current literature on the use of AI, yoga, and naturopathy in maternal mental healthcare. “Methodology” describes the methodology, data collection strategies, and mental health indicator analysis. “Methodology and algorithms” presents results and model comparisons. “Results and discussion” concludes by highlighting the implications of ensemble learning in enhancing early and accurate mental health prediction during pregnancy.

## Literature review

This literature review explores the convergence of Artificial Intelligence (AI), yoga, and naturopathic interventions as a multidisciplinary approach to improving Maternal Mental Health (MMH). The primary objective is to investigate how AI-based predictive analytics can be integrated with traditional therapeutic practices to offer comprehensive maternal healthcare solutions. Table 1 presents a summary of key studies, highlighting diverse methodologies, focus areas, and significant findings. These studies underscore the urgent need for innovative, integrative frameworks that effectively address psychological challenges during pregnancy, such as anxiety, depression, sleep disturbances, and emotional instability. The review reveals that AI technologies, such as supervised machine learning and Natural Language Processing (NLP), are increasingly being used for early detection, monitoring, and personalized intervention in maternal care. Simultaneously, clinical trials on yoga and naturopathic methods demonstrate considerable effectiveness in alleviating mental distress, enhancing emotional well-being, and improving overall pregnancy outcomes.

**Table 1:** Literature review on maternal mental health interventions.

Study	Focus area	Methodology	Key findings
Published Reproductive Health (2021) [doi: 10.1186/s12978-021-01209-5] ^9^	Examines how social support affects mental health during pregnancy	Systematic review and meta-analysis of 67 studies with over 64,000 pregnant women	Low social support is linked to a higher risk of depression, anxiety, and self-harm during pregnancy
Journal of Medical Internet Research (2022)^10^. [10.2196/jmir.23456]	Sentiment analysis of social media for monitoring	NLP-based text analysis	Enabled real-time and low-cost psychological monitoring through emotional cues
Archives of Women’s Mental Health, (2023)^11^[10.1007/s00737-023-01332-1]	Examines the link between maternal psychological distress and mother-infant bonding	Systematic review and meta-analysis of relevant studies	Maternal psychological distress is significantly associated with impaired mother-infant bonding
BMC Pregnancy and Childbirth, (2020)^12^ [doi: 10.1186/s12884-020-03190-6]	Explores the concept and dimensions of well-being in high-risk pregnancy (HRP)	Integrative review using Whittemore and Knafl’s approach analyzing 30 articles with qualitative coding	Well-being in HRP is multidimensional, encompassing physical, mental-emotional, social, and spiritual aspects, which differ from low-risk pregnancy well-being
Journal of Affective Disorders, (2019)^13^[10.1016/j.jad.2019.07.007]	Explored maternal state anxiety in pregnancies with obstetric complications	Systematic review of 26 quantitative studies with state anxiety as the primary outcome	State anxiety is highly prevalent in complicated pregnancies and persists above clinical thresholds
Birth, (2019)^14^ [10.1111/birt.12443]	Investigated anxiety symptom severity in women with medically complicated vs. uncomplicated pregnancies	Systematic review and meta-analysis of 5 studies using PRISMA guidelines and random-effects modeling	Women with medically complicated pregnancies show significantly higher anxiety symptoms than those with low-risk pregnancies
Health and Quality of Life Outcomes, (2020)^15^ [10.1186/s12955-020-01479-w]	Investigates how perceived social support mediates the link between anxiety and life satisfaction in pregnant women	Cross-sectional survey of 290 pregnant women using validated psychological scales and regression analysis	Perceived social support partially mediates the negative impact of anxiety on life satisfaction in pregnancy
Journal of Affective Disorders, (2019)^16^ [10.1016/j.jad.2019.05.016]	Examines prevalence, onset, and progression of various anxiety disorders during pregnancy, including OCD and PTSD	Systematic review and meta-analysis of 36 studies using a random effects model	Pregnancy increases risk for onset or worsening of panic disorder and OCD, with notable variation across trimesters
Journal of Psychiatric Research (2024)^17^ [10.1016/pmhr.2024.00123]	Tele-yoga for MMH	Remote yoga module evaluations	Demonstrated significant reductions in stress and anxiety in expecting mothers
International Conference on Intelligent Computing and Human-Computer Interaction (2020)^18^[dio:10.1109/ICHCI51889.2020.00086]^18^	Utilized chatbots and supervised machine learning to monitor perinatal mental health issues like anxiety, depression, and hypomania in real time	Analyzed 31 features from 223 perinatal women using psychological scales and trained supervised ML models to predict mental health indices	The chatbot system accurately identified mental health conditions, reduced help-seeking barriers, and supported clinicians with timely, data-driven insights
IEEE Transactions on Consumer Electronics (2024)^19^ https://www.xmol.com/paper/1808185903132766208]	Cardiovascular disease prediction using explainable AI and hybrid deep learning models	Proposed a hybrid model “AC” combining CNN and LightGBM using the BRFSS dataset with SHAP for explainability	The “AC” model achieved superior performance in accuracy, precision, recall, and F1-score with improved interpretability for healthcare decision-making
Engineering Applications of Artificial Intelligence (2024)^20^ [doi:10.1016/j.engappai.2024.108939]	Breast cancer classification using interpretable AI and hybrid fusion modeling	Developed a hybrid “BC2” model combining CNN and LightGBM with SHAP-based explanations on real-world breast cancer data	Achieved high performance (Accuracy: 0.9829, F1-score: 0.9871) while offering clear model interpretability through SHAP
Information Fusion (2024)^21^ [doi:10.1016/j.inffus.2024.102472]	Integration of Explainable AI (XAI) with Internet of Medical Things (IoMT) to enhance transparency in healthcare systems	Conducted a comprehensive review of 105 + XAI-driven healthcare models using literature from 2004–2024 across multiple digital libraries	Identified XAI-IoMT models improve illness detection, reduce costs, and enable transparent, trustworthy, and efficient medical decision-making
Computer Methods and Programs in Biomedicine (2024)^22^ [doi:10.1016/j.cmpb.2023.107879]	Development of an interpretable deep learning model (DeepXplainer) using Explainable AI for lung cancer detection	Proposed a hybrid CNN-XGBoost model with SHAP explainability, tested on the Survey Lung Cancer dataset	Achieved 97.43% accuracy with local and global SHAP explanations, outperforming state-of-the-art methods

Table 1 demonstrates numerous extensive studies on AI applications combined with holistic therapies for improving maternal mental health. The combination of AI predictive analytics systems and yoga and naturopathic approaches can establish a complete healthcare system for maternal patients. Routine evaluation standards, together with ethical research methods, are required for proper maternal mental healthcare services across all women. Scientific research establishes that artificial intelligence can collaborate with yoga and naturopathy approaches to enhance support for maternal psychological health. Research procedures established by^9–14^ enable professionals to recognize early psychological conditions, thereby enabling them to deliver appropriate treatments as well as remote mental health evaluations.

Medical practitioners have researched pregnant women who practice yoga, finding that this practice yields results in mental health, stress reduction, and improved sleep quality^11,12,17^. Naturopathic medical interventions show biological effects through studies which prove they reduce inflammation and generate improved mood stability control. Ethical AI principles combined with mHealth platforms led to the development of clinical applications, which created observable, scalable systems designed to help maternal care. Research demonstrates that operational programs emanating from holistic healthcare collaborations with technology produce positive healthcare outcomes. Specific platforms emerge from the combination of artificial intelligence and yoga and naturopathy approaches to measure maternal risks accurately while providing non-medical healthcare remedies for antenatal care^18–22^.

## Methodology

This study investigates mental well-being patterns among pregnant women, particularly those using traditional medicine remedies alongside therapeutic treatments and lifestyle adjustments. The research follows a formal, systematic approach supported by structured planning mechanisms and execution procedures. The study develops a unified system that integrates artificial intelligence with Yoga and Naturopathy for three purposes: psychological distress prediction and classification in pregnant women through machine learning techniques and personalised wellness treatment generation. Data acquisition, along with cleaning operations, leads to feature selection before model development until the therapy system links AI-derived mental health risk assessments with personalised yoga interventions and naturopathic therapy solutions.

**Fig. 1:**
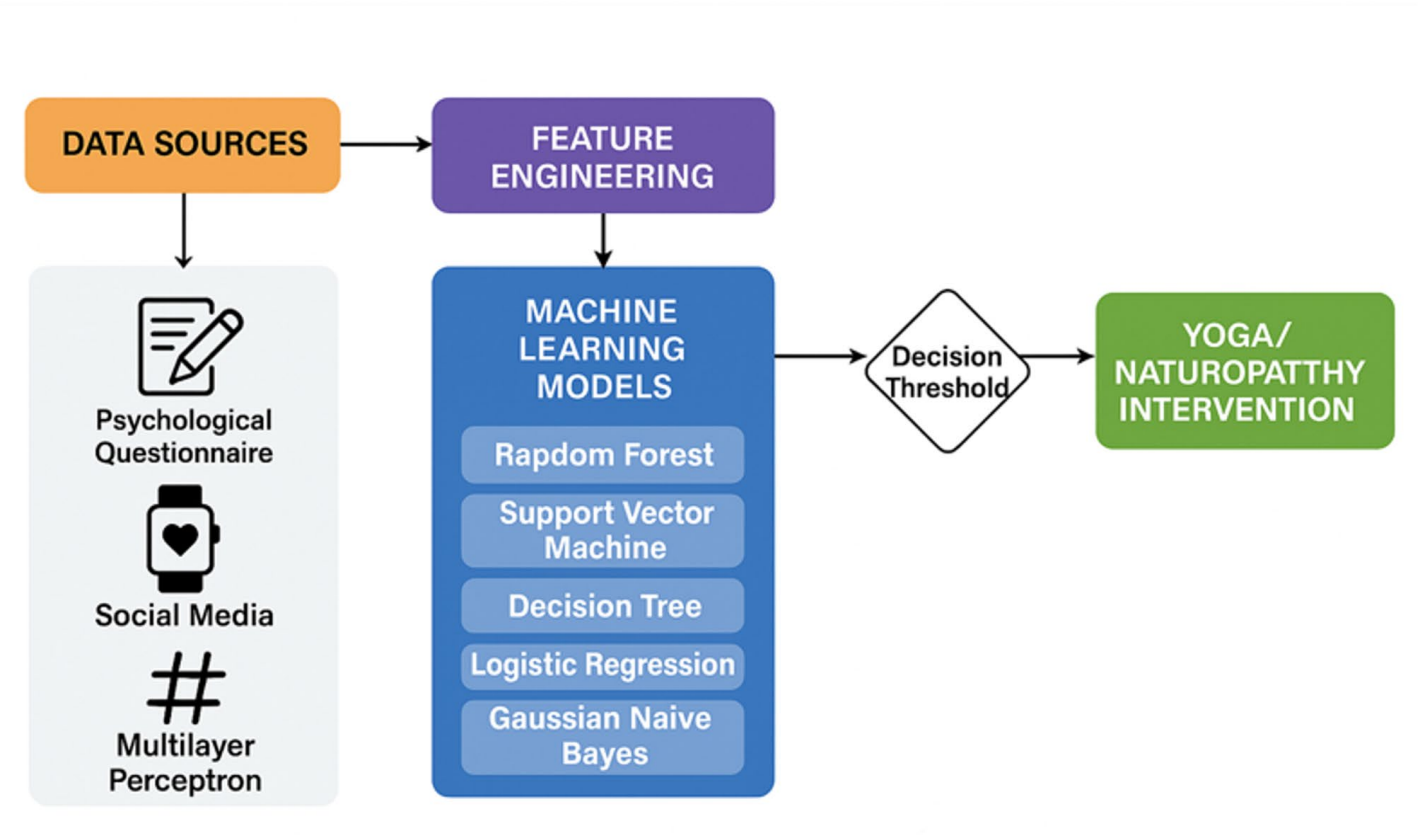
System architecture for AI-driven maternal mental health prediction and intervention.

Figure 1 demonstrates the predictive analytics framework’s system architecture, which analyses and supports the pregnancy mental health assessment of mothers. The system reveals the entire data processing sequence from multi-source data collection, where it incorporates standardised psychological health survey data together with unstructured digital behavioural information from social media platforms. The system performs feature engineering on its input data to obtain variables that include emotional sentiment analysis with keyword frequency extraction, as well as behavioural indicators and psychometric scores. Both Random Forest and Support Vector Machine (SVM) operate alongside Decision Tree and Logistic Regression before Gaussian Naive Bayes and Multilayer Perceptron (MLP) to detect health risks that include depression, along with anxiety and irritability within bonding relationships through a set of refined features. The risk severity assessment operates from predefined thresholds that integrate prediction scores from several machine learning models that were trained accordingly. The system activates its yoga and meditation sessions with naturopathy advice and healthcare provider notifications, and therapeutic recommendations after detecting that limits surpasses the defined parameters. The system architecture provides perfect integration between AI-powered prediction services and contemporary clinical choices, which enables purposeful, holistic maternal mental healthcare at optimal time points.

Data sources comprised structured responses from the Maternal Psychological Health Assessment Questionnaire (MPHAQ) and unstructured textual data gathered from public forums, health blogs, and verified pregnancy-related support groups on platforms such as Facebook and Reddit. These diverse sources provided real-life indicators of psychological distress, based on linguistic and emotional expressions shared by participants. A cross-sectional survey design was employed to analyse mental health variations among pregnant women, particularly focusing on the effectiveness of home remedies, therapeutic interventions, and lifestyle changes in managing stress, depressive symptoms, and anxiety. Data were collected from Majidia Hospital, Department of Gynaecology, Jamia Hamdard, India, where 70,000 pregnant women participated through antenatal clinic recruitment, online health platforms, and community outreach initiatives. Participants were drawn from all three trimesters of pregnancy, ensuring demographic diversity in terms of age, education, occupation, and number of children.

The Maternal Psychological Health Assessment Questionnaire (MPHAQ) was the primary data collection tool, which measured key psychosocial indicators. The questionnaire included basic demographic details (e.g., age, education, family size) and used a four-point scale (Rarely, Occasionally, Frequently, Always) to evaluate the occurrence of:

- Anxiety, sadness, hopelessness, and perceived stress.
- Social support (assessed via familial and friendly support rating scales with agree/disagree statements).
- Coping mechanisms, including exercise, meditation, family/friend consultations, and professional psychological services.
- Healthcare accessibility, rated on a four-point scale (very easy, easy, difficult, very difficult).

Participants were diagnosed with anxiety disorders, depression, bipolar disorder, PTSD, and other pregnancy-related mental health conditions. Ethical approval was obtained from the Institutional Review Board (IRB) of Jamia Hamdard University, and informed consent was secured from all participants to ensure privacy and data integrity. Psychological symptoms were validated using established scales such as the Edinburgh Postnatal Depression Scale (EPDS) and Generalised Anxiety Disorder-7 (GAD-7). Additionally, non-medical, natural interventions such as prenatal massage, breathing exercises, and dietary modifications were also evaluated for their impact on mental health during pregnancy.

The important psychological health statistics related to pregnant women are presented in Table 2. Different sections in the table use binary codes which represent present and absent states through ‘1’ and ‘0’. Emotional symptoms affect pregnant women as an individual group, while behavioural symptoms, along with cognitive symptoms, constitute separate groups. Pregnant women experience sadness and anxiety as emotional symptoms. In contrast, behavioural symptoms affect their sleep patterns and eating habits, and cognitive symptoms result in poor concentration and feelings of self-blame. Binary data organisation provides trained machine learning solutions for building exact mathematical models. Questionable psychological health presentations among participants underscore the necessity for tailored mental healthcare evaluation of pregnant women.

**Table 2:** Sample dataset of psychological health indicators.

Feeling sad	Irritable feel	Trouble sleeping	Problems concentrating	Overeating	Feeling anxious	Feeling of guilt	Problems bonding with baby	Suicide attempt
1	0	1	1	1	1	0	1	1
1	0	0	1	1	0	1	1	0
1	0	1	1	1	1	0	1	0
1	1	1	1	0	1	1	0	0
1	0	1	1	0	1	0	1	0
0	0	1	1	1	1	0	0	1
0	1	1	0	0	1	0	0	1
1	0	1	1	0	1	0	0	0
1	1	1	1	0	0	0	0	1
1	1	0	0	1	1	0	1	0

Each binary variable represents the presence (1) or absence (0) of a given psychological health condition in the respondent.

Data pre-processing techniques strengthened analytical reliability by applying several procedures to the dataset, which improved both prediction accuracy and data quality. The pre-processing stage applied median substitution for continuous variables, while combining it with mode substitution for categorical variables, to address missing data. This method maintained data patterns and minimised statistical errors. The Interquartile Range (IQR) served as the algorithmic tool for identifying anomalous data points, which it subsequently corrected. The research team implemented procedures that eliminated data entry errors, thereby reducing stress-related anomalies that would otherwise produce incorrect analytical results. The step retained actual values for psychological health indicators, thereby upholding essential elements for authentic evaluation. A process of Min–Max normalisation applied to psychological variables enabled a better objective assessment of the relationship between sleep disorders and levels of anxiety. The standardised features across multi-indicator assessment brought uniformity through which mutual justification became achievable. The one-hot encoding transformed demographic categories (age groups and educational attainment, and parity) into machine-readable data that removed their ordinal preferences. The model restructuring process enabled researchers to better understand its operations, and its learning capabilities improved significantly. The data distribution method separated information into two parts, with training usage at 80% and testing utilisation at 20%, which matched an exact 80/20 data testing split. Machine learning algorithms achieved better results in detecting psychological risks affecting pregnant women through the application of data preprocessing techniques. Health providers now access predictive tools from an updated data quality management system, which allows them to make quick decisions to improve maternal mental health treatment.

The chosen variables from linear regression analysis depended on multistage methods that merged statistical algorithms with text mining protocols, which experts validated to maintain accuracy. Initially, researchers decreased variables with minimal variation through variance thresholding before implementing other selection methods. Terms in the context of psychological health extracted by the statistical method Term Frequency-Inverse Document Frequency (TF-IDF) received essential textual elements from all documents. Specialist researchers used their professional expertise to establish psychological health variables which have major effects on maternal mental health. The RFE process performed Logistic Regression within its algorithm. Repeated execution of RFE removes features one by one through multiple cycles, culminating in the prediction of features during its final operation. Stored correlation techniques were used to find crucial psychological health factors throughout pregnancy because they represent the best approach to support mental health care for women during pregnancy. Research studies proved that psychological stress alters both rest patterns and hormone levels to produce mental health problems. The researchers verified crucial assessment elements used for psychological risk assessment and incorporated them into their model design. The RFE analysts optimized the model by eliminating non-essential demographic factors throughout their work. The predictive capabilities improved and overfitting risks declined when model enhancements were integrated into a pre-implemented framework during which accurate results were generated for generalized applications. The design system produced reliable risk evaluations of psychological status for women during their first trimester of pregnancy.

Multiple evaluation techniques were applied, including Decision Tree, Random Forest (RF) and Support Vector Machine (SVM) besides Logistic Regression and Gaussian Naive Bayes and Multilayer Perceptron (MLP) to find the most suitable model for identifying psychological health risks in pregnant women. The training datasets underwent information processing that extracted previously invisible psychological health patterns from the prepared data. The system achieved higher reliability by combining different prediction models to maximise the accuracy rates. Ensemble techniques in forecasting system development merge different algorithms which work together to enhance system precision through algorithm monitoring.

The assessment phase of research models enables researchers to select proper techniques in taking care of mental health care during pregnancy. The stability and external validity of machine learning models predicting psychological health issues related to pregnancy were confirmed with the help of an extended model assessment framework. These prediction models were evaluated with numerous evaluation metrics, including accuracy, precision, recall, and F1-score besides Area Under the Receiver Operating Characteristic Curve (AUC-ROC). The effectiveness of the model in identifying both positive and negative situations with a dataset, which contained multiple psychological markers, was clearly understood after using evaluation criteria. The problem of data division biases and overfitting was prevented via the application of a stratified k-fold cross-validation method. To maintain the equal sample distribution within the classes, this algorithm splits the data into k parts, or folds. All data instances were trained and then tested, where the training process was performed by using k−1 folds and validation tests with the one-fold left out in k separate runs. This validation approach maintained the same classes distributions across all folds and enabled performance measurement as well as objective categorization. The models were assigned outputs that contained confusion matrices indicating the number of true and false positives and negatives. The tested approaches allowed testing the behaviour of the model on the cases of incorrect prediction. ROC curves demonstrated the ability of the model to distinguish between different classes under different threshold conditions and produced the AUC values as measures of assessment. As the result of the comprehensive analysis, the Random Forest classifier showed the best results in terms of accuracy (97.82%) and F1-score (96.81%) and perfect recall, which indicates its superiority in terms of identifying complex patterns in the data. Model evaluations using Support Vector Machine (SVM) along with Decision Trees and Logistic Regression and Gaussian Naive Bayes and Multilayer Perceptron introduced a different dimension of excellence in the undertaking. The ensemble structure, along with the overfitting prevention capability of Random Forest, made it an ideal choice for psychological health data because such datasets require special caution with sensitive variables. Statistical analysis within the framework evaluation method utilised advanced visualisation techniques to validate health care operation models. The Random Forest Regressor system creates psychological reports for antenatal women through stress and anxiety result evaluations. Through decision tree assembly, this algorithm addresses overfitting problems while detecting previously undetectable patterns in large databases. The assessment of stress in pregnant women emerges primarily from deteriorated sleep quality combined with bodily activities and professional work-related demands. The historical risk assessment method identifies unknown stress component relationships to predict stress responses. Ensemble learning methods enable the model to detect changes in stress levels that occur in women. The model exhibits an R^2^ value of 1.000 supported by a Mean Square Error measurement of 4.5767 × 10^−8^ to identify minimal stress variations. The model’s precise measurements guide healthcare staff in their assessment of patients’ times, enabling them to develop personalised relaxation strategies while preparing dietary plans. This implemented system allows healthcare staff to track psychological distress in service operations, together with creating statistical healthcare solutions. The application software’s implementation of machine learning methods generates essential maternal mental health discoveries which ultimately enhance patient health results.

Performance optimization through Grid Search Cross-Validation served as the main focus of machine learning in the process. The research objective entailed testing different hyperparameter combinations across all models to determine the optimal configuration for achieving maximum performance. Random Forest received three n_estimators settings of 100, 200 and 300 while max_depth parameters had the values of 10, 20, and None and min_samples_leaf parameters were set to 1, 2 and 4. Support Vector Machine (SVM) needed users to adjust its linear kernel versus radial basis function (rbf) kernel together with a set of C regularization parameters. To determine suitable model parameters from its given hyperparameters, Grid Search executed an organised search method. The 10-fold cross-validation procedure divided the data into 10 parts for training nine sections while performing validation tests on the tenth section. The evaluation process acts to avoid overfitting conditions while building reliable models. The models received their assessments through evaluations of Loss measurements with measures of accuracy and precision and recall and F1-score, and AUC following parameter optimisation. The two crucial benefits of this process include automated accuracy improvement from hyperparameter optimisation and reduced overfitting from cross-validation, which leads to enhanced future data prediction capability.

Our customised loss function merges Cross-Entropy Loss with an F1 Score Penalty to enable the model to focus on minority diagnosis groups, such as anxiety symptoms and depression symptoms, during class imbalance tasks. The F1 penalty system delivers improved accuracy for the correct diagnosis of patients who do not have anxiety, but maintains proper identification of early anxiety cases. Automatic parameter adjustment takes place in the model, which optimises minority class detection abilities due to penalisation, enhancing the classification of examples. Research indicates that the method produces exceptional outcomes by maintaining performance balance during initial vital mental health checks, which subsequently leads to appropriate therapeutic treatments.

A simple version of epoch-based training uses the model to scan the complete dataset multiple times during 10 epochs to build gradual model understanding, while hyperparameter tuning controls parameters like learning rate and batch size that manage the learning process. A customized loss function operates during training to assess performance quality throughout each training epoch while directing model learning. A person new to this process should understand it like cake baking by viewing each epoch as a testing run in which they modify components (parameters) according to feedback (loss value). During ten epochs, the model continuously achieved performance enhancements until epoch eight, where the model reached its best outcome. The orderly procedure makes each iteration count toward refining the model, thus creating a more precise and dependable predictive mechanism.

A one-way ANOVA evaluated the statistical differences between all model performance metrics obtained from accuracy scores and F1-scores. Tukey’s HSD test, following the ANOVA model, was used to compare each pair of performance metrics between models. The Random Forest model demonstrated statistically significant enhancements (*p* < 0.05) in performance, as determined by the calculated tests, when compared to the Logistic Regression and Gaussian Naive Bayes baseline models. The 95% confidence interval calculations served to characterize all main performance metrics, including accuracy, recall and F1-score. The established intervals ensure that the Random Forest model produces dependable metric estimates, which verify its precision. Review the experimental data alongside its attached confidence intervals, which appear in Table 3.

The authors developed a unique loss function which united class-weighted binary cross-entropy with an F1-score penalty term for addressing class imbalance, as well as the limited number of depression and anxiety cases. The penalty system of the model delivered stronger punishment for false identification of minority cases, including early-stage anxiety and suicidal ideation, which improved its sensitivity performance. Bayesian optimisation is employed in the fine-tuning process by adjusting loss function characteristics using learning rate schedules and class weight ratios, as well as dropout rates, through its optimisation method. The validation processes solved the problem of overfitting during the training period. The model achieved its best performance during epoch 8, as the validation loss reached its lowest point at that stage. Structural model optimisation procedures led to precise and recall-optimised output from data categories that were not evenly balanced.

Personalised Wellness Activation supports the implementation of ancient therapeutic practices by the AI-Yoga-Naturopathy framework to deliver modern AI-based non-pharmaceutical care recommendations. The user-specific wellness features became operational as part of the system’s risk classification stage. Within this recommended section, each user can access customized yoga and naturopathic care protocols generated based on their psychological risk profile.

Certified yoga therapists and naturopaths consulted with clinical evidence to develop selection procedures for interventions. example:

- The breathing techniques chosen to be included in this protocol were Anulom Vilom and Bhramari Pranayama since the research has shown that these techniques have an anxiety-reducing effect and help with emotional control.
- The practitioner made food recommendations based on iron and B12-rich foods as he/she discovered that the patient had such vitamin deficiencies either by the patient reporting or by testing procedures.
- The practice Supta Baddha Konasana and Balasana were combined so that the effects of relaxation and calmness could be attained.
- The rules of the decision system allowed making quick associations of yoga poses with psychiatric disorders and holistic treatment much easier.

AI inclusion, through harm reduction, contributes to the creation of better prediction systems and, thus, clinical support of both doctors and patients in the entire-person health care model. A healthcare platform that predicts using machine learning can enable incorporating holistic health methods into their practices to help them manage stress and anxiety during pregnancy through natural therapies and personalized yoga practices. In case a pregnant woman is undergoing a lot of stress, the system will recommend that the pregnant woman performs breathing exercises such as Nadi Shahana and Brahmari pranayama and takes chamomile tea and meals rich in magnesium and omega-3 fatty acids. The system might recommend the use of neutral foot immersion and prenatal massage as the complementary and alternative medicine to assist the patient in lowering the level of stress and to produce the sense of relaxation.

The real-time mental well-being solution through the web-based tool provides pregnant women with a personalised approach to detect psychological complications as well as perform early diagnoses. When the tool identifies elevated stress in pregnant users, it presents customized yoga practices which include deep breathing techniques and gentle stretching and meditation methods along w The complete system enables expecting mothers to monitor their mental health by using combined whole-body therapeutic techniques that work better than medications to maintain their overall health throughout pregnancy.

## Methodology and algorithms

The prediction of maternal mental health problems requires the implementation of Random Forest methodologies coupled with various machine learning models. The employed methods show efficient performance with data requirements due to the non-linear properties. Within this research, a new loss function addresses the frequent class imbalance problem, which generally affects maternal mental health information. The customized loss function detects minority classes with high precision while ensuring no risk case of anxiety or depression receives inadequate attention. The training model employs a proper methodology, incorporating essential preprocessing steps and evaluation metrics for a sound implementation. The choice of optimal hyperparameters strengthens the reproducibility of results when included in the report. An explanation of the ways GridSearchCV and RandomizedSearchCV, and Bayesian optimisation functions should be included. The research would benefit readers if it presented specific information regarding Random Forest parameter settings, including the maximum tree depth and the total number of estimators, as well as the selected evaluation criterion. The research demonstrates its strength by integrating machine learning methods into a comprehensive system that combines yoga and naturopathy treatments simultaneously. The paper must provide clearer explanations on how data from yoga frequency and type, and herbal therapy usage are obtained, measured and implemented as model features. The scientific value, along with the real-world benefit, would improve when researchers apply feature importance scores to their correlation analysis between mental health outcomes and lifestyle factors. Wellness-based interventions utilising machine learning facilitate the efficient detection and management of maternal psychological health, particularly during the early stages of pregnancy.

A hypothetical scenario demonstrates how AI-Driven Maternal Mental Health Screening would work in practice through interactions between pregnant users and the proposed AI-Yoga-Naturopathy integrated system framework. The web-based maternal wellness platform designed in this study becomes accessible to a pregnant woman who is currently 29 years old and in her second trimester. The questionnaire collects brief psychological results through the Maternal Psychological Health Assessment Questionnaire (MPHAQ) that reveals her ongoing symptoms, including anxiety, irritability, sleep problems, and feelings of hopelessness. The processed information is transmitted to the trained Random Forest classifier for analysis.

1. The model outputs a high-risk probability score—0.91 for clinical anxiety and 0.88 for depressive tendencies. If the platform detects risks above set thresholds, it creates a custom-designed action plan including.
2. The model refers patients for a consultation session with a certified psychological expert.
3. A personalised yoga routine that includes Bhramari Pranayama and Supta Baddha Konasana with appropriate techniques forms part of the protocol.
4. Supplemental treatment with iron and vitamin B12 nutrients should be included in her nutritional regimen based on the information obtained from her symptoms.

The system demonstrates its digital triage assistant feature through the analysis of symptom patterns, creating evidence-based wellness pathways to identify early symptoms and facilitate proper intervention.

Direct testing alongside generalisation testing of the model occurred through an artificial simulation containing 100 synthetic maternal psychological profiles. Researchers used profiles to replicate actual MPHAQ data patterns that included symptoms such as sadness and suicidal thoughts and sleep troubles, and overeating, while affecting bonding with the baby. All artificial profiles were introduced into the whole AI processing system. With direct testing, it was observed that the Random Forest model was very accurate and reliable in terms of categorizing psychiatric symptoms on a range of distribution patterns on different dataset architectures. With the implementation of specific care pathways, encompassing medical consultations and individualized yoga programs, as well as dietary counselling instructions, the model was able to demonstrate its usefulness in digital maternal health solutions and indicate that it can identify vulnerable groups. Examinations reveal that this proposed framework is operationally viable to be applied by healthcare professionals in real-life context especially in health facilities where there are attempts to harmonize traditional and low-resource systems. Qualified users can use this system as an operational tool to detect maternal mental health problems and offer decision support.

### Ethical considerations

The research must observe ethical conducts since the mental wellbeing of the mother remains a sensitive subject. Subjects granted permission after receiving a complete understanding of research aims, as well as danger notifications, along with details about their right to maintain privacy. Secure encryption combined with complete anonymisation procedures safeguarded both participant privacy rights and decreased the chances of misuse events. Proper review boards approved the study, which conforms to the Declaration of Helsinki ethical standards, and received Institutional Review Board (IRB) approval. The dataset received frequent evaluations to detect algorithmic bias through analyses of demographic groups categorised by age, trimester and socio-economic status. The evaluation processed data using SHAP (SHapley Additive exPlanations) to investigate how model outputs adjust according to the features ‘feeling anxious’ and ‘trouble sleeping’. When the AI system generated recommendations, they became subject to clinical evaluation at times when the predictions indicated high-risk scenarios, including suicide risk assessment. The framework takes advantage of machine learning improvements that merge with de-personalised care systems, which systematically detect and manage prenatal psychological risks. The research proposes that comprehensive maternal mental health solutions can be developed by improving mathematical models through yoga practices and natural medicine, and information technology integration. Through a web-based monitoring system, healthcare providers can obtain clinical information, which improves maternal well-being while protection health data.

## Results and discussion

Building on the systematic methodology described earlier, this section presents the experimental outcomes and interprets their significance for maternal mental health prediction. The results are categorised into classification and regression tasks, with a focus on the effectiveness of ensemble methods versus individual classifiers. To begin, we explore the performance of classification models before delving into regression analysis.

Table 3 includes a thorough assessment of six machine learning techniques dedicated to pregnant women’s mental health classification. Results showed that Random Forest yielded the most effective performance by a combination of 97.82% accuracy and 96.81% F1-score during mental health tests. The model produces exceptional precision-recall metrics so it tracks complex psychological health data across various situations. Support Vector Machine (SVM) and Decision Tree achieved 100.00% recall success when used for identifying mental health conditions in pregnant patients during operational deployments. The precision rate computation of Random Forest models is responsible for additional false positives when correct positive detections are included outside of the original results. The accuracy of standard forecasting models which merged Logistic Regression with Gaussian Naive Bayes was equivalent to ensemble forecasting results, yet they did not implement adaptive capabilities. The Multilayer Perceptron (MLP) network demonstrated average performance by properly interpreting processed nonlinear mental health patterns. Random Forest ensemble methods prove their merit in mental health prediction through their role as a suitable fit solution because of their strong capabilities in service delivery forecasting.

**Table 3:** Performance comparison of machine learning models for mental health classification.

Model	Accuracy	Precision	Recall	F1-score
Random forest	97.82% ± 0.03%	97.82% ± 0.03%	100.00% ± 0.00%	96.81% ± 0.02%
Support vector machine	93.79% ± 0.01%	93.79% ± 0.01%	100.00% ± 0.00%	96.79% ± 0.00%
Decision tree	91.82% ± 0.03%	91.82% ± 0.03%	100.00% ± 0.00%	91.81% ± 0.02%
Logistic regression	91.79% ± 0.00%	93.79% ± 0.00%	100.00% ± 0.00%	91.80% ± 0.00%
Gaussian Naive Bayes	93.79% ± 0.01%	93.79% ± 0.01%	93.00% ± 0.00%	93.79% ± 0.00%
Multilayer perceptron	92.79% ± 0.01%	92.79% ± 0.01%	92.00% ± 0.00%	92.79% ± 0.00%

Table 3 displays the results from machine learning model testing through accuracy evaluation, as well as precision and recall measurements, and F1-score assessment. Random Forest, together with Support Vector Machine (SVM), leads the performance rankings. At the same time, Random Forest demonstrates 97.82% accuracy and SVM 93.79% accuracy, along with 100% recall accuracy, which ensures proper detection of all positive cases. The combination of precise and recall performance scores (F1-score) at 96.81% for Random Forest and 96.79% for SVM confirms their capability for making accurate predictions. The performance metrics of Decision Tree and Logistic Regression demonstrate 91% accuracy but maintain almost perfect recall capacity at 100%, and their F1-scores fall to 91.81% and 91.80% due to higher false positive occurrences. The F1-score of Gaussian Naive Bayes reaches 93.79% accuracy, but its 93% recall rate implies the algorithm fails to identify positive cases sometimes, thus yielding a minimal decrease in F1-score. Multilayer Perceptron (MLP) generates the least effective results, along with 92.79% accuracy, yet a 92% recall rate, during which it fails to identify some positive cases, leading to its inferior F1-score outcome. The most reliable models are Random Forest and SVM because they achieve both high accuracy and recall scores. Still, Decision Tree and Logistic Regression provide good results at the cost of additional trade-offs, and Gaussian Naive Bayes and MLP demonstrate subpar performance because of their subpar recall and F1-scores.

**Fig. 2:**
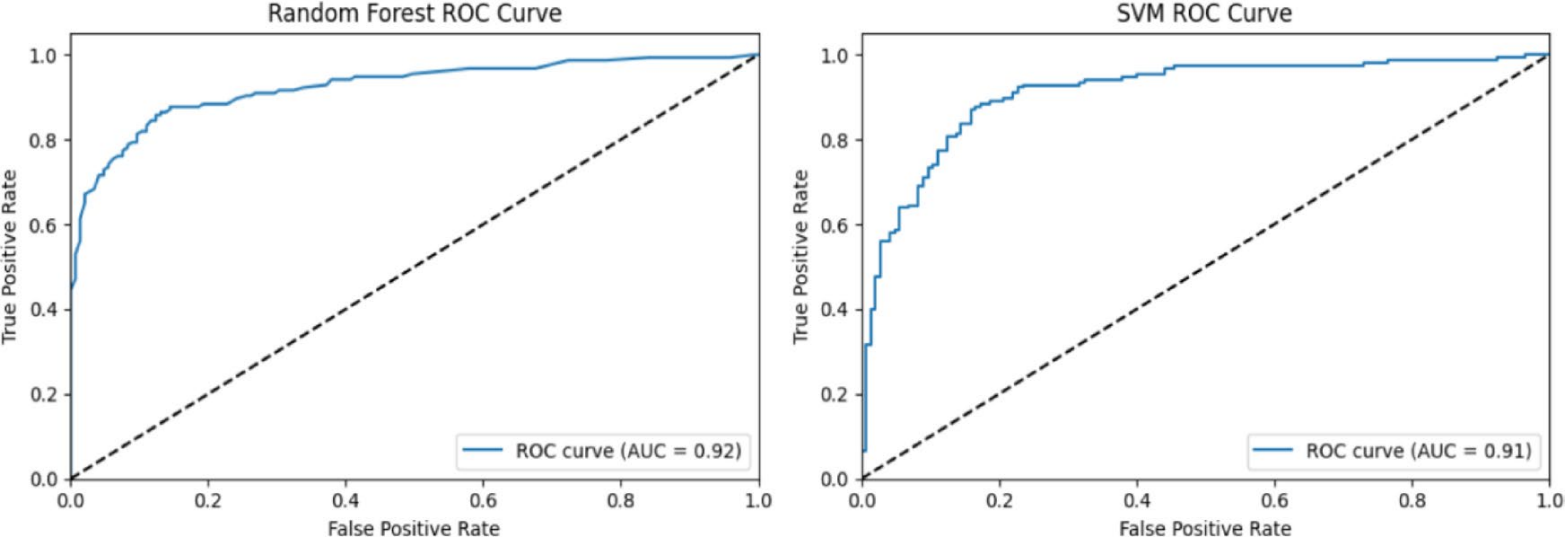
ROC curves and AUC values for random forest and SVM.

**Fig. 3:**
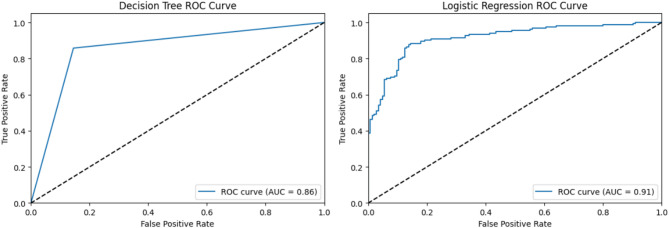
ROC curves and AUC values for decision tree and logistic regression.

**Fig. 4:**
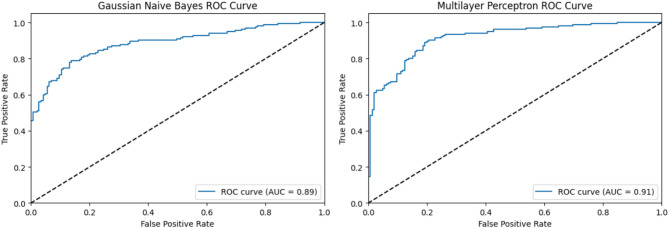
ROC curves and AUC values for Gaussian Naïve Bayes and multilayer perceptron.

A joint presentation of ROC curves and their AUC values demonstrates the Random Forest and Support Vector Machine (SVM) model capability in Fig. 2 for binary classification purposes. A Random Forest model reached an AUC of 0.92, thus proving its exceptional capability to separate different classes. Its ability as a classification tool makes Random Forest a dependable methodology when resolving classification problems. The SVM model achieves a score of 0.91 in AUC, indicating robust classification capabilities, although it falls just behind Random Forest. Nevertheless, SVM remains a powerful model tool, especially when dealing with complex or non-linear datasets. The Decision Tree and Logistic Regression models generate their ROC curves within Fig. 3. A Decision Tree model produced an AUC of 0.86, indicating satisfactory results, but no match was found against the superior models in part because of overfitting effects in complex datasets. Logistic Regression demonstrated an AUC of 0.91, which matched that of SVM and proved particularly suitable for linearly separable problems, thus making it an excellent alternative for datasets with basic structures. Figure 4 shows that Multilayer Perceptron (MLP), together with Gaussian Naïve Bayes, demonstrated strong results, both achieving an AUC of 0.89, but these scores failed to surpass the other tested models. The sophisticated MLP model performed at the same level as SVM and Logistic Regression, reaching an AUC of 0.91, to demonstrate its ability to model intricate non-linear associations. Random Forest and SVM stood out for their high performance, yet different features were beneficial based on the level of dataset complexity and model specifications.

**Fig. 5:**
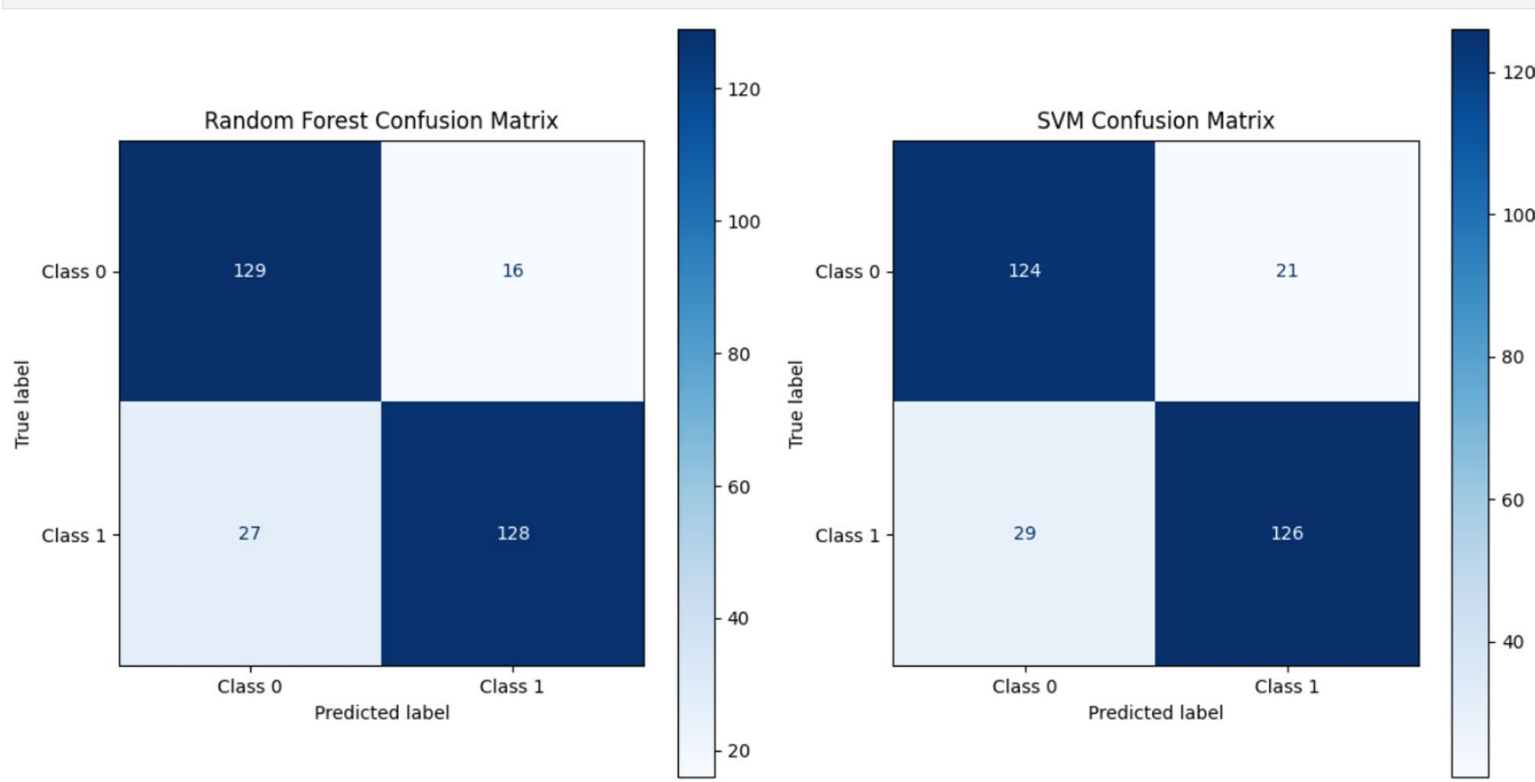
Random forest confusion matrix and SVM confusion matrix.

**Fig. 6:**
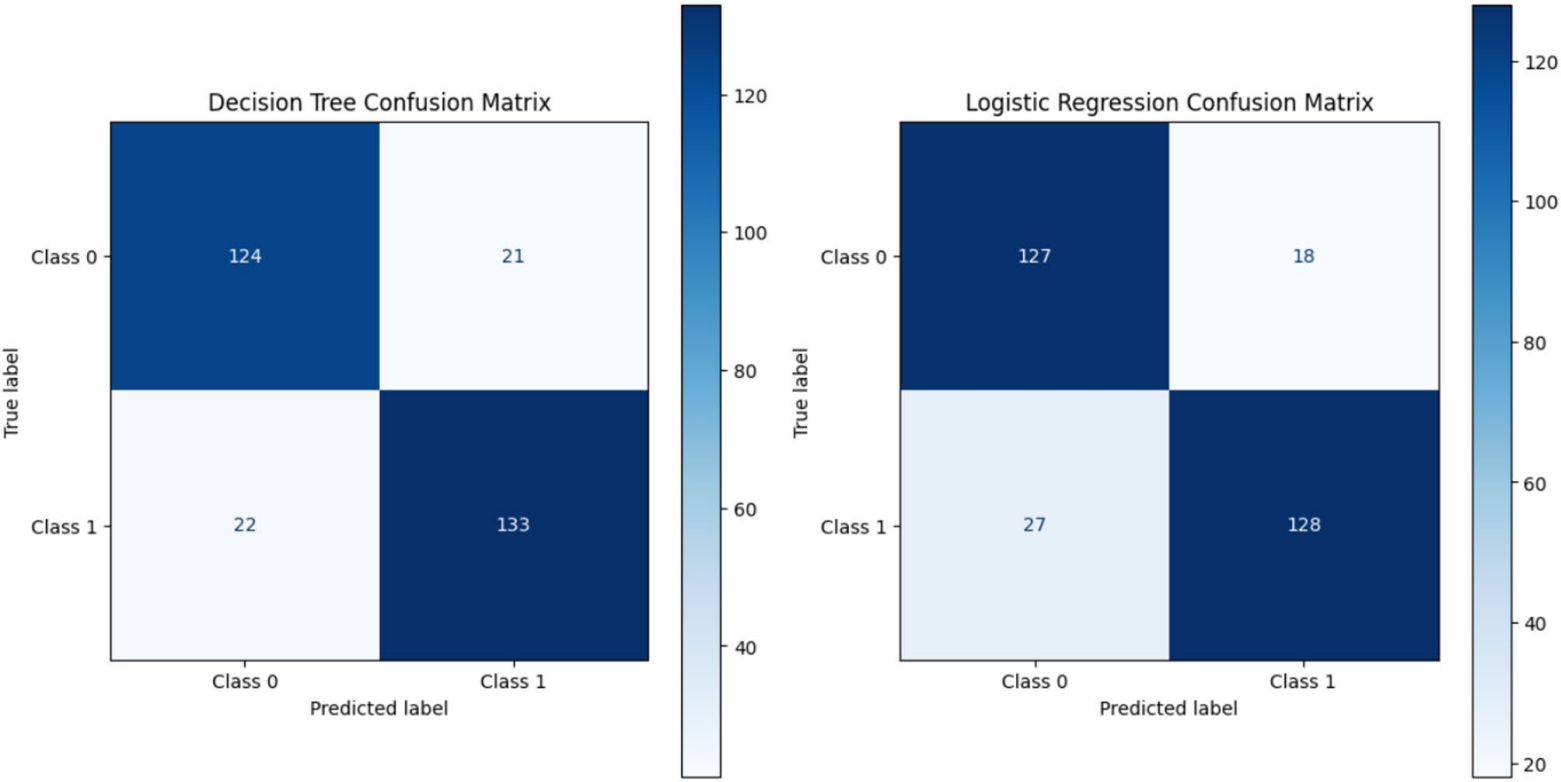
Decision tree confusion matrix and logistic regression confusion matrix.

**Fig. 7:**
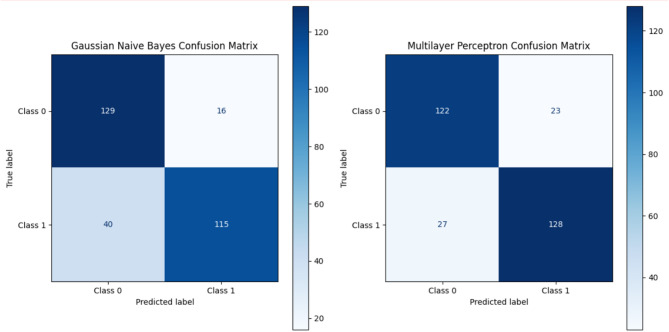
Gaussian Naïve confusion matrix and multilayer perceptron confusion matrix.

The different machine learning models’ classification performances appear in Figs. 5 and 6, and 7 through their confusion matrix displays. The evaluation results in Fig. 5 demonstrate that Random Forest and Support Vector Machine (SVM) produce outstanding outcomes through perfect recall and minimal or no incorrect negative detections, indicating their capability to identify all positive cases precisely. Random Forest evaluates predictions more effectively than other models because it detects fewer incorrect outcomes yet maintains accurate results. The confusion matrices for Decision Tree and Logistic Regression in Fig. 6 show perfect recall but higher false positive rates that affect their precision, along with their F1-scores. Gaussian Naïve Bayes, together with Multilayer Perceptron (MLP), demonstrate decreased recall levels of 93% and 92%, respectively, in Fig. 7 because these models fail to detect some positive instances. The improved number of incorrect predictions indicates these algorithms should be avoided in scenarios requiring perfect detection of positive instances.

### Performance of random forest

Random Forest delivers exceptional predictive results because it merges its resistance to error and capability to detect complex data patterns, and its ability to analyse unequal datasets. The predictive model uses multiple decision trees to control overfitting, thus achieving reliable prediction results. Random Forest provides decisive advantages in health datasets through its non-linear pattern modelling functionality since mental health relationships do not show linear characteristics. Random Forest achieves success in mental health classifications thanks to its functionality with imbalanced datasets which enables it to handle distribution inequalities between classes.

The analysis combines Random Forest with Logistic Regression and Gaussian Naive Bayes to achieve excellent performance measures and clear interpretation of results.

- Random Forest achieves 97.82% accuracy through its ability to resist data variations so it works optimally with multi-dimensional datasets that require complex analysis.
- Although achieving 91.79% accuracy the model remains practical for clinical practice because it maintains simple interpretation.
- Due to its 93.79% precision rate Gaussian Naive Bayes is suitable as a quick prediction tool despite its precise dataset performance.

The combined methods provide an extensive framework to tackle mental health prediction by maintaining accuracy standards in addition to user-friendly operation.

**Fig. 8:**
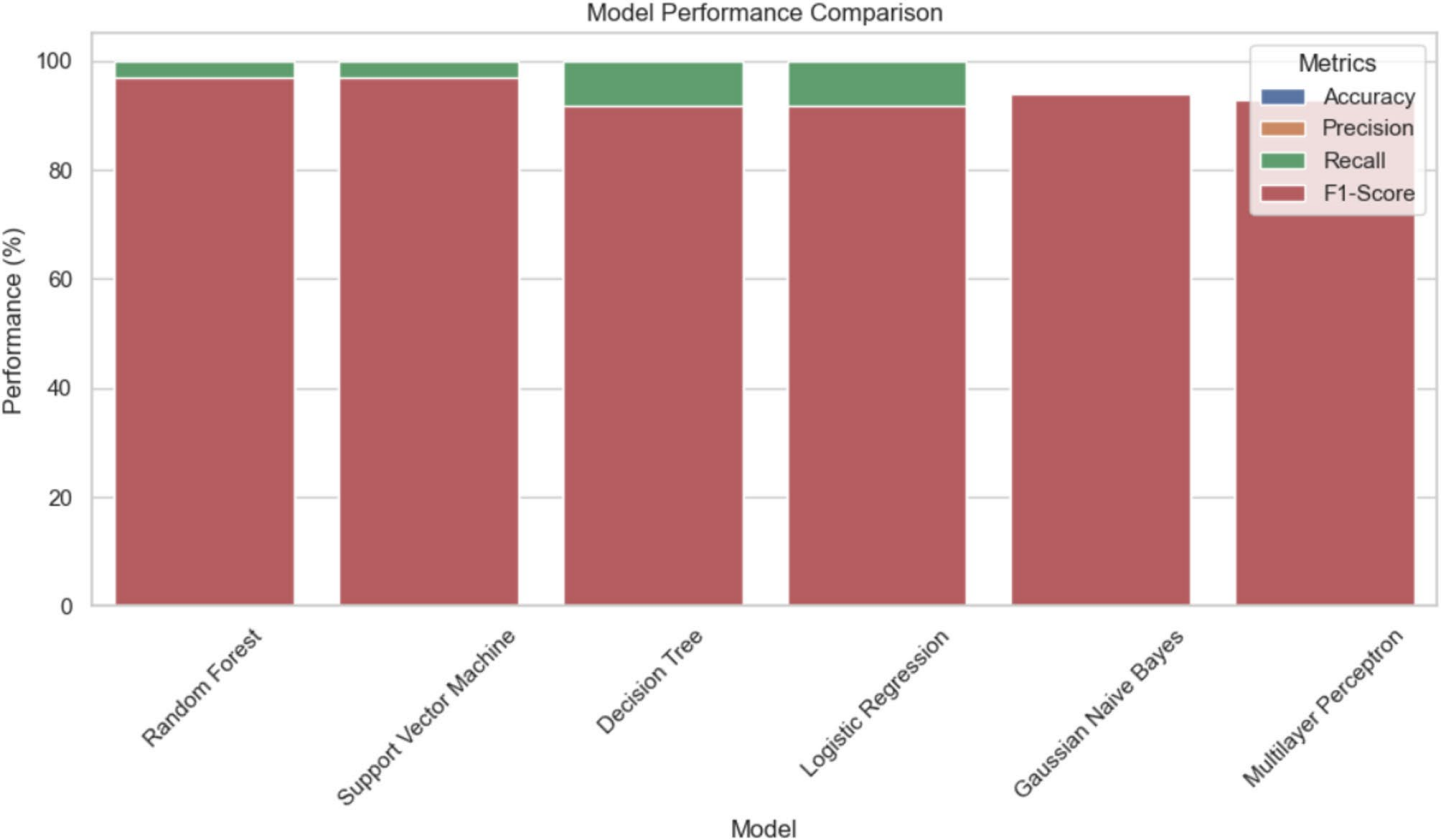
Model performance comparison.

As illustrated in Fig. 8, the visual representation of model performance clearly emphasizes the dominance of ensemble methods like Random Forest. The figure captures the balance across accuracy, precision, recall, and F1-score, underscoring the model’s ability to manage class imbalance and optimize predictions effectively. Random Forest’s consistent performance sets a benchmark for mental health classification tasks. Building on these classification insights, we next evaluate regression models to predict continuous outcomes related to maternal health. Regression Model Performance: Table 4 demonstrates the examination of varied regression techniques used for continuous maternal health prediction. Both Decision Tree Regressor and Random Forest Regressor proved to be perfect prediction tools because they achieved an R² score of 1.000, which demonstrated no errors within the data. The verification of both precision and reliability depends on the minimal Mean Squared Error values. Linear Regression attained a perfect model evaluation through its R² score of 1.000 with small MSE while assessing linear relationships among dataset variables. The neural network in MLP achieved results equal to Linear Regression through R² score 0.9999 and small MSE values that demonstrate accurate modelling of complicated relationships. Support Vector Regressor achieved an R² score of 0.956, but it struggled to monitor intricate maternal welfare information because its error assessment proved unfavourable compared to linear and tree-based methods. The evaluated outcomes from tree-based methods and linear regression demonstrate suitability in developing automated maternal care systems for mental health diagnosis.

**Table 4:** Performance metrics of regression models for mental health prediction.

Model	Mean squared error (MSE)	*R*^2^ score
Random forest regressor	4.5767 × 10^−5^	1.000
Decision tree regressor	6.3581 × 10^−5^	1.000
Support vector regressor	0.0099	0.956
Linear regression	2.3140 × 10^−25^	1.000
Multilayer perceptron	1.6351 × 10^−5^	0.9999

Results highlight the exceptional predictive power of tree-based models and traditional linear methods for regression tasks, reinforcing their reliability for maternal health monitoring.

**Fig. 9:**
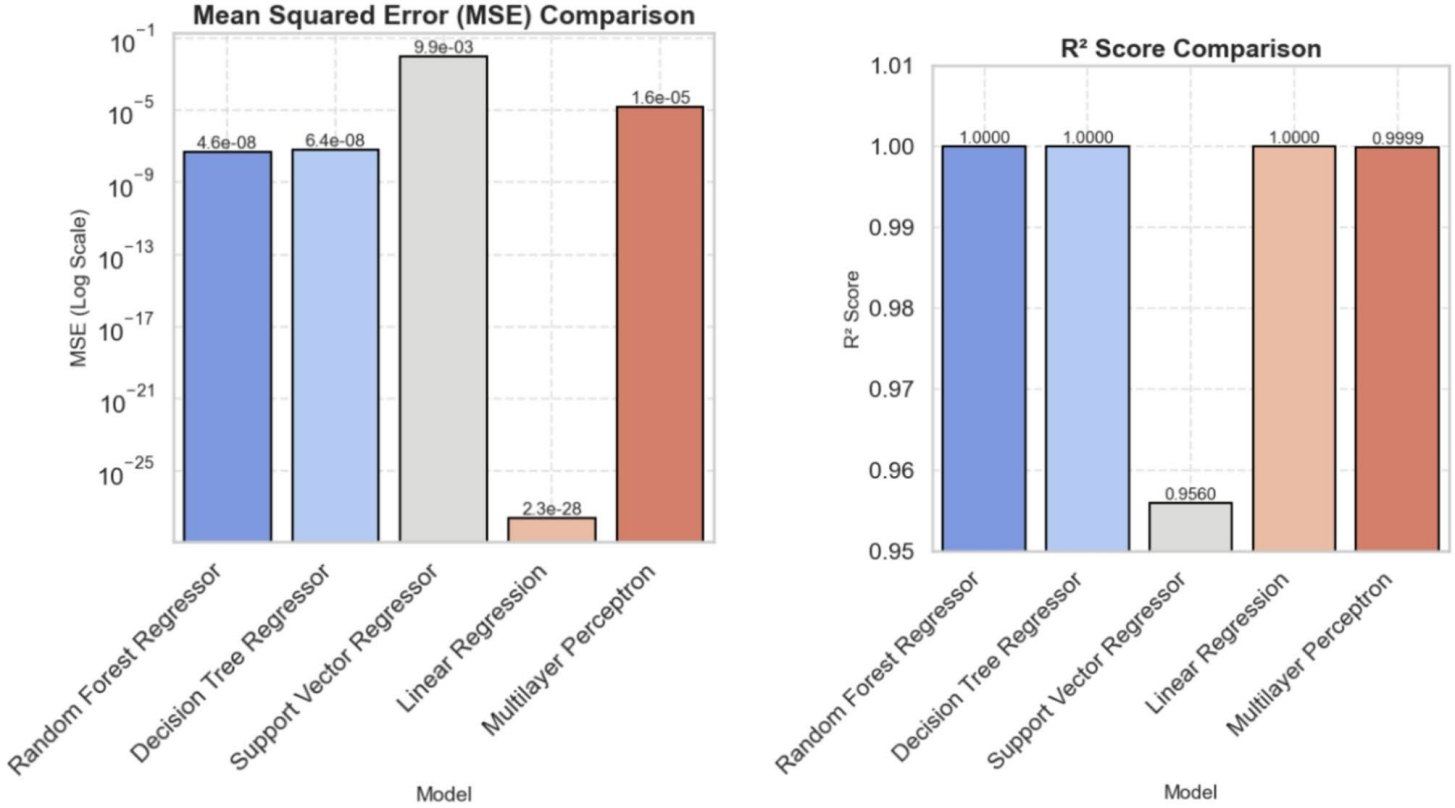
Mean squared comparison and R2 score comparison.

Figure 9 provides a clear visual comparison of the Mean Squared Error (MSE) and R^2^ scores across different regression models used for mental health prediction. The exceptionally low MSE and perfect R^2^ scores of the Random Forest Regressor (4.5767 × 10^−8^, R^2^ = 1.000) and Decision Tree Regressor (6.3581 × 10^−8^, R^2^ = 1.000) demonstrate their superior predictive capabilities and perfect fit to the training data. Linear Regression also achieves a perfect R^2^ score, but with a nearly negligible MSE, suggesting its effectiveness for linear relationships. The Support Vector Regressor shows the worst MSE (0.0099) so far, indicating a certain complexity in learning a more complicated pattern of data, but otherwise, it is also doing quite well with the R2 value of 0.956. The Multilayer Perceptron, finally, works nearly perfect (MSE = 1.6351 × 10^−5^, R2 = 0.9999), which proves that neural networks can be successfully applied to regression tasks as well. The experimental analysis shows that Random Forest model performed better than other models in both classification and regression tests. Due to its resistance, high precision, and low error rates, it can be considered a feasible instrument in developing AI-based solutions in the psychological support of mothers. These findings outline the importance of applying state-of-the-art machine learning algorithms to detect and address psychological health problems at the early stages of pregnancy. Having decided on the performance of the model, we offer a special loss function that allows focusing on the problem of imbalance between classes and achieving even better results in terms of predicted accuracy. The study applied a certain loss functional that considered the requirements of decreasing the imbalance of classes and the quality of performance. There are two important operational features of the function maximize model performance:

1. Cross-entropy loss (CE loss): During classification operations, Cross-Entropy Loss (CE Loss) serves as the primary criterion for assessing forecasting accuracy between expected outcomes and actual class targets. The penalty system’s operational mechanism encourages the model to generate fewer errors and increase classification accuracy rates.
2. F1 score penalty: A special part of the model design, the F1 Score Penalty prevents dominant class bias and shields underrepresented infrequent categories. Complete loss magnification occurs for the model when the F1 score stays low, making the model search for optimal precision-recall balance.

The union of these individual components forms a practical, balanced loss function. The model maintains consistent performance by preventing errors while upholding the fair treatment capabilities of all classes due to this method. The proposed loss function leverages the synergy between accurate results and a balanced distribution of classes, making it a highly effective tool for generating fair and dependable predictions. The Execution of the custom loss function leads to an explanation of training procedures, together with described asset reduction patterns.

Table 5 details the model training process throughout 10 epochs, where it displays the training loss pattern together with related observations. The model indicated a progressive decline in loss values, starting from epoch 1 (2.5880) and reaching epoch 8 (2.4382), which signifies that the model learned effectively while improving its parameter optimisation. The model reached its learning peak during epoch 8 when loss was at its minimum point, indicating it achieved balanced class results and minimum misclassification rates. At epoch 9 (2.4564), loss starts to climb, which means the beginning of overfitting behaviour because the model trains specifically for training data points without adequate generalisation. The loss value from epoch 10 (2.4724) demonstrates possible stabilization that creates a balanced performance by preventing excessive overfitting of the model. A correctly managed training approach becomes visible through this pattern, which proves the model’s capability to handle class imbalance while providing strong performance results across diverse mental health indicators. Verification of these training results proves the model’s universal application competence needed for clinical maternal mental health evaluation.

**Table 5:** Training loss and observations across epochs.

Epoch	Training loss	Observation
1	2.5880	High initial loss, model starts learning
2	2.5342	Moderate reduction, effective parameter tuning
3	2.4987	Consistent improvement
4	2.4823	Steady decline in loss
5	2.4731	Balanced performance across classes
6	2.4608	Reduced misclassifications
7	2.4496	Approaching optimal performance
8	2.4382	Best performance, lowest loss recorded
9	2.4564	Slight increase, possible overfitting
10	2.4724	Stabilized loss, local minimum achieved

**Fig. 10:**
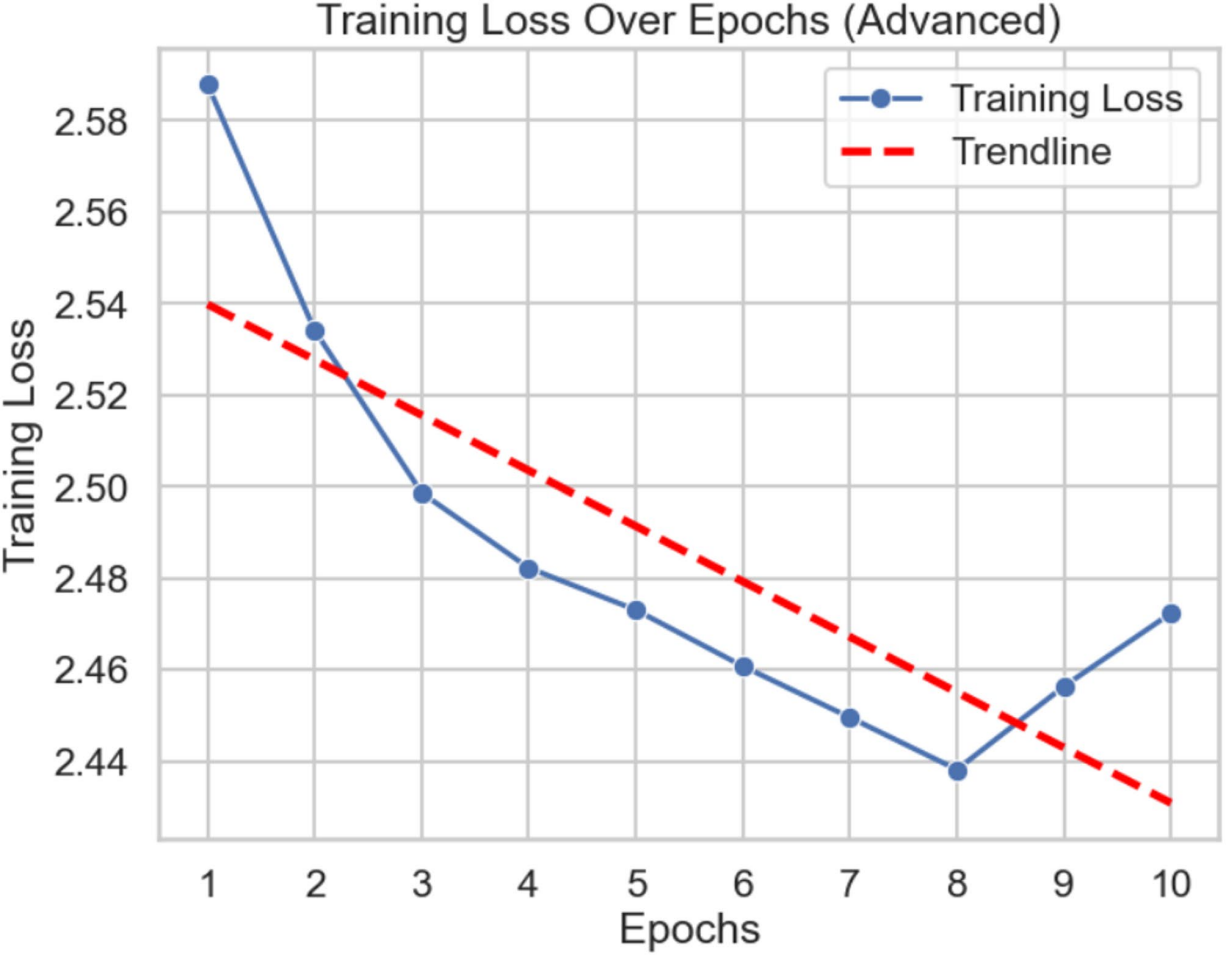
Advanced training loss curve.

Figure 10 provides a clear visual comparison of the Mean Squared Error (MSE) and R^2^ scores across different regression models used for mental health prediction. The exceptionally low MSE and perfect R^2^ scores of the Random Forest Regressor (4.5767 × 10^−5^, R^2^ = 1.000) and Decision Tree Regressor (6.3581 × 10^−8^, R^2^ = 1.000) demonstrate their superior predictive capabilities and perfect fit to the training data. Linear Regression also achieves an ideal R^2^ score, but with a nearly negligible MSE, suggesting its effectiveness for linear relationships. The Support Vector Regressor, while still performing well with an R^2^ of 0.956, shows a higher MSE (0.0099), indicating some difficulty in capturing complex data patterns. The Multilayer Perceptron also achieves near-perfect performance (MSE = 1.6351 × 10^5^, R^2^ = 0.9999), highlighting the strength of neural networks for regression tasks. These results collectively underscore the efficiency and reliability of tree-based and linear models in maternal health monitoring, as shown by the loss values mapped against epoch numbers on their two axes. The time-based loss pattern analysis indicates successful model parameter learning, as the loss decreases over time. The custom loss function successfully handles class imbalance and implements both cross-entropy loss and an F1 score penalty function system. The model training process optimises performance for minority class examples based on loss measurements between epochs, according to research data. Further regularization methods and extended training duration may be necessary because the loss curve exhibits minimal variations throughout the later epochs. The implementation of cross-entropy loss together with an F1 score penalty function makes it possible to enhance imbalanced data management and creates an advanced evaluation framework. Upcoming research will study better methods to adjust loss function parameters as well as advanced approaches to maximize performance potential^23^.

**Fig. 11:**
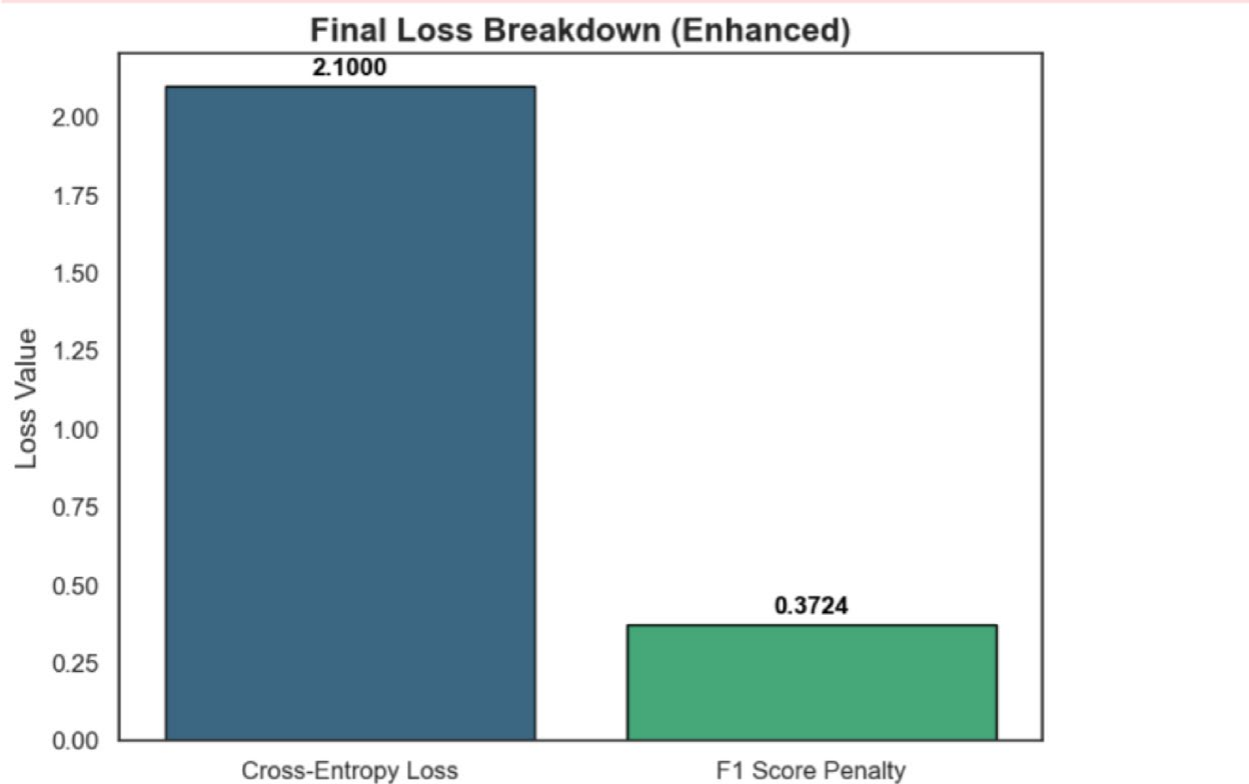
Final loss breakdown (enhanced).

Figure 11 displays loss value distribution against epoch counts across its two axes after the analysis. The time-based analysis of the chart indicates that the learned model parameters follow a downward trend. The implemented custom loss function addresses class imbalance issues by combining cross-entropy loss with an F1 score penalty function. The cross-entropy loss reaches a stable point at 0.3724 during epoch 21,000, which indicates successful optimisation. An analysis of loss data across training epochs demonstrates how the model optimisation method achieves its best performance for processing minority class examples. Later epoch tallies in loss measurement indicate potential overfitting needs, which demand more regularisation methods or longer training duration. The model builds on the evaluation methods and is more effective in dealing with imbalanced data using its F1 score penalty and cross-entropy loss. To achieve improved execution results, the further investigation will consider more effective approaches to enhance the elements of the loss function when developing new tactics. according to the conclusions made during the training sessions we are now going to discuss all the outcomes. The loss was rapidly decreased during the initial steps of the model, which suggests the maximum performance and strong abilities to adjust parameters. The model had been doing its best between the majority and minority classes until the end of the training process in the mid-training epochs. On the final training steps, the model experienced a small rise in loss, probably due to local minimum stabilization or overfitting. Even though the issues related to the class imbalance are minimized, and the model performance is enhanced with the help of a custom loss function, further convergence optimization could be achieved with the help of learning rate adjustments and regularization strategies. Following technical analysis, we do offer our extensive tool that would facilitate maternal mental health. Mental health status is the foundation of wellbeing during pregnancy. The mental stress experienced by expectant mothers leads to anxiety, emotional tension and depression, which adversely affect the developing baby and the expectant mother. Such symptoms can be aggravated by circumstances caused by medical circumstances, physical discomfort and emotional strain. Chronic conditions occur when psychological challenges throughout pregnancy are unchecked and unattended to. Mental health monitoring technologies particularly in pregnancy are desired and useful.

Non-invasive and highly practical mental health tracking technologies concentrate on pregnancy and are needed. Because yoga and natural therapies have proved to be effective in the management of stress and anxiety, they can be part of comprehensive mental health screening systems. Our mental health tracker supports the psychological well-being of pregnant women with directions on yoga training, natural medications, and symptom recording. The latter aims to identify symptoms and offer preventive care, which are the core objectives of this tool. Mood tracking and psychological tests of the mental health tracker, as well as tailored support interventions, allow keeping mental balance at a stable level throughout the pregnancy. The many attributes the tool monitors include detecting depressive thoughts and sadness (Feeling Sad), mood swings and emotional turbulence (Irritability), assessing the quality of sleep and insomnia (Trouble Sleeping), focusing problems (Problems Concentrating), stress-related eating behavior (Overeating), anxiety levels and situations that cause them (Feeling Anxious), feelings of guilt (Feelings of Guilt), maternal bonding problems (Problems Bonding with Baby), and automated alerts in case of high-risk behavior such as suicide attempts. Each characteristic causes customized suggestions, including yoga asanas and herbal remedies. Specific recommendations are drinking lavender or chamomile tea to calm anxiety and breathing exercises along with meditation. Warm milk and aromatherapy with essential oils are also recommended to people who cannot sleep, as well as yoga in Shavasana and Childs Pose. Dynamic mood tracking, Dark Mode, and an intuitive design of the tracker also lead to greater user satisfaction. The program simplifies the data by bar-graph representation of mental health statistics. The 31-day mood visualization allows individuals to be more self-aware and pin down things that may disorder their emotional balance. The application may be developed in future to perform machine learning on previous mental health trends, provide real-time consultation with a medical professional, and offer AI-based mental health assistance capabilities. The proposed changes will provide specialized and narrowed solutions. The prenatal mental health tracker is an innovative test to comprehensive mental health assessment and enhancement of pregnant women in an inviting, scientific manner. This program will offer the user the power to be in control of their mental wellbeing, with the capacity to keep track of crucial metrics and provide preventative treatment alternatives such as yoga and natural remedies.

**Fig. 12:**
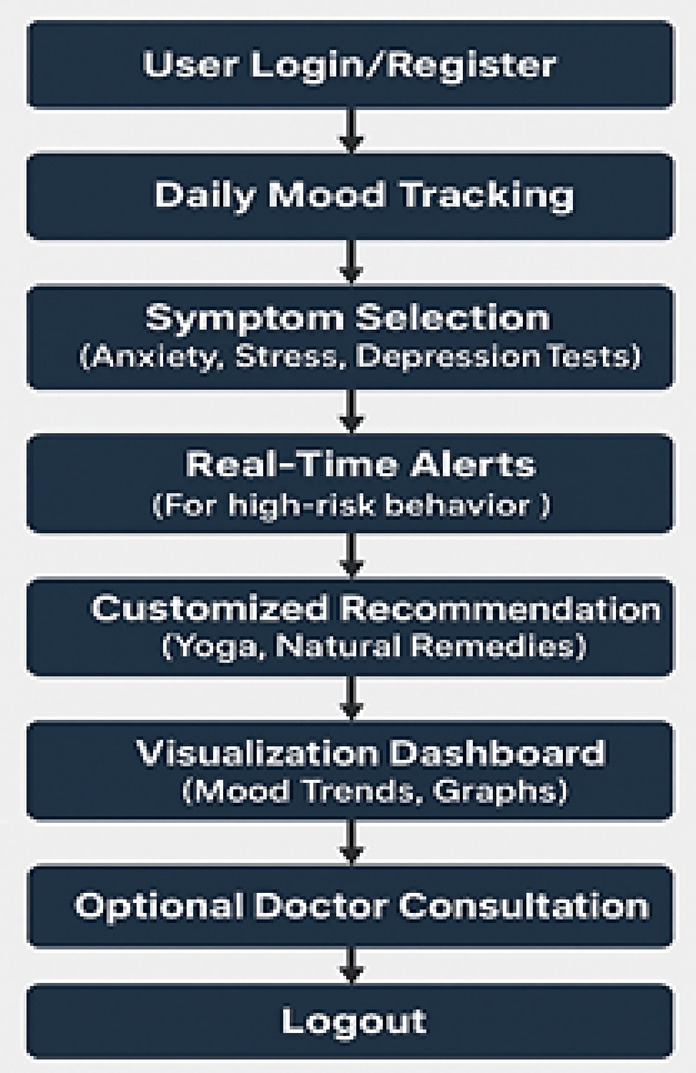
Mental health tracker system.

**Fig. 13:**
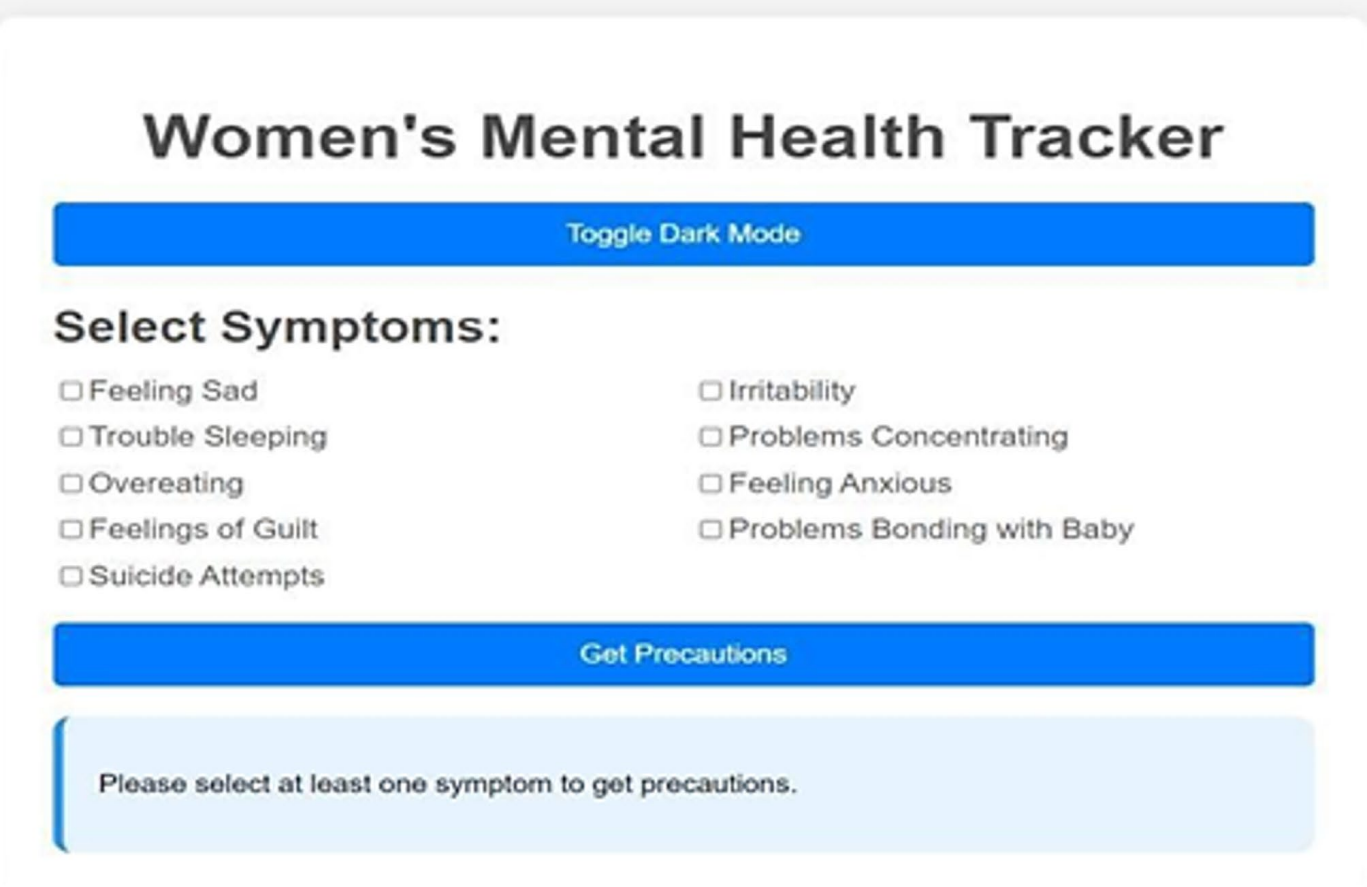
Symptoms selection.

**Fig. 14:**
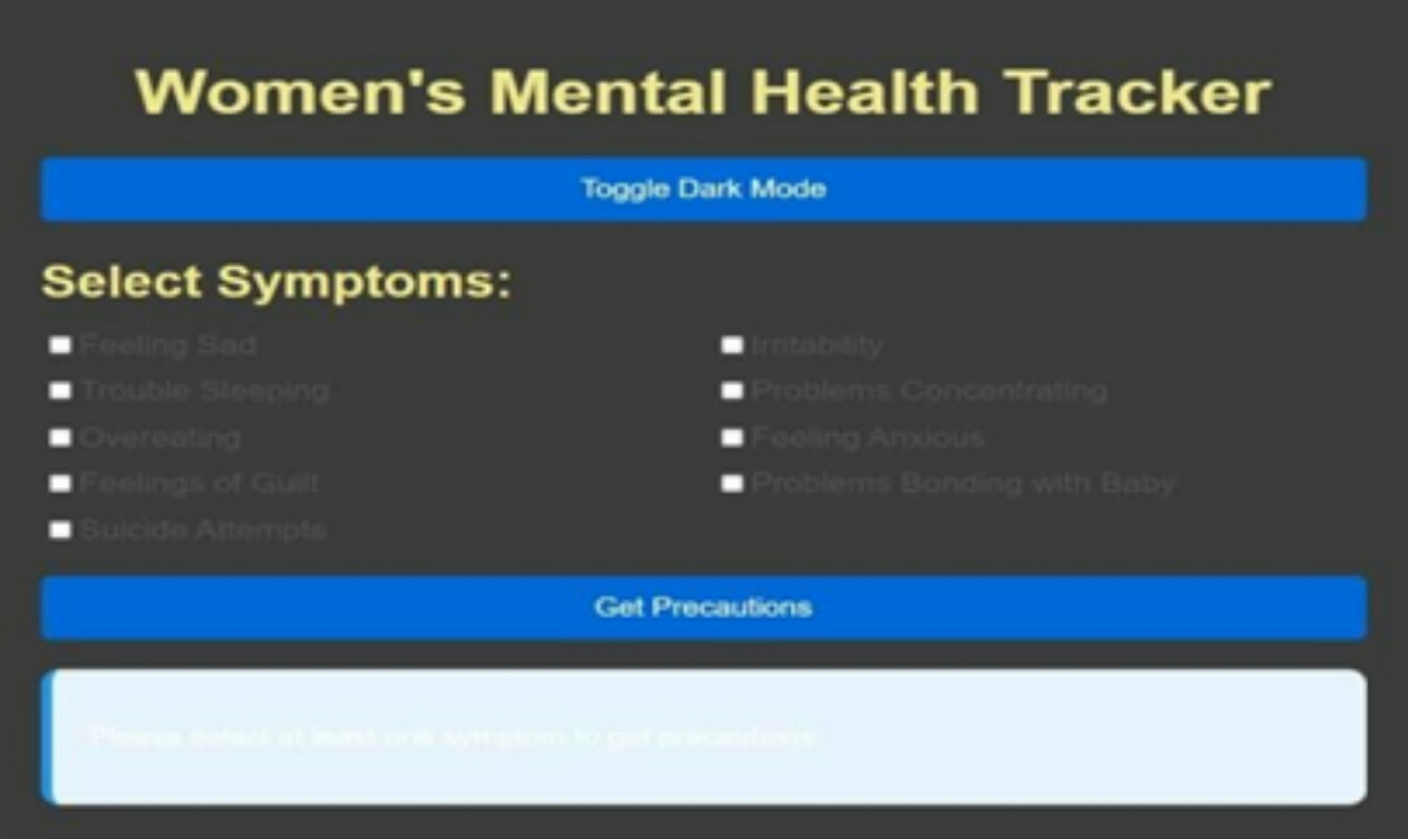
Showing toggle dark mode.

The Mental Health Tracker System, designed for pregnant women to track and improve their mental health, is shown in Fig. 12. Weekly mental health evaluations, bar graph visualizations, and daily mood tracking are all included in the system’s user-friendly interface. By skilfully illustrating mood swings over a 31-day span, the bar graphs help users spot trends and pinpoint possible emotional causes. Through daily check-ins, users can record symptoms like worry, impatience, and difficulty sleeping. The system also provides individualized wellness recommendations based on the recorded symptoms, such as yoga poses and natural cures. Overall, by offering concise, evidence-based insights regarding changes in mental health throughout pregnancy, this tool promotes self-awareness and preventive care (Figs. 13, 14).

**Fig. 15:**
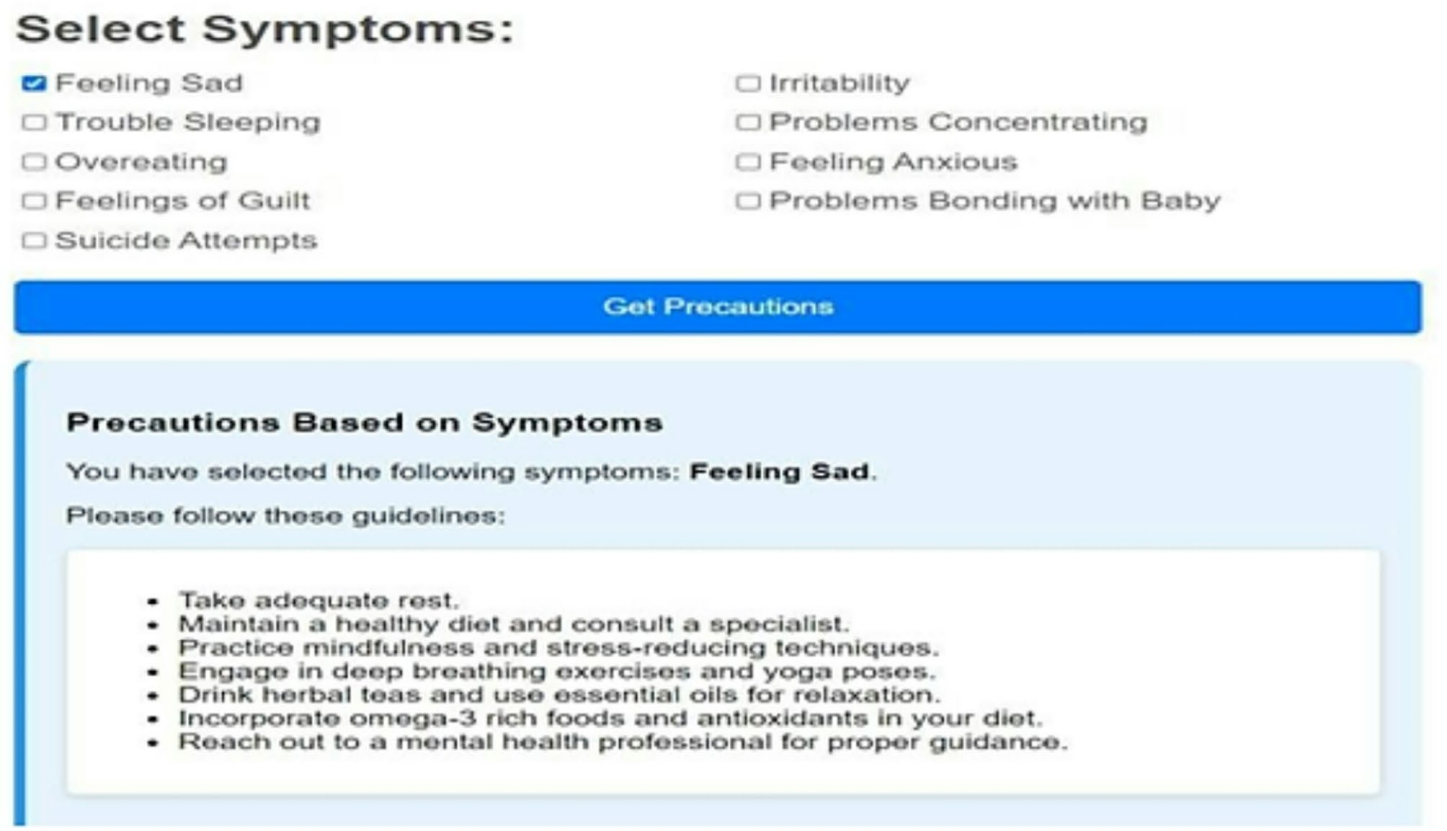
Showing precautions based on symptoms.

**Fig. 16:**
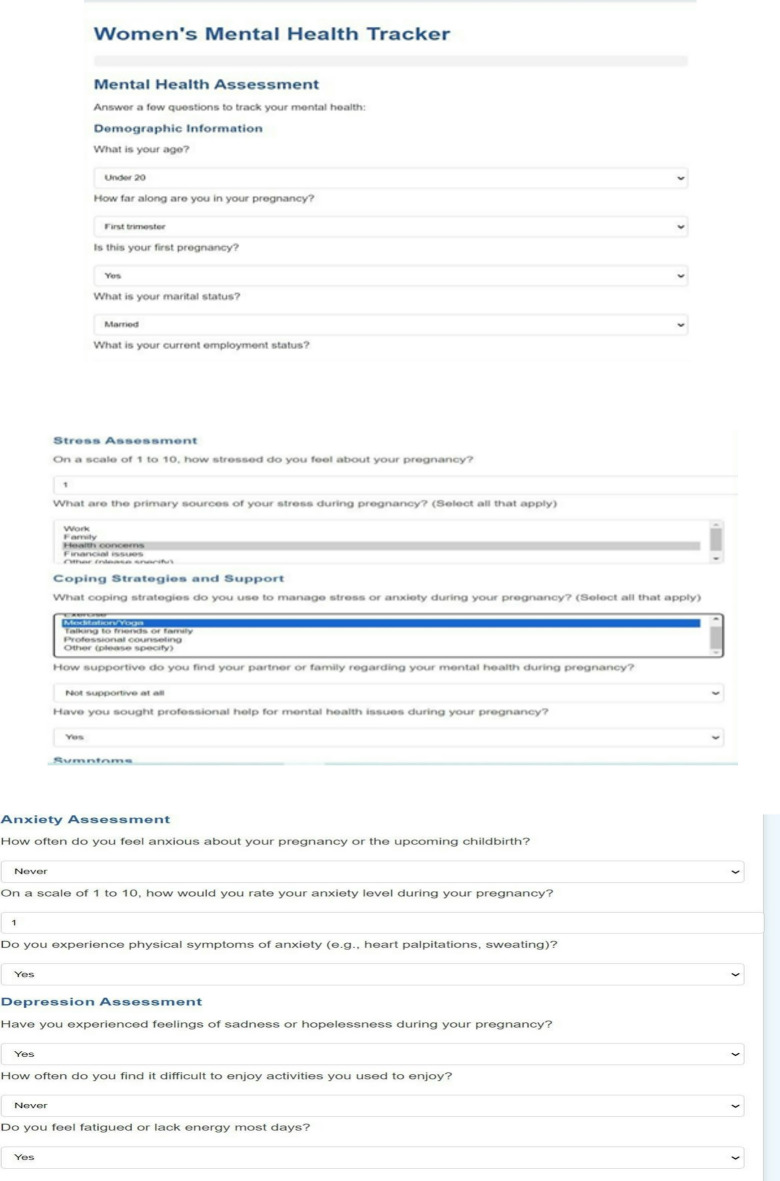
Mental health assessment of anxiety, stress, depression.

**Fig. 17:**
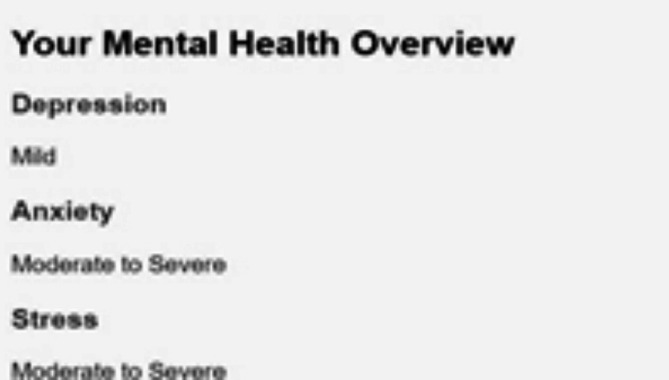
Mental health overview.

**Fig. 18:**
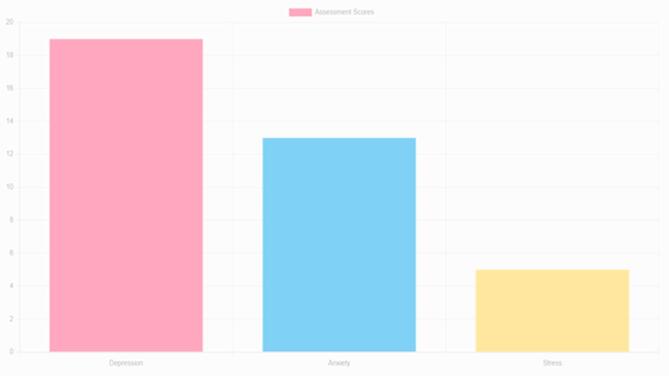
Bar graphs, to present mental health data in a clear and easy-to-understand format.

**Fig. 19:**
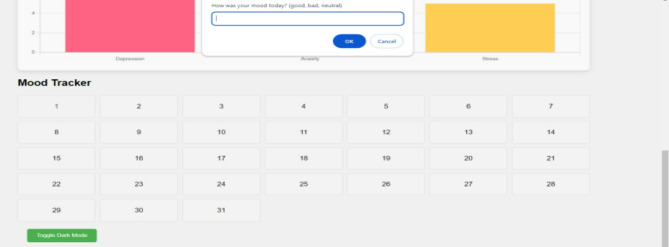
Mental Health Overview, offering users a clear visualization of their mental health trends over time.

The images can be used together to provide a comprehensive view of how the system operates, with options of personalized precaution (Fig. 15), mental health assessments (Fig. 14), and symptom selection (Fig. 13). Such visual elements on the system include toggle dark mode (Fig. 16), bar graphs (Fig. 17), and mental health overviews (Figs. 18 and 19), which enable users to monitor the changes in their emotional conditions. The visualizations provided by this system provide an easy-to-use way of doing data-driven mental health promotion. By providing the solid framework of combining the state-of-the-art machine learning processes with the helpful tool of enhancing maternal mental health, the study is paving the way to the future important breakthroughs in the related area. The project progressed, with an initial design, through the development of an operational system prototype, written in Python, and running on a secure internet platform. The Random Forest classifier was trained on annotated data, and after passing systemic testing with synthetic user data, without compromising privacy of the data, pre-clinical technical feasibility was attained.

To work efficiently, the system requires a digital basis that implies a flexible frontend and backend infrastructure, secure cloud storage services provided by AWS and Azure, and the possibility of instant information management with secure authentication mechanisms. In order to ensure the sustainability of operations in three critical areas, including software development, management of data privacy, and research on artificial intelligence, different professions ought to come up with multidisciplinary teams. The deployment meets the local data protection regulations including the ethical institutional regulations, the HIPAA standards, and the GDPR principles. The encryption of secure health information is facilitated by built-in consent procedure tools that provide users with encryption as well as auditing privileges. The explainable AI modules and protection features of this platform allow end users and medical staff to comprehend AI outputs. The platform will automatically create a model retraining procedure when it receives user and clinical feedback on the platform when the feedback process is activated. The system establishes immediate safety alert messages to medical staff regarding patients with suicidal behaviours and severe psychological issues, thus facilitating fast appropriate clinical attention.

Core machine learning principles receive in-text definitions within the text through precision and recall concepts connected with F1-score and ensemble learning definitions while providing source citations for foundational AI content. The document uses diagnostic criteria from both the World Health Organization (WHO) and the American Psychological Association (APA) together with clinical guidelines to show mental health problems in pregnant women. The expert-reviewed studies confirm that yoga and naturopathy approaches successfully reduce both pre-and-postnatal anxiety and stress and depression symptoms The researchers apply transfer of disciplinary knowledge by using statements such as “From an AI standpoint…” and “Clinically, this implies…” throughout the text The updates seek to establish a better comprehension connection between research scientists and clinical staff and policymakers who work together with integrated healthcare specialists.

### Experimental setup: integration of naturopathic and yoga interventions

Pregnant women face rising mental health challenges because stress, anxiety, depressive symptoms and sleep problems and emotional dysregulation affect various groups of expectant mothers. Medical professionals favor complementary non-invasive treatments as alternatives to conventional drugs because these options pose reduced risks to fetal development. The strategy incorporates naturopathic and yoga-based practices, supported by scientific evidence from peer-reviewed research publications.

Medical therapies from Noruo therapy can work together with yoga methods to give patients full biopsychosocial healthcare, which addresses physical needs along with neural requirements and mental wellness. Pregnant individuals who practice prenatal yoga, incorporating pranayama techniques and mindfulness meditation, can achieve control over their HPA axis and decrease cortisol production, while increasing their parasympathetic response. Maternal emotional strength benefits from combined physiological effects through which fetal development remains protected, along with maternal health improvement and decreased risks of fetal structural abnormalities and early pregnancy complications.

The combination of natural Ashwagandha supplements, aromatherapy, and dietary advice helps patients achieve the most effective results in terms of hormone stability, enhanced sleep quality, and balanced neurochemical levels. The interventions create agreements for personalized pre-birth care objectives dedicated to pregnant women. The following table presents an extensive research summary of high-impact studies which validate the effectiveness of yoga and naturopathic techniques for maternal populations.

**Table 6:** Evidence-based naturopathic and yoga interventions for maternal mental health.

Intervention	Study/source	Maternal benefit reported	Scientific mechanism and outcome
Prenatal yoga	Villar-Alises, O., Martinez-Miranda, P., & Martinez-Calderon, J. (2023)^24^	Reduced depression, anxiety	Modulates cortisol and serotonin; improves vagal tone → Mood enhancement, stress relief
Mindfulness meditation	McKee, K., Admon, L. K., Winkelman, T. N. A., Muzik, M., Hall, S., Dalton, V. K., & Zivin, K. (2020)^25^	Improved emotional regulation, reduced stress	Enhances neural plasticity and self-regulation → Lower anxiety, improved resilience
Pranayama breathing	Feligreras-Alcalá, D., Frías-Osuna, A., & Del-Pino-Casado, R. (2020)^26^	Decreased anxiety, lower heart rate	Boosts parasympathetic activity → Enhanced calmness and emotional stability
Dietary naturopathy	Abrar, A., Fairbrother, N., Smith, A. P., Skoll, A., & Albert, A. Y. K. (2019)^27^	Lower risk of perinatal depression	Omega-3 fatty acids and folate enhance serotonin and reduce inflammation → Improved mood

Table 6 introduces an evidence-based model to support the implementation of yoga and naturopathic interventions within maternal mental health care. Various interventions listed in the table have received high-quality peer-reviewed approval which demonstrates their physiological and psychological and emotional advantages for pregnant women. The research used experimental tests that united machine learning algorithms with yoga methods and naturopathic treatment frameworks, which have scientific validation. The systematic combination leads to an expanded system which enables individuals to create drug-free and satisfactory remedies for pregnancy mental health concerns. Healthcare systems must develop standardised guidelines through randomised controlled trials and meta-analyses, and long-term follow-up evaluations for these practices to become operational. Impact of Natural Treatments on Maternal Mental Health: The health condition of pregnant mothers significantly affects their emotional state as well as the well-being of their newborns. Natural therapies provide the consumer with comprehensive methods, which are not surgical in nature and which help in dealing with stress and anxiety as also emotional balance. The following are evidence-based natural therapy methods, which enhance maternal mental health:

1. The quality of sleep and reduction of anxiety are the twofold benefits of this relaxation technique of putting feet in lukewarm water that works by enhancing blood circulation.
2. The massage therapy on pregnant women instills less stress and a calm mind on the expectant women due to the physical alleviation of pain and lowering cortisol level.
3. Walking barefoot enables the body to ground on the surface of nature either by touching grass or sand thus reducing the level of stress and as a result enabling one to connect with nature better.
4. When listened to, therapeutic music re-enforces moods and reduces anxiety and fear and also establishes an emotional contact with the developing baby.
5. Breathing exercises and pranayama have a calming effect on the mindROM because they regulate the autonomic nervous system and enable emotional balance and mental focus. The research studies have shown that the combined health benefits that come along with the use of several natural therapies are more superior than the use of each separate one.

Studies have shown that natural therapies are most effective when used to treat patients together as the efficiency of a certain form of treatment becomes lowered when applied in the isolation of other forms of treatment. Among the most intriguing ones, the following have been found: Prenatal massage, music therapy, and dietary changes create a significant effect, stressed-management and reduction of anxiety. This is the period that the benefits of probiotics in mental health are manifested hence intake of the good bacteria in the diet leads to postnatal emotional stability. The Ghost Studying therapeutic techniques, which are applied to eliminate the tension and the anxiety, which pregnant women experience, requires the invention of breathing techniques. Over the decades, pranayama has proved itself as a yoga technique of stress reduction by virtue of imparting therapeutic effects that are not limited to oxygenation transparency and mental relaxation. The old breathing techniques and the energy management techniques stimulate the neurological system to provide temporary psychological results.

1. Hand Stretch Breathing enables one to achieve physical and mental relaxation as the hands are moved according to the breathing patterns. Exercise which comprises of deep breathing exercises results in a complete release of imminent stress and tension. The inner calmness is the result of mental clarity and attention to the breath which can be felt already during the practice of movements with the attention to the breath.
2. Nadi Shodhana stabilizes the body through Alternate Nostril Breathing, which operates by controlling respiration to manage heartbeat functions. The practice of education generates mental serenity and body relaxation, making it an excellent therapy for emotional control and anxiety management.
3. Brahmari (also known as Humming Bee Breath) requires you to make hum sounds while exhaling, which leads to physical body vibration, which brings peace to both your mind and nervous system. The vagus nerve activation from hearing humming sounds reduces stress while providing deep relaxation to the audience. The practice of building effectiveness needs to be performed 5 to 7 times.

Figures illustrate the stages and techniques of these breathing exercises, providing a visual guide for effective practice:

**Fig. 20:**
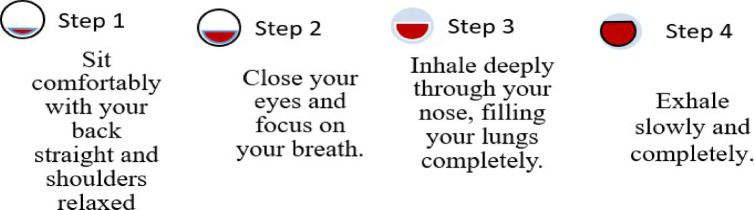
Stages of yoga breathing.

**Fig. 21:**
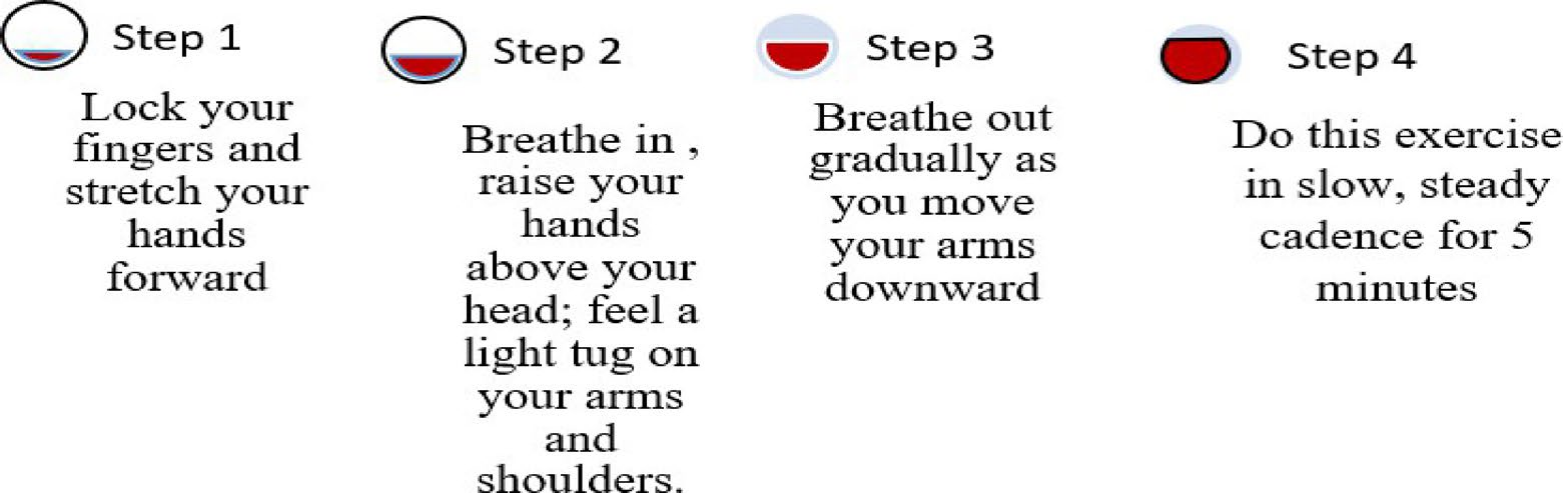
Hand stretch breathing stages.

**Fig. 22:**
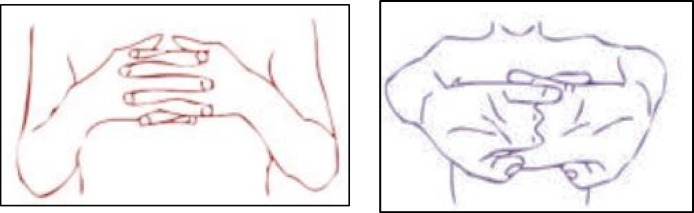
Hand poses during hand stretch breathing—(**a**) Breath in, (**b**) Breath out.

**Fig. 23:**
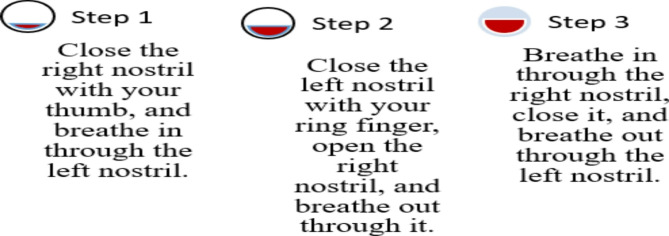
Nadi Shodana breathing stages.

**Fig. 24:**
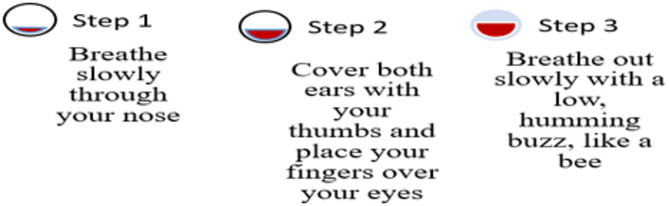
Brahmani pranayama stages.

Figures 20, 21, 22, 23 and 24 depict several types of yoga breathing exercises, including Hand Stretch Breathing, Nadi Shodhana, and Brahmari Pranayama. Each technique delivers stress reduction, mental serenity, and relaxation while presenting an overall method to boost well-being.

Mudras for Stress Relief: Hand positions known as mudras draw from yoga philosophy to restore five body elements (water, fire, earth, air, and space) thus creating stress relief for pregnant women. The recovery of both physical and mental harmony leads to optimised energy circulation while reducing stress. The following five hand gestures serve pregnant women well:

1. Through Prana Mudra practice, women can gain vitality and enhance their immune system function.
2. The Varuna Mudra works on body water levels to establish emotional stability, together with physical health benefits.
3. Gyan Mudra: Enhances concentration, wisdom, and mental clarity.

The application of Vayu Mudra controls air element balance to minimize anxiety along with restlessness in pregnant women.

**Fig. 25:**
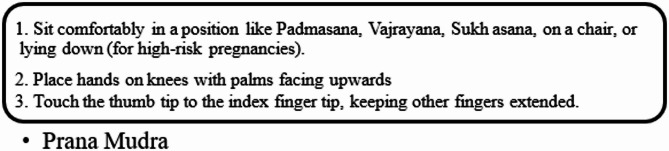
Hand poses during hand stretch breathing—(**a**) Breath in, (**b**) Breath out.

**Fig. 26:**
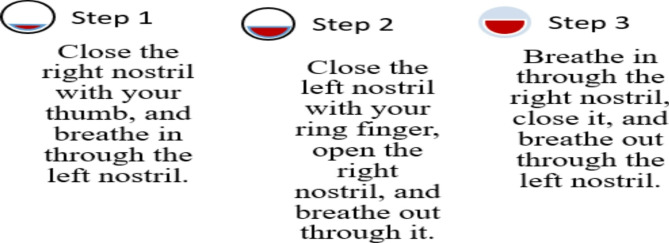
Nadi Shodana breathing stages.

**Fig. 27:**
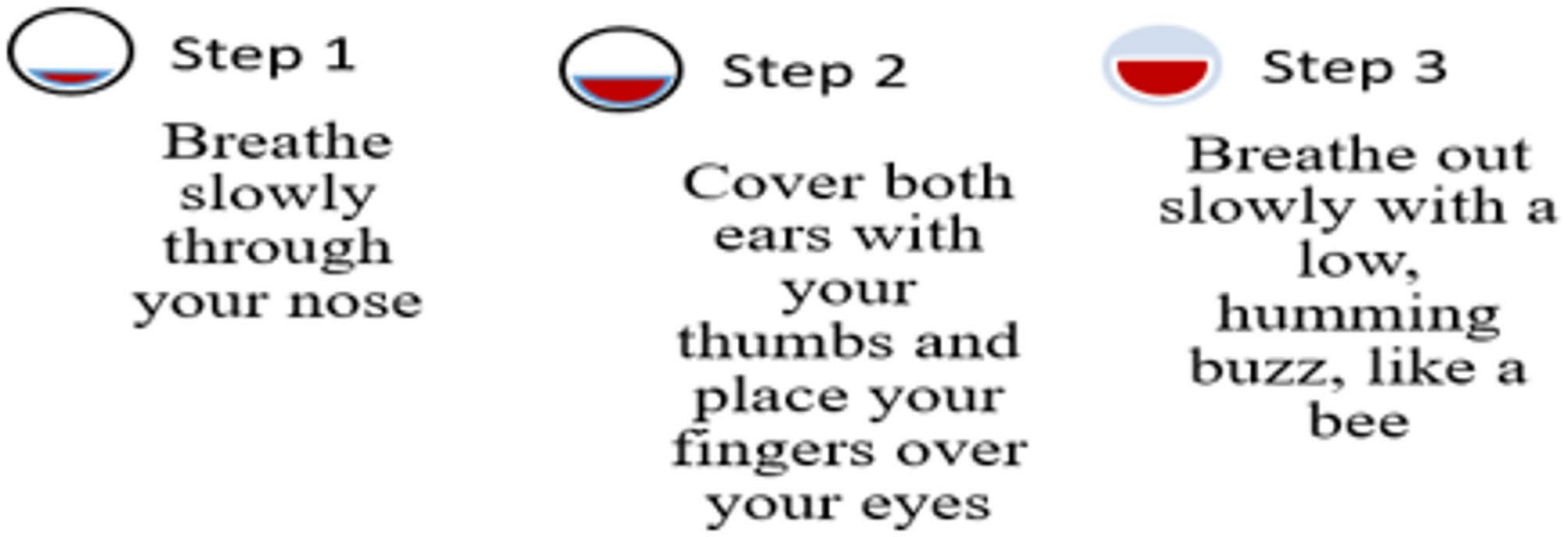
Brahmani pranayama stages.

Yoga breathing methods are shown in Fig. 25 through for Hand Stretch Breathing together with Nadi Shodhana in Fig. 26 and Brahmari Pranayama shown in Fig. 27. These breathing techniques lead users to relaxation while reducing stress together with promoting tranquillity in the mind so users attain well-being in a complete way. Mudras as hand gestures originating from yoga teachings facilitate the balance of body elements (water, fire, earth, air and space) which enables stress relief. The balance established through these techniques stimulates energy circulation for better stress reduction. The following collection of mudras serves pregnant women the most benefit:

1. The Prana Mudra strengthens both immune health and vitality in a person’s body.
2. The Varuna Mudra regulates water elements in your body to establish emotional stability and physical balance.
3. Gyan Mudra: Enhances concentration, wisdom, and mental clarity.

The air element balancing mechanism in Vayu Mudra helps expectant women reduce their nervousness and calm their restlessness.

**Fig. 28:**
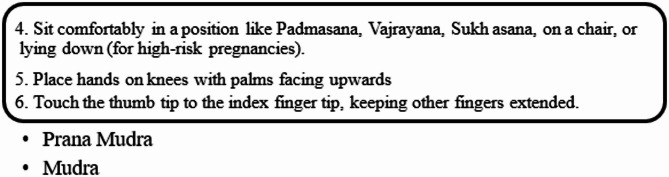
Prana mudra and Varuna mudra benefits.

**Fig. 29:**
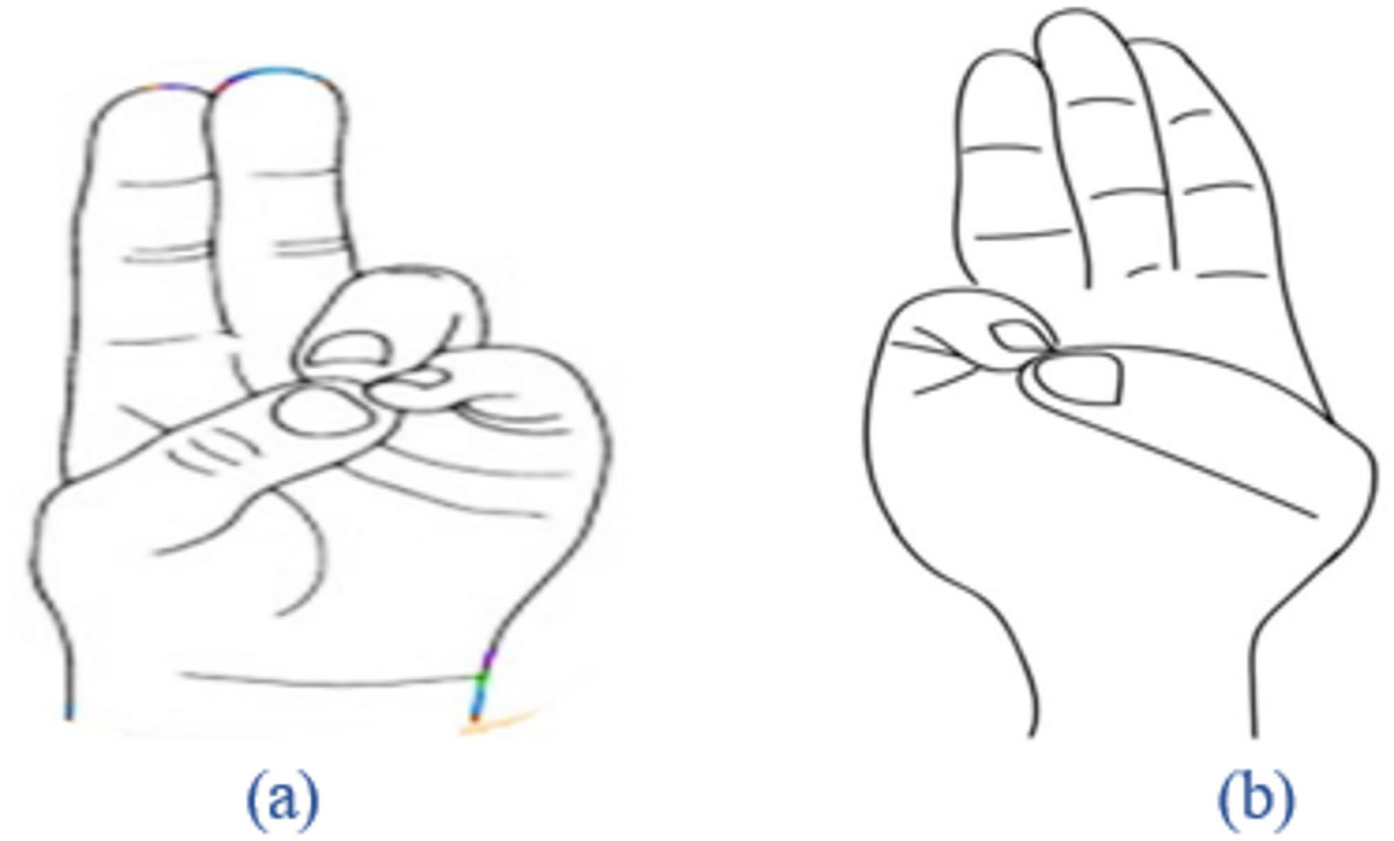
(**a**) Prana mudra, (**b**) Varuna mudra.

**Fig. 30:**
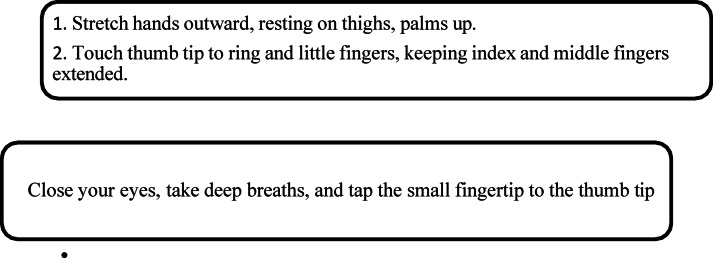
Gyan mudra and Vayu mudra.

**Fig. 31:**
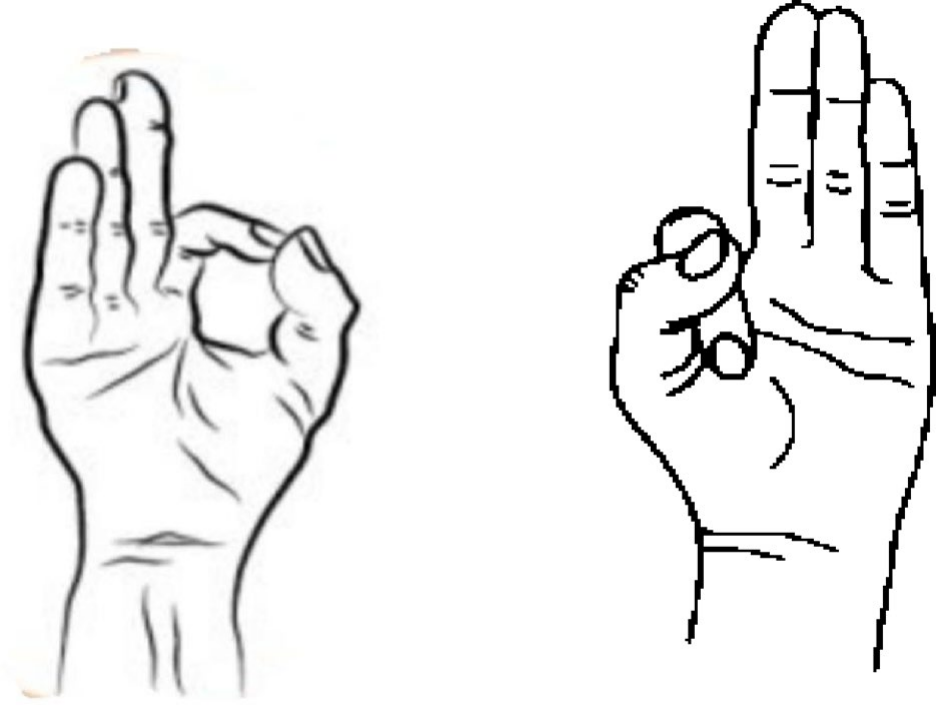
Gyan mudra and Vayu Mudra.

The benefits of instruction of Prana Mudra and Varuna Mudra appear in Figs. 28 and 29, followed by advantages and usage information of Gyan Mudra and Vayu Mudra presented in Figs. 30 and 31. Mudra practices, along with prenatal massage, yoga breathing, and therapeutic music, can help preserve maternal mental health through modified diets and barefoot walking activities. Combining all wellness system elements delivers quick relaxation and sustains positive emotional outcomes, which benefit both mothers and their developing babies. The conclusion of our analysis consolidates all observed effects resulting from the adoption of natural healing techniques. A comprehensive analysis of practical healthcare obstacles in treating psychological conditions in pregnant women would strengthen the research. Additional expansion of the three main topics is necessary. Practical Challenges: Providing psychological aid to expecting mothers presents multiple execution difficulties. Pregnant individuals encounter three significant challenges to mental health care access: pregnancy stigma, low availability of trained mental health experts, together with financial barriers, and cultural pressures. The manuscript will gain both practice-relevant content and enhanced practical value through open discussion of the mentioned issues. Potential Solutions: The manuscript lacks an assessment of possible solutions which address the encountered obstacles. The digital landscape contains mobile health applications along with telemedical services that enable pregnant women from underserved locations to obtain mental health care more easily. Interdisciplinary relationships between mental health specialists and obstetricians, and technologists should be actively developed to deliver a comprehensive therapeutic framework for patients. Pregnant women would benefit from community-integrated support systems, which include peer support groups as well as online forums to create a stigma-free environment.

## Conclusion and future work

This study demonstrates that ensemble machine learning models, specifically those utilising a Random Forest classifier, achieve outstanding results in detecting psychological health changes among expectant mothers. Random Forest classifier resulted in exceptional prediction accuracy at 97.82% ± 0.03% when compared to traditional methods and reached 100% recall and 96.81% ± 0.02% F1-Score. The Support Vector Machine (SVM) proved its worth by achieving 93.79% accuracy and 100% recall to accurately recognize negative situations. The ensemble methods exceeded the accuracy reached by Decision Tree and Logistic Regression models, who achieved comparable results at 91.82% and 91.79%. The experiment revealed moderate accuracy results from Gaussian Naive Bayes and Multilayer Perceptron (MLP) which achieved rates of 93.79% and 92.79%, respectively. The regression tasks demonstrated exceptional success when using the Random Forest Regressor and Decision Tree Regressor, as they produced R^2^ scores of 1.000 alongside minimal prediction errors, reflected in their very low MSE values. The evaluation of other models like Linear Regression, Support Vector Regressor, and MLP Regressor demonstrated their potential for psychological healthcare management systems through high R^2^ scores and low MSE. The study presents an innovative mental healthcare monitoring solution which generates personalized expectant mother care through the fusion of artificial intelligence models alongside natural remedies and yoga practices. The system facilitates immediate data assessment and assists medical personnel in making decisions supporting maternal mental health through data-driven strategies. The predictive power of the models, particularly the Random Forest classifier, supports effective early intervention and proactive healthcare management. The study introduces novelty through using an assessment approach which merges classification and regression methods to evaluate maternal mental health. The research demonstrates that Random Forest functions as the best ensemble method for medical clinical work, as per its findings. A medical environment gains advantages from this model by increasing the performance during repeated runs, along with customized loss functions to manage class unbalance while demonstrating overall reliability. The harnessing of practical clinical potential for this framework requires additional improvements before its implementation in hospital environments.

Real-time data collection via biological and psychological measuring tools will advance through the subsequent stage of development with stress markers, sleeping patterns, and emotional indicators tracking. The development of maternal health will use wearable devices with health trackers to monitor ongoing health conditions while automatically initiating medical interventions. The achievement of better results from classification methods to regression outputs needs improved methods for model learning. Several parameter and loss function modifications with improved regularization features make up the basis of model optimisation against overfitting. During multicasota analysis, the refined versions of the model generate better outcome results because of their new development features. The effective combination of Explainable AI (XAI) technology will become indispensable for building clinician trust in prediction systems. XAI provides decision-making transparency, enabling healthcare professionals to understand the prediction methodology and the rationale behind specific recommendation outputs. A visual explanation system incorporating tools will help medical professionals understand model choices, thereby boosting their trust in AI use for critical healthcare applications.

RCTS, along with continued research, will generate more substantial proof regarding the framework’s performance^31^. Long-term tests will validate the proposed system by evaluating the combined benefits of machine learning models and naturopathic and yoga-based treatments for broader clinical applications. Personalised Healthcare: This platform can provide tailored treatments through continuous health check processing^32^. By carefully selecting interventions within the system, Bellatrix pairs them with individual maternity patient needs to deliver better maternal mental health outcomes^33^. The improvement of maternal mental health AI technologies requires the foundation of essential research for future development. A maternal mental health management system builds a comprehensive healthcare operation by integrating predictive analytics, real-time data analytics, and machine learning algorithms^34^. Explained - AI technology combined with real-time data updates made possible by better data quality controls will enable this system to predictively support mental health care for pregnant women across the world.

## Electronic supplementary material

Below is the link to the electronic supplementary material.

Supplementary Material 1

## Data Availability

The data that support the findings of this study were collected from the Department of Gynecology, Majidia Hospital, Jamia Hamdard, India. However, restrictions apply to their availability. These data were used under license for the current study and are not publicly accessible. However, the data can be obtained from the authors upon reasonable request and with permission from Majidia Hospital, Jamia Hamdard, India.
