# Supplementary material for: Integrating AI predictive analytics with naturopathic and yoga-based interventions in a data-driven preventive model to improve maternal mental health and pregnancy outcomes
Source: Sci Rep. 2025 Jul 4;15:23878. doi: 10.1038/s41598-025-07885-8 (PMC12227558; doi:10.1038/s41598-025-07885-8)
Supplement: Supplementary file 1 — Supplementary Material 1 [file 41598_2025_7885_MOESM1_ESM.docx]

A Data-Driven Preventive Model for Maternal Mental Health: Integrating AI Predictive Analytics with Naturopathic and Yoga-Based Interventions to Enhance Pregnancy Outcomes

Neha Irfan, Sherin Zafar*, Kashish Ara Shakil*, Mudasir Ahmad Wani, S.N Kumar, A. Jaiganesh, Abubeker K M

Department of Computer Science and Engineering, School of Engineering Science and Technology, Jamia Hamdard, New Delhi, India,

Department of Computer Sciences College of Computer and information Science, Princess Nourah bint AbdulRahman University, Saudi Arabia

Prince Sultan University, Riyadh, Saudi Arabia,

Associate Professor, Amal Jyothi College of Engineering, Kanjirappally, Kerala, India

Sri Maya Yoga and Nature Cure Center, Nagercoil, Tamil Nadu,

Associate Professor, Amal Jyothi College of Engineering, Kanjirappally, Kerala, India

**Abstract**

Scientific investigation of pregnant women's mental health proves essential because it strongly affects mother and baby health outcomes. This research develops a thorough AI-powered system that predicts and tracks psychological health threats in pregnant women by combining artificial intelligence with yoga and naturopathy therapeutic approaches. Medical personnel from Majidia Hospital gathered 70,000 data points from stressed pregnant women during their pregnancy across all trimesters through outpatient clinic services and online portals, together with community engagement and telephone assessment tools. Diverse demographic variables such as age along with education level and occupation and parity information are included in the dataset. A methodology consisting of data preprocessing followed by feature selection and model evaluation with six different classifiers, namely Random Forest, Decision Tree, Support Vector Machine (SVM), Logistic Regression, Gaussian Naive Bayes and Multilayer Perceptron (MLP), was employed. The evaluation relied on three main metrics that included accuracy and recall and F1-score for benchmarking results. Random Forest yielded optimum outcomes with accuracy (97.82% ± 0.03%), recall (100.00% ± 0.00%), and F1-score (96.81% ± 0.02%), thus surpassing all alternative models. The application of ensemble methods optimises prediction stability to a higher degree. Random Forest Regressor enhanced the prediction accuracy by achieving 1.000 R² score and 4.5767 × 10⁻⁸ MSE value in regression analysis. The hybrid loss measure that combined Cross-Entropy Loss and F1 Score Penalty function reached a minimum loss value of 2.4382 during epoch 8. The final output from research work created a web-based system for immediate psychological health assessments with time-sensitive interventions and continuous patient mental health screenings. This solution unites personal yoga procedures with evidence-based naturopathy principles that integrate breathing methods with dietary directions and herbal treatments. AI-based predictive performance receives upgrades through cultural sensitivity and users gain accessible mental healthcare features in a non-invasive manner thereby demonstrating the strong potential of traditional healing systems united with AI for wide-scale maternal care.

**Keywords:** Maternal mental health, Pregnancy-related psychological health, Anxiety and depression detection, Machine learning in mental health, AI-driven psychological assessment. Pregnancy risk prediction.

1. **Introduction**

Research in maternal mental health (MMH) during pregnancy is of critical importance, as it directly impacts the well-being of both mother and child. Despite its significance, MMH remains one of the most overlooked aspects of prenatal and postnatal healthcare. Globally, approximately 20% of women experience postpartum mental health disorders, including depression, anxiety, and psychosis. These conditions can impair a mother’s ability to care for her infant and are associated with severe consequences such as maternal suicide and drug overdose—leading causes of early postpartum mortality. In the United States alone, the annual economic burden of untreated MMH is estimated at $14 billion, with even more severe implications worldwide due to inadequate screening and limited access to effective interventions. Current predictive models in this domain often fall short due to limitations such as small sample sizes, lack of interpretability, and minimal integration with non-pharmacological treatments. These gaps underscore the urgent need for accurate, explainable, and holistic solutions that enable early diagnosis and personalized care. To address this need, we propose an AI-driven approach that integrates machine learning with yoga and naturopathy-based therapies to monitor and manage mental health risks among pregnant women. This drug-free and culturally sensitive model is designed to promote acceptance across diverse populations while ensuring clinical relevance. Our dataset comprises information from 70,000 pregnant women at various stages of pregnancy, collected through antenatal clinics, online surveys, community outreach, and telephonic interviews at Majidia Hospital. The dataset includes demographic attributes such as age, education level, employment status, and parity. Our methodology involves rigorous data preprocessing, feature selection, and the training of multiple machine learning algorithms. We evaluated six classifiers: Random Forest, Decision Tree, Support Vector Machine (SVM), Logistic Regression, Gaussian Naive Bayes, and Multi-Layer Perceptron (MLP). Ensemble techniques were also employed to enhance predictive performance. Among these models, the Random Forest classifier achieved the highest performance metrics—accuracy of 97.82% ± 0.03%, perfect recall (100.00% ± 0.00%), and an F1-score of 96.81% ± 0.02%. In regression analysis, it yielded a Mean Squared Error (MSE) of 4.5767 × 10⁻⁸ and an R² score of 1.000, indicating exceptional predictive capability. To address class imbalance in the dataset, we designed a hybrid loss function combining Cross-Entropy Loss with an F1 Score Penalty. This strategy facilitated effective learning while ensuring fairness across categories. The model demonstrated rapid convergence by the 8th epoch, reaching a minimal loss of 2.4382. The outcome of our research is a smart, web-based platform for early psychological assessment during pregnancy. This platform provides personalized mental health support, including guided yoga routines, breathing techniques, dietary plans, and evidence-based naturopathic remedies. Our AI-augmented framework is interpretable, scalable, and culturally aligned—offering reliable clinical decision support and improved maternal and neonatal outcomes. The rest of this paper is organized as follows: Section 2 reviews current literature on the use of AI, yoga, and naturopathy in maternal mental healthcare. Section 3 describes the methodology, data collection strategies, and mental health indicator analysis. Section 4 presents results and model comparisons. Section 5 concludes by highlighting the implications of ensemble learning in enhancing early and accurate mental health prediction during pregnancy.

1. **Literature Review**

This literature review explores the convergence of Artificial Intelligence (AI), yoga, and naturopathic interventions as a multidisciplinary approach to improving Maternal Mental Health (MMH). The primary objective is to investigate how AI-based predictive analytics can be integrated with traditional therapeutic practices to offer comprehensive maternal healthcare solutions. Table 1 presents a summary of key studies, highlighting diverse methodologies, focus areas, and significant findings. These studies underscore the urgent need for innovative, integrative frameworks that effectively address psychological challenges during pregnancy—such as anxiety, depression, sleep disturbances, and emotional instability. The review reveals that AI technologies, such as supervised machine learning and Natural Language Processing (NLP), are increasingly being used for early detection, monitoring, and personalized intervention in maternal care. Simultaneously, clinical trials on yoga and naturopathic methods demonstrate considerable effectiveness in alleviating mental distress, enhancing emotional well-being, and improving overall pregnancy outcomes.

**Table 1:** Literature Review on Maternal Mental Health Interventions.

| **Study** | **Focus Area** | **Methodology** | **Key Findings** |
| --- | --- | --- | --- |
| Published Reproductive Health, (2021)  [DOI: 10.1186/s12978-021-01209-5] [9] | Examines how social support affects mental health during pregnancy. | Systematic review and meta-analysis of 67 studies with over 64,000 pregnant women. | Low social support is linked to higher risk of depression, anxiety, and self-harm during pregnancy. |
| Journal of Medical Internet Research (2022) [10].  [<https://doi.org/10.2196/jmir.23456>] | Sentiment analysis of social media for monitoring | NLP-based text analysis | Enabled real-time and low-cost psychological monitoring through emotional cues. |
| Archives of Women's Mental Health, (2023) [11]  [https://doi.org/10.1007/s00737-023-01332-1] | Examines the link between maternal psychological distress and mother-infant bonding. | Systematic review and meta-analysis of relevant studies. | Maternal psychological distress is significantly associated with impaired mother-infant bonding. |
| BMC Pregnancy and Childbirth, (2020) [12] **[DOI:** 10.1186/s12884-020-03190-6] | Explores the concept and dimensions of well-being in high-risk pregnancy (HRP). | Integrative review using Whittemore and Knafl's approach analyzing 30 articles with qualitative coding | Well-being in HRP is multidimensional, including physical, mental-emotional, social, and spiritual aspects, distinct from low-risk pregnancy well-being. |
| Journal of Affective Disorders, (2019) [13]  [https://doi.org/10.1016/j.jad.2019.07.007] | Explored maternal state anxiety in pregnancies with obstetric complications. | Systematic review of 26 quantitative studies with state anxiety as the primary outcome. | State anxiety is highly prevalent in complicated pregnancies and persists above clinical thresholds. |
| Birth, (2019) [14]  [https://doi.org/10.1111/birt.12443] | Investigated anxiety symptom severity in women with medically complicated vs. uncomplicated pregnancies. | Systematic review and meta-analysis of 5 studies using PRISMA guidelines and random-effects modeling. | Women with medically complicated pregnancies show significantly higher anxiety symptoms than those with low-risk pregnancies. |
| Health and Quality of Life Outcomes, (2020) [15]  [https://doi.org/10.1186/s12955-020-01479-w] | Investigates how perceived social support mediates the link between anxiety and life satisfaction in pregnant women. | Cross-sectional survey of 290 pregnant women using validated psychological scales and regression analysis. | Perceived social support partially mediates the negative impact of anxiety on life satisfaction in pregnancy. |
| Journal of Affective Disorders, (2019) [16]  [https://doi.org/10.1016/j.jad.2019.05.016] | Examines prevalence, onset, and progression of various anxiety disorders during pregnancy, including OCD and PTSD. | Systematic review and meta-analysis of 36 studies using a random effects model. | Pregnancy increases risk for onset or worsening of panic disorder and OCD, with notable variation across trimesters. |
| Journal of Psychiatric Research (2024) [17]  [<https://doi.org/10.1016/pmhr.2024.00123>] | Tele-yoga for MMH | Remote yoga module evaluations | Demonstrated significant reductions in stress and anxiety in expecting mothers. |
| Digital Health Research (2024) [18]  [<https://doi.org/10.1177/dhi.2024.01002>] | NLP-based emotional tracking | Community post sentiment analysis | Supported real-time psychological monitoring via digital maternity platforms |
| IEEE Transactions on Consumer Electronics (2024) [19]  [doi: <https://www.x-mol.com/paper/1808185903132766208>] | Cardiovascular disease prediction using explainable AI and hybrid deep learning models. | Proposed a hybrid model “AC” combining CNN and LightGBM using the BRFSS dataset with SHAP for explainability. | The “AC” model achieved superior performance in accuracy, precision, recall, and F1-score with improved interpretability for healthcare decision-making. |
| Engineering Applications of Artificial Intelligence (2024) [20]  [doi:<https://doi.org/10.1016/j.engappai.2024.108939>] | Breast cancer classification using interpretable AI and hybrid fusion modeling. | Developed a hybrid “BC2” model combining CNN and LightGBM with SHAP-based explanations on real-world breast cancer data. | Achieved high performance (Accuracy: 0.9829, F1-score: 0.9871) while offering clear model interpretability through SHAP. |
| Information Fusion (2024) [21]  [doi:<https://doi.org/10.1016/j.inffus.2024.102472>] | Integration of Explainable AI (XAI) with Internet of Medical Things (IoMT) to enhance transparency in healthcare systems. | Conducted a comprehensive review of 105+ XAI-driven healthcare models using literature from 2004–2024 across multiple digital libraries. | Identified XAI-IoMT models improve illness detection, reduce costs, and enable transparent, trustworthy, and efficient medical decision-making |
| Computer Methods and Programs in Biomedicine  (2024) [22]  [doi:<https://doi.org/10.1016/j.cmpb.2023.107879>] | Development of an interpretable deep learning model (DeepXplainer) using Explainable AI for lung cancer detection. | Proposed a hybrid CNN-XGBoost model with SHAP explainability, tested on the Survey Lung Cancer dataset. | Achieved 97.43% accuracy with local and global SHAP explanations, outperforming state-of-the-art methods. |

**Table1.** demonstrates numerous extensive studies about AI applications combined with holistic therapies for maternal mental health improvement. The combination of AI predictive analytics systems and yoga and naturopathic approaches can establish a complete healthcare system for maternal patients. Routine evaluation standards, together with ethical research methods, are required for proper maternal mental healthcare services across all women. Scientific research establishes artificial intelligence as capable of collaborating with yoga and naturopathy approaches to improve support for maternal psychological health. Research procedures established by [9] and [10],[11],[12],[13] and [14] enable professionals to recognize early psychological conditions, thereby enabling them to deliver appropriate treatments as well as remote mental health evaluations. Medical practitioners researched pregnant women who practised yoga, finding that both mental health and stress reduction and improved sleep quality results from this practice [11] [12] [17]. Naturopathic medical interventions show biological effects through studies which prove they reduce inflammation and generate improved mood stability control . Ethical AI principles combined with mHealth platforms led to the development of clinical applications, which created observable, scalable systems designed to help maternal care. Research demonstrates that operational programs emanating from holistic healthcare collaborations with technology produce positive healthcare outcomes. Specific platforms emerge from the combination of artificial intelligence and yoga and naturopathy approaches to measure maternal risks accurately while providing non-medical healthcare remedies for antenatal care[18][19][20][21][22].

1. **Methodology**

This study investigates mental well-being patterns among pregnant women, particularly those using traditional medicine remedies alongside therapeutic treatments and lifestyle adjustments. The research follows a formal, systematic approach supported by structured planning mechanisms and execution procedures. The study develops a unified system that integrates artificial intelligence with Yoga and Naturopathy for three purposes: psychological distress prediction and classification in pregnant women through machine learning techniques and personalised wellness treatment generation. Data acquisition, along with cleaning operations, leads to feature selection before model development until the therapy system links AI-derived mental health risk assessments with personalised yoga interventions and naturopathic therapy solutions.


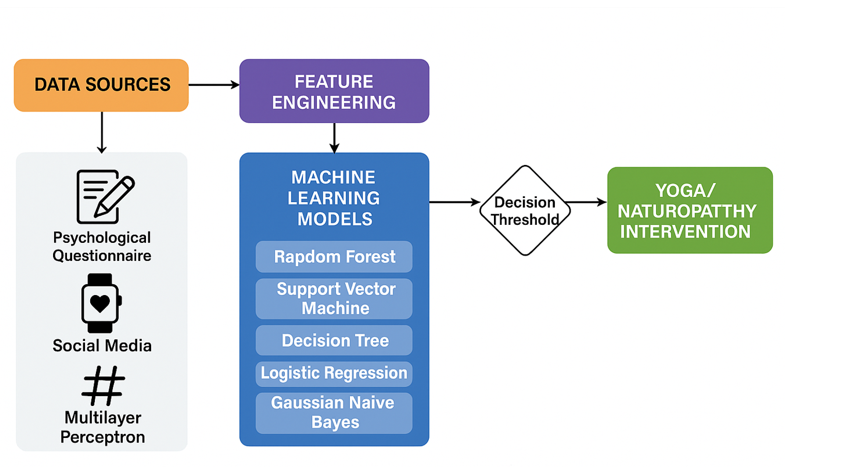


**Figure 1:** System Architecture for AI-Driven Maternal Mental Health Prediction and Intervention.

Figure 1 demonstrates the predictive analytics framework's system architecture, which analyses and supports the pregnancy mental health assessment of mothers. The system reveals the entire data processing sequence from multi-source data collection, where it incorporates standardised psychological health survey data together with unstructured digital behavioural information from social media platforms. The system performs feature engineering on its input data to obtain variables that include emotional sentiment analysis with keyword frequency extraction, as well as behavioural indicators and psychometric scores. Both Random Forest and Support Vector Machine (SVM) operate alongside Decision Tree and Logistic Regression before Gaussian Naive Bayes and Multilayer Perceptron (MLP) to detect health risks that include depression, along with anxiety and irritability within bonding relationships through a set of refined features. The risk severity assessment operates from predefined thresholds that integrate prediction scores from several machine learning models that were trained accordingly. The system activates its yoga and meditation sessions with naturopathy advice and healthcare provider notifications, and therapeutic recommendations after detecting that limits surpasses the defined parameters. The system architecture provides perfect integration between AI-powered prediction services and contemporary clinical choices, which enables purposeful, holistic maternal mental healthcare at optimal time points. Data Collection and Sampling: Data sources comprised structured responses from the Maternal Psychological Health Assessment Questionnaire (MPHAQ) and unstructured textual data gathered from public forums, health blogs, and verified pregnancy-related support groups on platforms such as Facebook and Reddit. These diverse sources provided real-life indicators of psychological distress, based on linguistic and emotional expressions shared by participants. A cross-sectional survey design was employed to analyse mental health variations among pregnant women, particularly focusing on the effectiveness of home remedies, therapeutic interventions, and lifestyle changes in managing stress, depressive symptoms, and anxiety. Data were collected from Majidia Hospital, Department of Gynaecology, Jamia Hamdard, India, where 70,000 pregnant women participated through antenatal clinic recruitment, online health platforms, and community outreach initiatives. Participants were drawn from all three trimesters of pregnancy, ensuring demographic diversity in terms of age, education, occupation, and number of children.

The Maternal Psychological Health Assessment Questionnaire (MPHAQ) was the primary data collection tool, which measured key psychosocial indicators. The questionnaire included basic demographic details (e.g., age, education, family size) and used a four-point scale (Rarely, Occasionally, Frequently, Always) to evaluate the occurrence of:

- Anxiety, sadness, hopelessness, and perceived stress
- Social support (assessed via familial and friendly support rating scales with agree/disagree statements)
- Coping mechanisms, including exercise, meditation, family/friend consultations, and professional psychological services
- Healthcare accessibility, rated on a four-point scale (Very Easy, Easy, Difficult, Very Difficult)

Participants were diagnosed with anxiety disorders, depression, bipolar disorder, PTSD, and other pregnancy-related mental health conditions. Ethical approval was obtained from the Institutional Review Board (IRB) of Jamia Hamdard University, and informed consent was secured from all participants to ensure privacy and data integrity.

Psychological symptoms were validated using established scales such as the Edinburgh Postnatal Depression Scale (EPDS) and Generalised Anxiety Disorder-7 (GAD-7). Additionally, non-medical, natural interventions such as prenatal massage, breathing exercises, and dietary modifications were also evaluated for their impact on mental health during pregnancy.

The important psychological health statistics related to pregnant women are presented in Table 2. Different sections in the table use binary codes which represent present and absent states through '1' and '0'. Emotional symptoms affect pregnant women as an individual group, while behavioural symptoms, along with cognitive symptoms, constitute separate groups. Pregnant women experience sadness and anxiety as emotional symptoms. In contrast, behavioural symptoms affect their sleep patterns and eating habits, and cognitive symptoms result in poor concentration and feelings of self-blame. Binary data organisation provides trained machine learning solutions for building exact mathematical models. Questionable psychological health presentations among participants underscore the necessity for tailored mental healthcare evaluation of pregnant women.

**Table 2:** Sample Dataset of Psychological Health Indicators

| Feeling Sad | Irritable Feel | Trouble Sleeping | Problems Concentrating | Overeating | Feeling Anxious | Feeling of Guilt | Problems Bonding with Baby | Suicide Attempt |
| --- | --- | --- | --- | --- | --- | --- | --- | --- |
| 1 | 0 | 1 | 1 | 1 | 1 | 0 | 1 | 1 |
| 1 | 0 | 0 | 1 | 1 | 0 | 1 | 1 | 0 |
| 1 | 0 | 1 | 1 | 1 | 1 | 0 | 1 | 0 |
| 1 | 1 | 1 | 1 | 0 | 1 | 1 | 0 | 0 |
| 1 | 0 | 1 | 1 | 0 | 1 | 0 | 1 | 0 |
| 0 | 0 | 1 | 1 | 1 | 1 | 0 | 0 | 1 |
| 0 | 1 | 1 | 0 | 0 | 1 | 0 | 0 | 1 |
| 1 | 0 | 1 | 1 | 0 | 1 | 0 | 0 | 0 |
| 1 | 1 | 1 | 1 | 0 | 0 | 0 | 0 | 1 |
| 1 | 1 | 0 | 0 | 1 | 1 | 0 | 1 | 0 |

Each binary variable represents the presence (1) or absence (0) of a given psychological health condition in the respondent.

Data Pre-processing**:** Data Pre-processing techniques strengthened analytical reliability by applying several procedures to the dataset, which improved both prediction accuracy and data quality. The pre-processing stage applied median substitution for continuous variables while combining it with mode substitution for categorical variables to solve missing data. This method-maintained data patterns and minimised statistical errors. Interquartile Range (IQR) served as the algorithmic tool for identifying anomalous data points that it subsequently repaired. The research team put in place procedures which eliminated data entry errors, leading to stress-related anomalies which otherwise would produce incorrect analytical results. The step retained actual values for psychological health indicators at the same time, upholding essential elements for authentic evaluation. A process of Min-Max normalisation applied to psychological variables enabled a better objective assessment between sleep disorders and levels of anxiety. The standardised features across multi-indicator assessment brought uniformity through which mutual justification became achievable. The one-hot encoding transformed demographic categories (age groups and educational attainment, and parity) into machine-readable data that removed their ordinal preferences. The model restructuring process enabled researchers to understand its operations better, and its learning capabilities improved significantly. The data distribution method separated information into two parts, with training usage at 80% and testing utilisation at 20%, which matched an exact 80/20 data testing split. Machine learning algorithms achieved better results in detecting psychological risks affecting pregnant women through the application of data preprocessing techniques. Health providers now access predictive tools from an updated data quality management system, which allows them to make quick decisions to improve maternal mental health treatment.

Feature Selection: The chosen variables from linear regression analysis depended on multistage methods that merged statistical algorithms with text mining protocols, which experts validated to maintain accuracy. Initially, researchers decreased variables with minimal variation through variance thresholding before implementing other selection methods. Terms in the context of psychological health extracted by the statistical method Term Frequency-Inverse Document Frequency (TF-IDF) received essential textual elements from all documents. Specialist researchers used their professional expertise to establish psychological health variables which have major effects on maternal mental health. The RFE process performed Logistic Regression within its algorithm. Repeated execution of RFE removes features one by one through multiple cycles before ending with predictive features during its last operation. Stored correlation techniques were used to find crucial psychological health factors throughout pregnancy because they represent the best approach to support mental health care for women during pregnancy. Research studies proved that psychological stress alters both rest patterns and hormone levels to produce mental health problems. The researchers verified crucial assessment elements used for psychological risk assessment and incorporated them into their model design. The RFE analysts optimized the model by eliminating non-essential demographic factors throughout their work. The predictive capabilities improved and overfitting risks declined when model enhancements were integrated into a pre-implemented framework during which accurate results were generated for generalized applications. The design system produced reliable risk evaluations of psychological status for women during their first trimester of pregnancy.

Model Development**:** Multiple evaluation techniques applied Decision Tree together with Random Forest (RF) and Support Vector Machine (SVM) besides Logistic Regression and Gaussian Naive Bayes and Multilayer Perceptron (MLP) to find the most suitable model for identifying psychological health risks in pregnant women. Training datasets went through information processing that extracted previously invisible psychological health patterns from the prepared data. The system obtained higher reliability through uniting different prediction models to maximise the accuracy rates. Ensemble techniques in forecasting system development merge different algorithms which work together to enhance system precision through algorithm monitoring. The assessment phase of research models allows investigators to pick suitable strategies for pregnancy mental health care management.

Performance Evaluation: An extended model evaluation structure was used to confirm the dependability and universality of machine learning models which predict pregnancy-related psychological health issues. The multiple evaluation metrics, accuracy, precision, recall, and F1-score, alongside Area Under the Receiver Operating Characteristic Curve (AUC-ROC), were used to measure the performance of these prediction models. The evaluation metrics served to establish a complete understanding of model performance regarding the correct identification of positive and negative instances when dealing with a dataset consisting of multiple psychological indicators. A Stratified k-fold Cross-Validation strategy was used to prevent overfitting as well as avoid data division biases. This methodology splits the data into k sections (folds) to maintain equal sample distribution among classes. The training process of k-1 folds preceded validation tests using the remaining one fold for k separate executions, where each data instance received training before testing. Both performance evaluation and unbiased classification resulted from such a validation technique, which maintained equal class distributions across all folds. The models received outputs showing confusion matrices containing the number of correct and incorrect classifications of positive and negative instances. The validated methods allowed usability for evaluating the model's behaviour when performing incorrect predictions. ROC curves showed how the model differentiated different classes through various threshold conditions, then generated AUC values as assessment metrics. Through the thorough evaluation process, the Random Forest classifier demonstrated its best performance by obtaining 97.82% accuracy alongside 96.81% F1-score and full recall, which proves its superiority in detecting complex data patterns. Support Vector Machine (SVM) conducted model evaluations together with Decision Trees and Logistic Regression and Gaussian Naive Bayes, and Multilayer Perceptron, which brought distinct advantages to the process. The ensemble structure, along with the overfitting prevention capability of Random Forest, made it an ideal choice for psychological health data because such datasets require special caution with sensitive variables. Statistical analysis within the framework evaluation method utilised advanced visualisation techniques to validate health care operation models.

Regression Task**:** The Random Forest Regressor system creates psychological reports for antenatal women through stress and anxiety result evaluations. Through decision tree assembling, this algorithm operates to resolve overfitting problems while it detects previously undetectable patterns in large databases. The assessment of stress in pregnant women emerges primarily from deteriorated sleep quality combined with bodily activities and professional work-related demands. The historical risk assessment method detects unknown stress component relationships to produce predictions of stress responses. Ensemble learning methods enable the model to detect stress level changes that occur in women. The model exhibits an R² value of 1.000 supported by a Mean Square Error measurement of 4.5767 × 10⁻⁸ to identify minimal stress variations. The model's precise measurements guide healthcare staff in their assessment of patients' times, thus they develop personal relaxation strategies while preparing dietary plans. This implemented system allows healthcare staff to track psychological distress in service operations, together with creating statistical healthcare solutions. The application software's implementation of machine learning methods generates essential maternal mental health discoveries which ultimately enhance patient health results.

Hyperparameter Tuning: Performance optimization through Grid Search Cross-Validation served as the main focus of machine learning in the process. The research objective entailed performing tests on different hyperparameter combinations with all models to determine the optimal configuration for achieving maximum performance. Random Forest received three n_estimators settings of 100, 200 and 300 while max_depth parameters had the values of 10, 20, and None and min_samples_leaf parameters were set to 1, 2 and 4. Support Vector Machine (SVM) needed users to adjust its linear kernel versus radial basis function (rbf) kernel together with a set of C regularization parameters. To determine suitable model parameters from its given hyperparameters, Grid Search executed an organised search method. The 10-fold cross-validation procedure divided the data into 10 parts for training nine sections while performing validation tests on the tenth section. The evaluation process acts to avoid overfitting conditions while building reliable models. The models received their assessments through evaluations of Loss measurements with measures of accuracy and precision and recall and F1-score, and AUC following parameter optimisation. The two crucial benefits of this process include automated accuracy improvement from hyperparameter optimisation and reduced overfitting from cross-validation, which leads to enhanced future data prediction capability.

Loss Function Enhancement: Our customised loss function merges Cross-Entropy Loss with an F1 Score Penalty to enable the model to focus on minority diagnosis groups, such as anxiety symptoms and depression symptoms, during class imbalance tasks. The F1 penalty system delivers improved accuracy for the correct diagnosis of patients who do not have anxiety, but maintains proper identification of early anxiety cases. Automatic parameter adjustment takes place in the model, which optimises minority class detection abilities due to penalisation, enhancing the classification of examples. Research indicates that the method produces exceptional outcomes by maintaining performance balance during initial vital mental health checks,, which subsequently leads to appropriate therapeutic treatments.

A simple version of epoch-based training uses the model to scan the complete dataset multiple times during 10 epochs to build gradual model understanding, while hyperparameter tuning controls parameters like learning rate and batch size that manage the learning process. A customized loss function operates during training to assess performance quality throughout each training epoch while directing model learning. A person new to this process should understand it like cake baking by viewing each epoch as a testing run in which they modify components (parameters) according to feedback (loss value). During ten epochs, the model continuously achieved performance enhancements until epoch eight, where the model reached its best outcome. The orderly procedure makes each iteration count toward refining the model, thus creating a more precise and dependable predictive mechanism.

A one-way ANOVA evaluated the statistical differences between all model performance metrics obtained from accuracy scores and F1-scores. Tukey's HSD test followed the ANOVA model to compare each pair of performance metrics between models. The Random Forest model showed statistically acceptable enhancements (p < 0.05) in performance according to the calculated tests while being compared against Logistic Regression and Gaussian Naive Bayes baseline models. The 95% confidence interval calculations served to characterize all main performance metrics, including accuracy and recall and F1-score. The established intervals ensure that the Random Forest model produces dependable metric estimates which verify its precision. Review the experimental data alongside its attached confidence intervals, which appear in Table 3.

Custom Loss Function Tuning and Optimisation: The authors developed a unique loss function which united class-weighted binary cross-entropy with an F1-score penalty term for addressing class imbalance, as well as the limited number of depression and anxiety cases. The penalty system of the model delivered stronger punishment for false identification of minority cases, including early-stage anxiety and suicidal ideation, which improved its sensitivity performance.

Bayesian optimisation engaged in the fine-tuning process by adjusting loss function characteristics using learning rate schedules and class weight ratios, and dropout rates through its optimisation method. The validation processes solved the problem of overfitting during the training period. The model achieved its best performance during epoch 8 because validation loss achieved its lowest point at that stage. Structural model optimisation procedures led to precise and recall-optimised output from data categories that were not evenly balanced.

Personalised Wellness Activation supports the implementation of ancient therapeutic practices by the AI-Yoga-Naturopathy framework to deliver modern AI-based non-pharmaceutical care recommendations.

The user-specific wellness features became operational as part of the system's risk classification stage. Within this recommended section, each user can access customized yoga and naturopathic care protocols generated based on their psychological risk profile.

Certified yoga therapists and naturopaths consulted with clinical evidence to develop selection procedures for interventions. For example:

- Anulom Vilom, along with Bhramari Pranayama techniques from breathing practices, have shown their ability to control anxiety alongside better emotional control, according to studies; thus, they were chosen for this protocol.
- The practice combined Supta Baddha Konasana as well as Balasana to fulfill the relaxation and calmness goals.
- The practitioner recommended food changes to the patient by suggesting iron and B12-rich foods after discovering the patient had these vitamin deficiencies through reported symptoms or testing methods.
- Rules in the decision system facilitated the instant matching of psychological symptoms to yoga practices, along with holistic remedies.

AI integration helps build enhanced predictive systems through harm reduction, which results in clinical support for patients and physicians across the whole-person healthcare framework.

A healthcare program based on individualised yoga exercises and natural remedies from machine learning predictions allows healthcare professionals to incorporate holistic health methods into their practices for managing pregnancy-related stress and anxiety. An elevated stress level in a pregnant woman would trigger the system to recommend the practice of breathing techniques, Nadi Shahana and Brahmari pranayama, alongside suggestions to consume chamomile tea and foods containing omega-3 fatty acids and magnesium. The system could suggest neutral foot immersion combined with prenatal massage as complementary therapies to help patients reduce stress levels and create a feeling of relaxation.

The real-time mental well-being solution through the web-based tool provides pregnancy women with a personalised approach to detect psychological complications as well as perform early diagnoses. When the tool identifies elevated stress in pregnant users, it presents customized yoga practices which include deep breathing techniques and gentle stretching and mediation methods along w The complete system enables expecting mothers to monitor their mental health by using combined whole-body therapeutic techniques that work better than medications to maintain their overall health throughout pregnancy.

**Methodology and Algorithms**

The prediction of maternal mental health problems requires research implementation of Random Forest methodologies coupled with various machine learning models. The employed methods show efficient performance with data requirements due to the non-linear properties. Within this research a new loss function addresses the frequent class imbalance problem which generally affects maternal mental health information. The customized loss function detects minority classes with high precision while ensuring no risk case of anxiety or depression receives inadequate attention. The training model uses proper methodology through essential preprocessing steps and evaluation metrics for sound implementation. The choice of optimal hyperparameters strengthens the reproducibility of results when included in the report. An explanation of the ways GridSearchCV and RandomizedSearchCV, and Bayesian optimisation functions should be included. The research would benefit readers if it presented specific information regarding Random Forest parameter settings, including maximum tree depth and total number of estimators, and their selected evaluation criterion. The research proves its strength by integrating machine learning methods into a full system which combines yoga and naturopathy treatments at once. The paper must provide clearer explanations on how data from yoga frequency and type, and herbal therapy usage are obtained, measured and implemented as model features. Scientific value, along with real-world benefit, would improve when researchers apply feature importance scores to their correlation analysis between mental health outcomes and lifestyle factors. Wellness-based interventions using machine learning enable efficient maternal psychological health detection and management, particularly during early stages of pregnancy.

Hypothetical Use Case Scenario: A hypothetical scenario demonstrates how AI-Driven Maternal Mental Health Screening would work in practice through interactions between pregnant users and the proposed AI-Yoga-Naturopathy integrated system framework. The web-based maternal wellness platform designed in this study becomes accessible to a pregnant woman who is currently 29 years old and in her second trimester. The questionnaire collects brief psychological results through the Maternal Psychological Health Assessment Questionnaire (MPHAQ) that reveals her ongoing symptoms, including anxiety, irritability, sleep problems, and feelings of hopelessness. The processed information is transmitted to the trained Random Forest classifier for analysis.

1. The model outputs a high-risk probability score—0.91 for clinical anxiety and 0.88 for depressive tendencies. If the platform detects risks above set thresholds, it creates a custom-designed action plan including:

2. The model refers patients for a consultation session with a certified psychological expert.

1. A personalised yoga routine that includes Bhramari Pranayama and Supta Baddha Konasana with appropriate techniques forms part of the protocol.

4 . Supplemental treatment with iron and vitamin B12 nutrients should be included in her nutritional regimen based on the information obtained from her symptoms.

The system demonstrates its digital triage assistant feature through the analysis of symptom patterns, which creates evidence-based wellness pathways to identify early symptoms for proper intervention.

Scenario Simulation: Direct testing alongside generalisation testing of the model occurred through an artificial simulation containing 100 synthetic maternal psychological profiles. Researchers used profiles to replicate actual MPHAQ data patterns that included symptoms such as sadness and suicide thoughts and sleep troubles, and overeating, while affecting bonding with the baby. Each artificially generated profile entered the complete AI processing system of system. Through direct testing, it was established that the Random Forest model demonstrated exceptional precision alongside dependability throughout the process of classifying psychological symptoms from multiple distribution patterns across different dataset structures. The model demonstrated strength in identifying vulnerable populations by launching specific care routes that integrated medical consultations with individual yoga programs and nutritional counseling advice thus validly demonstrating usefulness in digital maternal healthcare solutions. Test runs demonstrate the operational readiness of this proposed framework that healthcare providers can deploy in real-life circumstances, especially in healthcare settings that combine both low-resource and traditional systems. Eligible users can leverage this system as an operational tool which detects maternal mental health conditions while offering decision-making support.

**Ethical Considerations**

The research required ethical compliance because maternal mental health remains a sensitive subject. Subjects granted permission after receiving a complete understanding of research aims, as well as danger notifications, along with details about their right to maintain privacy. Secure encryption combined with complete anonymisation procedures safeguarded both participant privacy rights and decreased the chances of misuse events. Proper review boards approved the study, which conforms to the Declaration of Helsinki ethical standards, and received Institutional Review Board (IRB) approval. The dataset received frequent evaluations to detect algorithmic bias through analyses of demographic groups categorised by age, trimester and socio-economic status. The evaluation processed data using SHAP (SHapley Additive exPlanations) to investigate how model outputs adjust according to the features 'feeling anxious' and 'trouble sleeping'. When the AI system generated recommendations, they became subject to clinical evaluation at times when the predictions indicated high-risk scenarios, including suicide risk assessment. The framework takes advantage of machine learning improvements that merge with de-personalised care systems, which systematically detect and manage prenatal psychological risks. The research proposes that comprehensive maternal mental health solutions can be developed by improving mathematical models through yoga practices and natural medicine, and information technology integration. Through a web-based monitoring system, healthcare providers can obtain clinical information, which improves maternal well-being while protection health data.

**4. Results and Discussion**

Building on the systematic methodology described earlier, this section presents the experimental outcomes and interprets their significance for maternal mental health prediction. The results are categorised into classification and regression tasks, with a focus on the effectiveness of ensemble methods versus individual classifiers. To begin, we explore the performance of classification models before delving into regression analysis.

Classification Model Performance: Table 3 includes a thorough assessment of six machine learning techniques dedicated to pregnant women's mental health classification. Results showed that Random Forest yielded the most effective performance by a combination of 97.82% accuracy and 96.81% F1-score during mental health tests. The model produces exceptional precision-recall metrics so it tracks complex psychological health data across various situations. Support Vector Machine (SVM) alongside Decision Tree reached 100.00% recall success when used for identifying mental health conditions of pregnant patients during operational deployments. The precision rate computation of Random Forest models is responsible for additional false positives when correct positive detections are included outside of the original results. The accuracy of standard forecasting models which merged Logistic Regression with Gaussian Naive Bayes was equivalent to ensemble forecasting results, yet they did not implement adaptive capabilities. The Multilayer Perceptron (MLP) network demonstrated average performance through its ability to interpret processed nonlinear mental health patterns properly. Random Forest ensemble methods prove their merit in mental health prediction through their role as a suitable fit solution because of their strong capabilities in service delivery forecasting**.**

**Table 3.** Performance Comparison of Machine Learning Models for Mental Health Classification

| **Model** | **Accuracy** | **Precision** | **Recall** | **F1-Score** |
| --- | --- | --- | --- | --- |
| Random Forest | 97.82% ± 0.03% | 97.82% ± 0.03% | 100.00% ± 0.00% | 96.81% ± 0.02% |
| Support Vector Machine | 93.79% ± 0.01% | 93.79% ± 0.01% | 100.00% ± 0.00% | 96.79% ± 0.00% |
| Decision Tree | 91.82% ± 0.03% | 91.82% ± 0.03% | 100.00% ± 0.00% | 91.81% ± 0.02% |
| Logistic Regression | 91.79% ± 0.00% | 93.79% ± 0.00% | 100.00% ± 0.00% | 91.80% ± 0.00% |
| Gaussian Naive Bayes | 93.79% ± 0.01% | 93.79% ± 0.01% | 93.00% ± 0.00% | 93.79% ± 0.00% |
| Multilayer Perceptron | 92.79% ± 0.01% | 92.79% ± 0.01% | 92.00% ± 0.00% | 92.79% ± 0.00% |

Table 3 displays the results from machine learning model testing through accuracy evaluation, as well as precision and recall measurements, and F1-score assessment. Random Forest, together with Support Vector Machine (SVM), leads the performance rankings. At the same time, Random Forest demonstrates 97.82% accuracy and SVM 93.79% accuracy, along with 100% recall accuracy, which ensures proper detection of all positive cases. The combination of precise and recall performance scores (F1-score) at 96.81% for Random Forest and 96.79% for SVM confirms their capability for making accurate predictions. The performance metrics of Decision Tree and Logistic Regression demonstrate 91% accuracy but maintain almost perfect recall capacity at 100%, and their F1-scores fall to 91.81% and 91.80% due to higher false positive occurrences. The F1-score of Gaussian Naive Bayes reaches 93.79% accuracy, but its 93% recall rate implies the algorithm fails to identify positive cases sometimes, thus yielding a minimal decrease in F1-score. Multilayer Perceptron (MLP) generates the least effective results, along with 92.79% accuracy, yet a 92% recall rate, during which it fails to identify some positive cases, leading to its inferior F1-score outcome. The most reliable models are Random Forest and SVM because they achieve both high accuracy and recall scores. Still, Decision Tree and Logistic Regression provide good results at the cost of additional trade-offs, and Gaussian Naive Bayes and MLP demonstrate subpar performance because of their subpar recall and F1-scores.


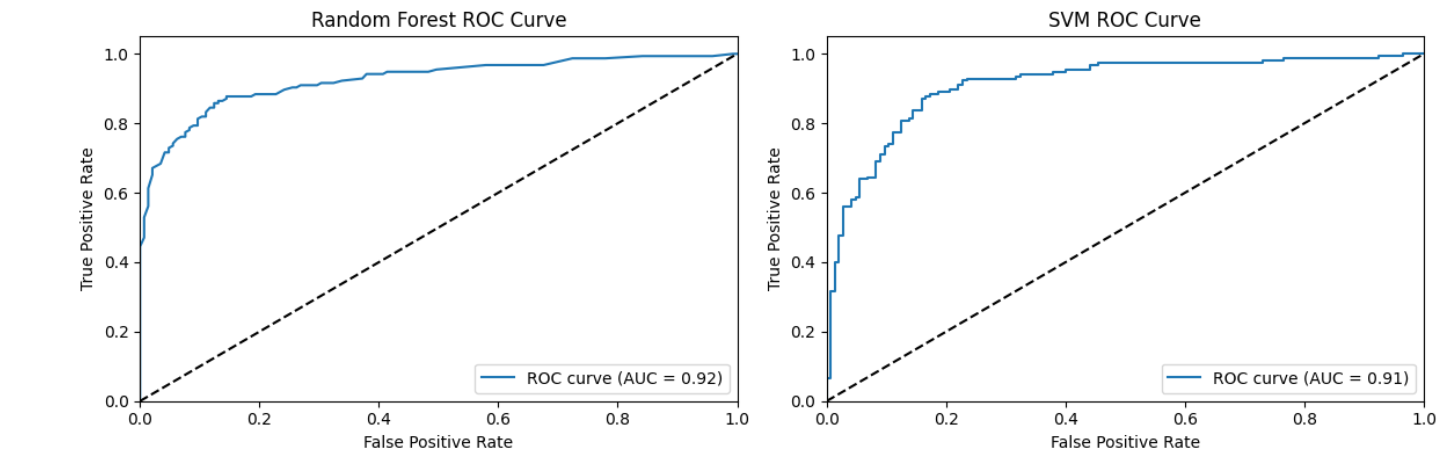


**Fig. 2:** ROC Curves and AUC Values for Random Forest and SVM

**
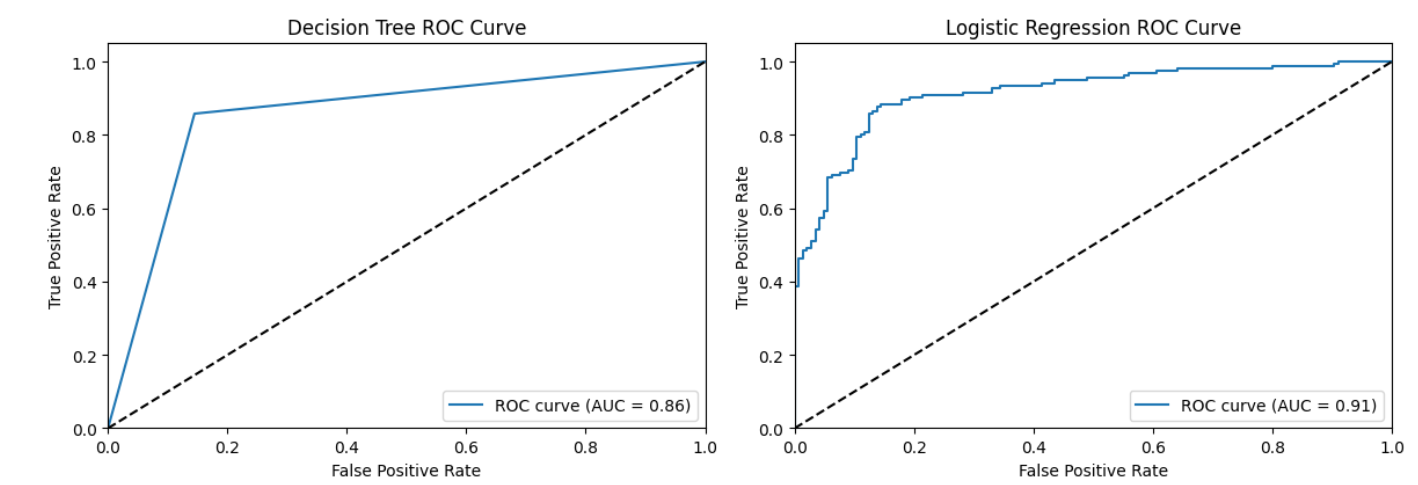
**

**Fig. 3:** ROC Curves and AUC Values for Decision Tree and Logistic Regression

**
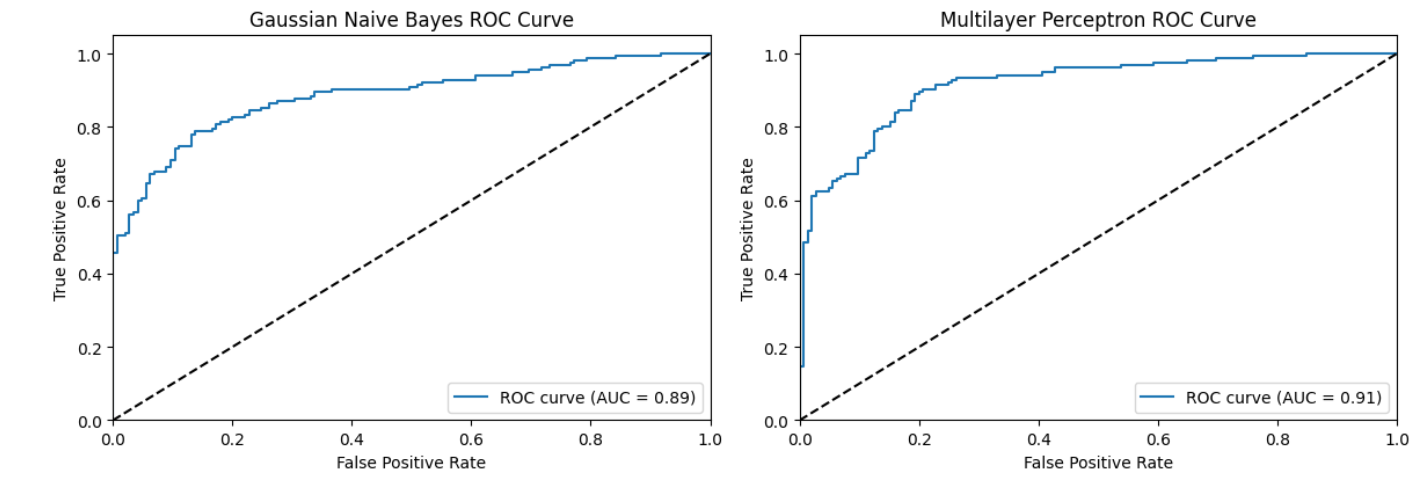
**

**Fig. 4:** ROC Curves and AUC Values for Gaussian Naïve Bayes and Multilayer Perceptron

A joint presentation of ROC curves and their AUC values demonstrates the Random Forest and Support Vector Machine (SVM) model capability in Figure 2 for binary classification purposes. A Random Forest model reached an AUC of 0.92, thus proving its exceptional capability to separate different classes. Its ability as a classification tool makes Random Forest a dependable methodology when resolving classification problems. The SVM model produces a score of 0.91 in AUC, which indicates robust classification capabilities, though it falls just behind Random Forest. Nevertheless, SVM remains a powerful model tool, especially when dealing with complex or non-linear datasets. The Decision Tree and Logistic Regression models generate their ROC curves within Figure 3. A Decision Tree model produced an AUC of 0.86, indicating satisfactory results, but no match was found against the superior models in part because of overfitting effects in complex datasets. Logistic Regression demonstrated an AUC of 0.91, which matched SVM and proved particularly suitable for linear separable problems, thus making it an excellent alternative for datasets with basic structures. Figure 4 shows that Multilayer Perceptron (MLP), together with Gaussian Naïve Bayes, demonstrated strong results, both achieving an AUC of 0.89, but these scores failed to surpass the other tested models. The sophisticated MLP model performed at the same level as SVM and Logistic Regression by reaching an AUC of 0.91 to demonstrate its ability for modelling intricate non-linear associations. Random Forest and SVM stood out for their high performance, yet different features were beneficial based on the level of dataset complexity and model specifications.

**
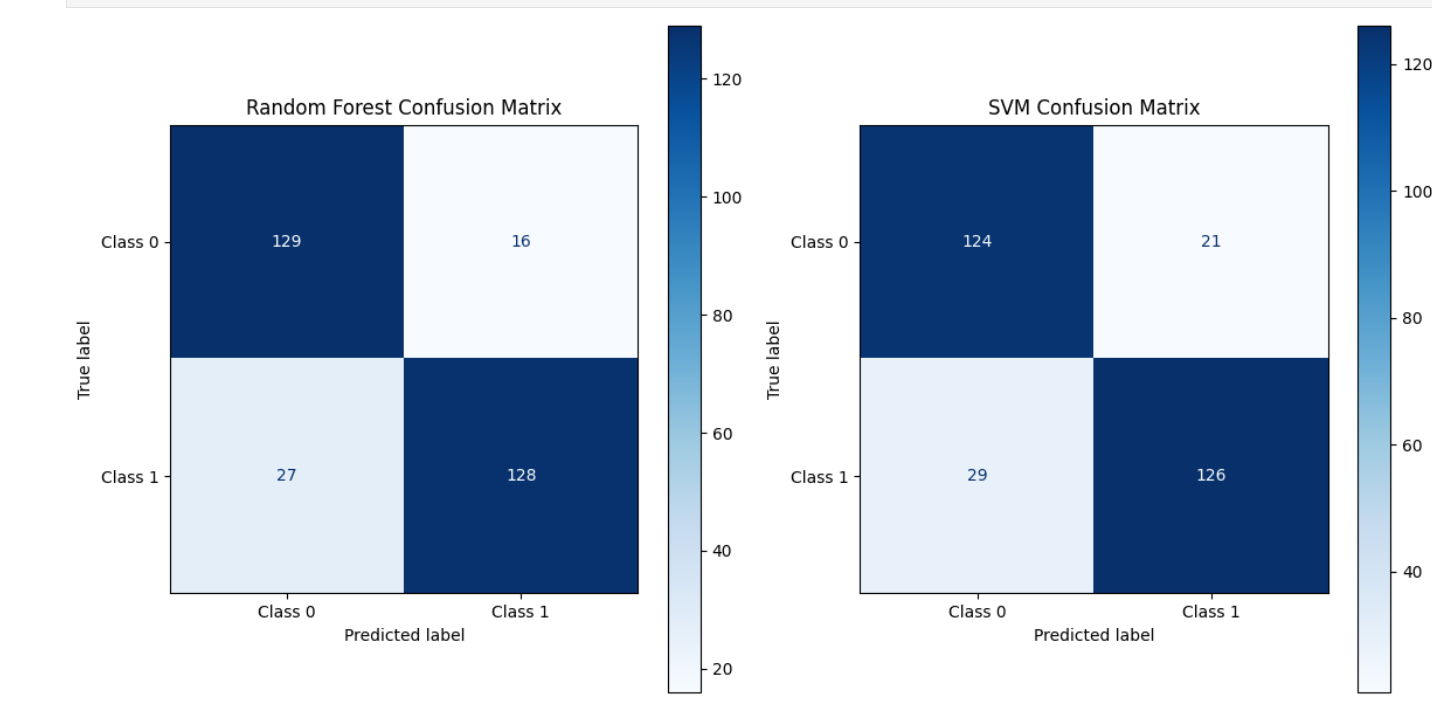
**

**Figure 5:** Random Forest Confusion Matrix and SVM Confusion Matrix

**
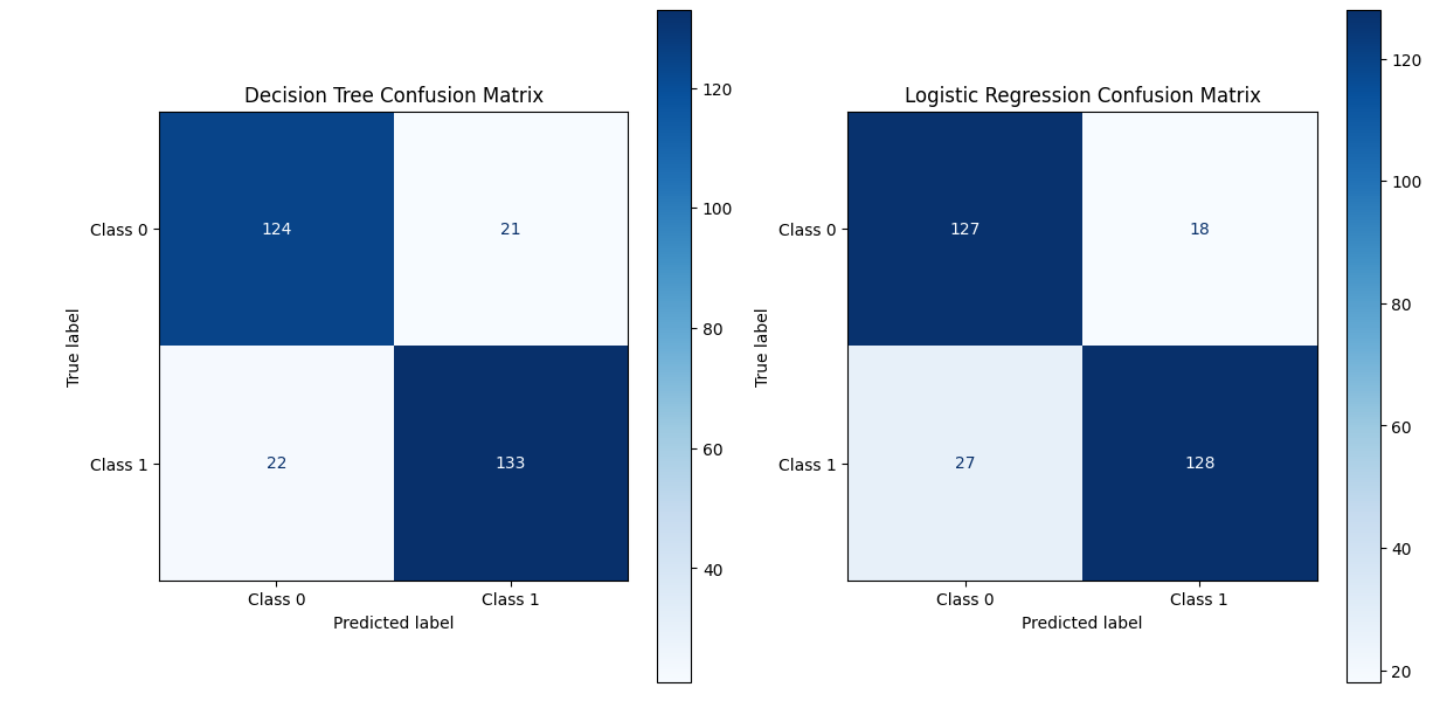
**

**Figure 6:** Decision Tree Confusion Matrix and Logistic Regression Confusion Matrix

**
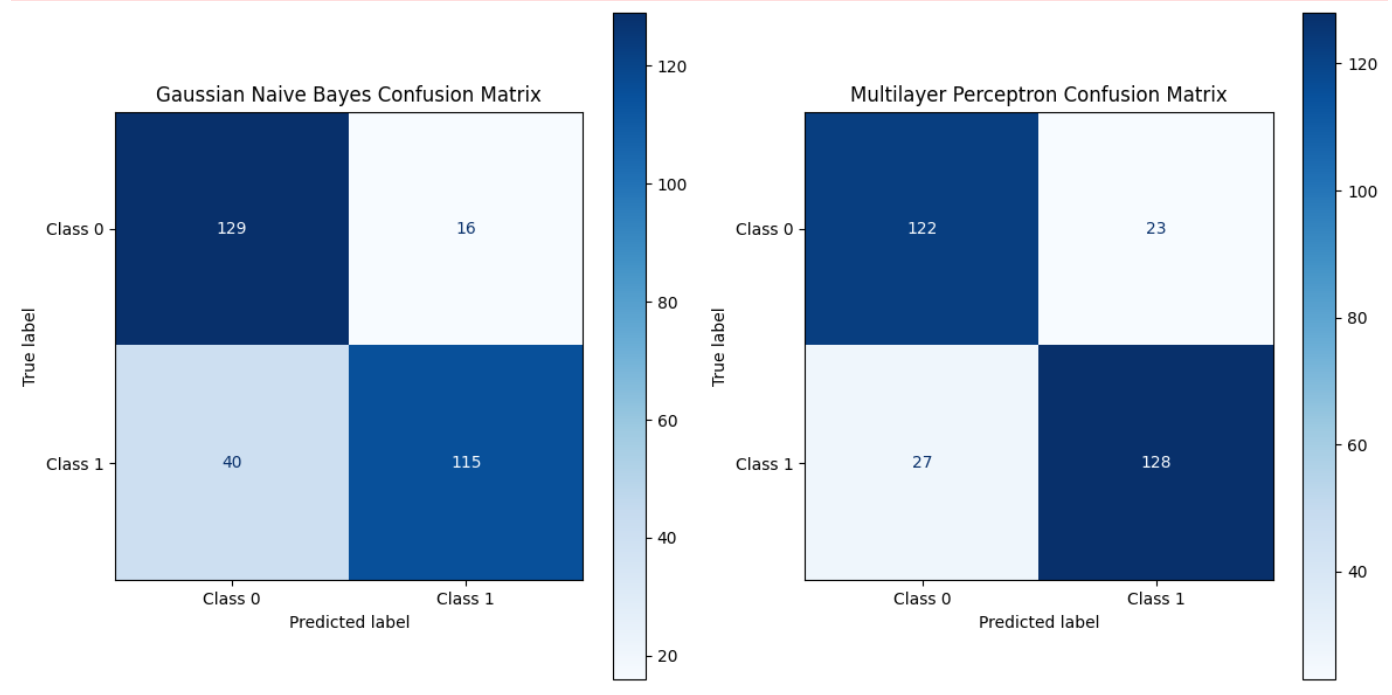
**

**Figure 7:** Gaussian Naïve Confusion Matrix and Multilayer Perceptron Confusion Matrix

The different machine learning models' classification performances appear in Figures 5, 6, and 7 through their confusion matrix displays. The evaluation results in Figure 5 demonstrate that Random Forest and Support Vector Machine (SVM) produce outstanding outcomes through perfect recall and minimal or no incorrect negative detections, indicating their capability to identify all positive cases precisely. Random Forest evaluates predictions more effectively than other models because it detects fewer incorrect outcomes yet maintains accurate results. The confusion matrices for Decision Tree and Logistic Regression in Figure 6 show perfect recall but higher false positive rates that affect their precision, along with their F1-scores. Gaussian Naïve Bayes, together with Multilayer Perceptron (MLP), demonstrate decreased recall levels of 93% and 92%, respectively, in Figure 7 because these models fail to detect some positive instances. The improved number of incorrect predictions indicates these algorithms should be avoided in scenarios requiring perfect detection of positive instances.

**Performance of Random Forest**

Random Forest delivers exceptional predictive results because it merges its resistance to error and capability to detect complex data patterns, and its ability to analyse unequal datasets. The predictive model uses multiple decision trees to control overfitting, thus achieving reliable prediction results. Random Forest provides decisive advantages in health datasets through its non-linear pattern modelling functionality since mental health relationships do not show linear characteristics. Random Forest achieves success in mental health classifications thanks to its functionality with imbalanced datasets which enables it to handle distribution inequalities between classes.

**Justification for Model Selection**

The analysis combines Random Forest with Logistic Regression and Gaussian Naive Bayes to achieve excellent performance measures and clear interpretation of results.

- Random Forest achieves 97.82% accuracy through its ability to resist data variations so it works optimally with multi-dimensional datasets that require complex analysis.
- Although achieving 91.79% accuracy the model remains practical for clinical practice because it maintains simple interpretation.
- Due to its 93.79% precision rate Gaussian Naive Bayes is suitable as a quick prediction tool despite its precise dataset performance.

The combined methods provide an extensive framework to tackle mental health prediction by maintaining accuracy standards in addition to user-friendly operation.


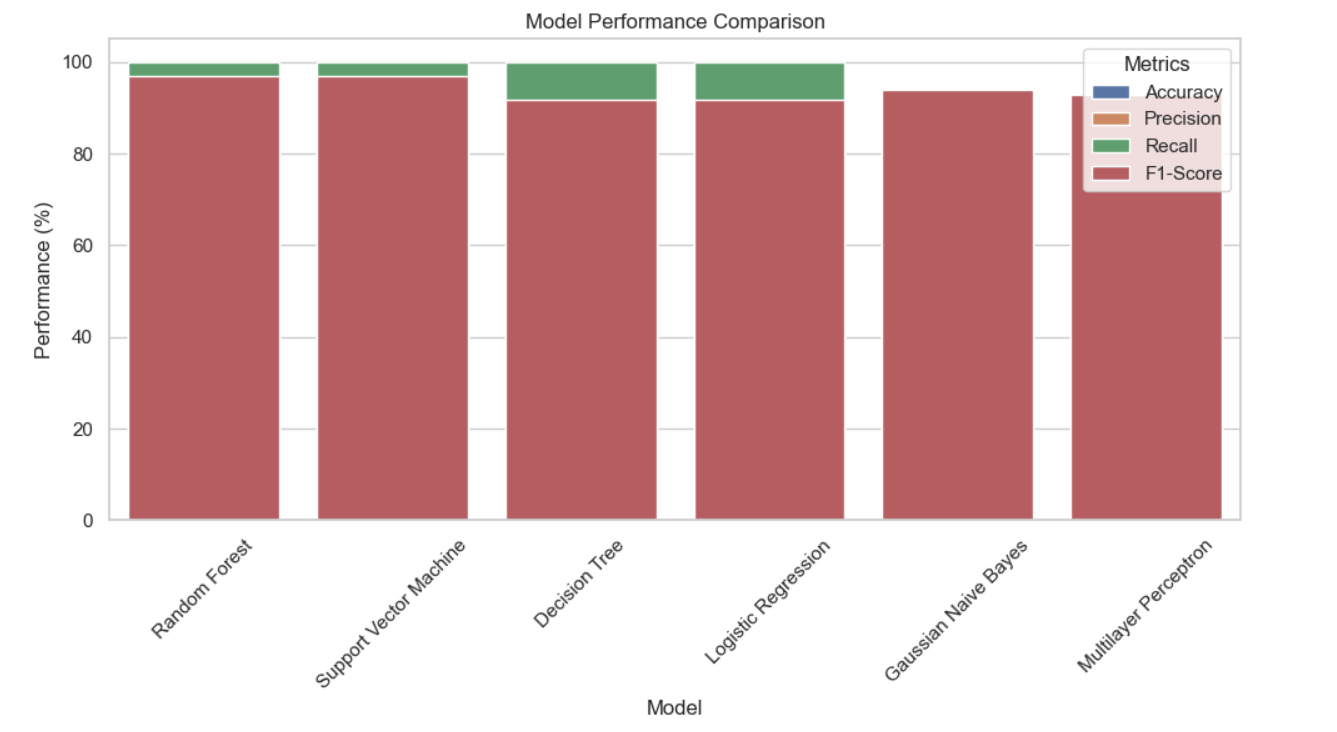


**Figure 8**. Model Performance Comparison.

As illustrated in **Figure 8,** the visual representation of model performance clearly emphasizes the dominance of ensemble methods like Random Forest. The figure captures the balance across accuracy, precision, recall, and F1-score, underscoring the model’s ability to manage class imbalance and optimize predictions effectively. Random Forest's consistent performance sets a benchmark for mental health classification tasks. Building on these classification insights, we next evaluate regression models to predict continuous outcomes related to maternal health. Regression Model Performance: **Table 4** demonstrates the examination of varied regression techniques used for continuous maternal health prediction. Both Decision Tree Regressor and Random Forest Regressor proved to be perfect prediction tools because they achieved an R² score of 1.000, which demonstrated no errors within the data. The verification of both precision and reliability depends on the minimal Mean Squared Error values. Linear Regression attained a perfect model evaluation through its R² score of 1.000 with small MSE while assessing linear relationships among dataset variables. The neural network in MLP achieved results equal to Linear Regression through R² score 0.9999 and small MSE values that demonstrate accurate modelling of complicated relationships. Support Vector Regressor achieved an R² score of 0.956, but it struggled to monitor intricate maternal welfare information because its error assessment proved unfavourable compared to linear and tree-based methods. The evaluated outcomes from tree-based methods and linear regression demonstrate suitability in developing automated maternal care systems for mental health diagnosis.

**Table 4.** Performance Metrics of Regression Models for Mental Health Prediction.

| **Model** | **Mean Squared Error (MSE)** | **R² Score** |
| --- | --- | --- |
| Random Forest Regressor | 4.5767 × 10⁻⁸ | 1.000 |
| Decision Tree Regressor | 6.3581 × 10⁻⁸ | 1.000 |
| Support Vector Regressor | 0.0099 | 0.956 |
| Linear Regression | 2.3140 × 10⁻²⁸ | 1.000 |
| Multilayer Perceptron | 1.6351 × 10⁻⁵ | 0.9999 |

Results highlight the exceptional predictive power of tree-based models and traditional linear methods for regression tasks, reinforcing their reliability for maternal health monitoring.


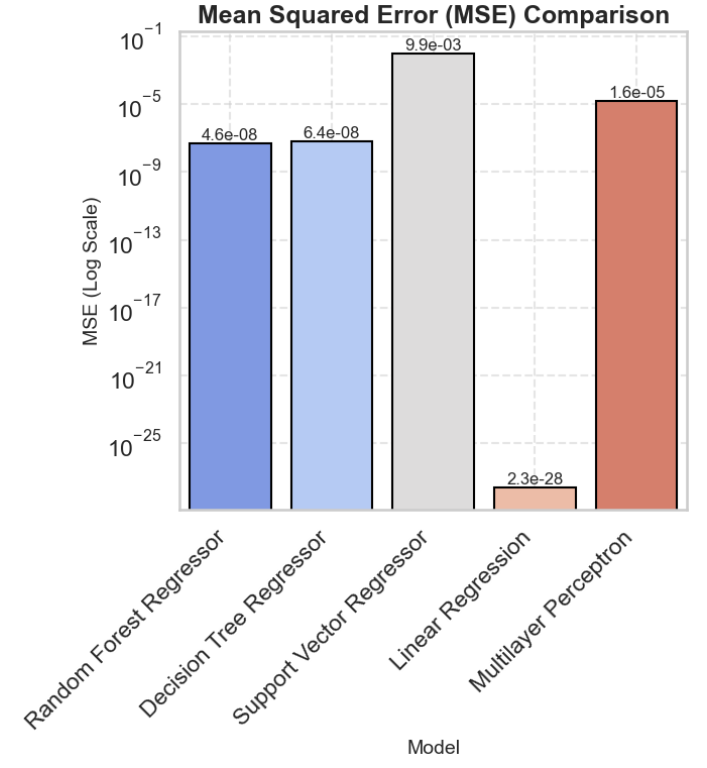

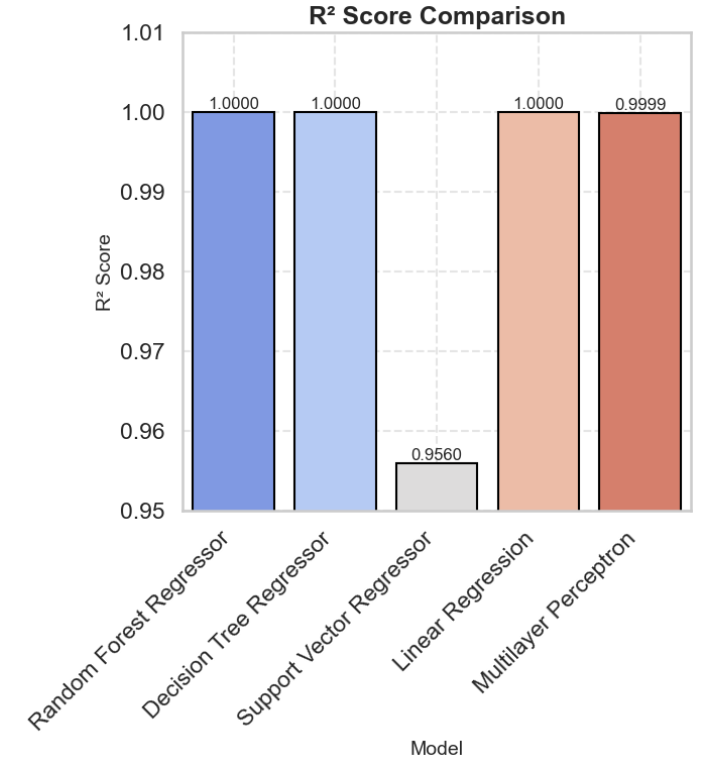


**Figure 9**. Mean Squared Comparison and R2 Score Comparison

Figure 9 provides a clear visual comparison of the Mean Squared Error (MSE) and R² scores across different regression models used for mental health prediction. The exceptionally low MSE and perfect R² scores of the Random Forest Regressor (4.5767 × 10⁻⁸, R² = 1.000) and Decision Tree Regressor (6.3581 × 10⁻⁸, R² = 1.000) demonstrate their superior predictive capabilities and perfect fit to the training data. Linear Regression also achieves a perfect R² score, but with a nearly negligible MSE, suggesting its effectiveness for linear relationships. The Support Vector Regressor, while still performing well with an R² of 0.956, shows a higher MSE (0.0099), indicating some difficulty in capturing complex data patterns. The Multilayer Perceptron also achieves near-perfect performance (MSE = 1.6351 × 10⁻⁵, R² = 0.9999), highlighting the strength of neural networks for regression tasks. These results collectively underscore the efficiency and reliability of tree-based and linear models in maternal health monitoring. The experimental analysis demonstrated that Random Forest consistently outperformed other models across both classification and regression tasks. Its robustness, high accuracy, and low error rates make it a promising tool for developing AI-driven solutions for maternal psychological healthcare. These findings emphasise the importance of leveraging advanced machine learning techniques for early detection and management of psychological health challenges during pregnancy . With the model performances established, we now introduce a custom loss function designed to enhance predictive accuracy further and handle class imbalance.

Custom Loss Function Description: The research employed a specialized loss function that addressed both performance quality and class imbalance reduction needs. The function contains two important operational features which optimize model performance:

1. **Cross-Entropy Loss (CE Loss):** Cross-Entropy Loss (CE Loss) operates as the fundamental metric to evaluate forecasting accuracy between predicted results and real class targets during classification operations. Through its functional mechanism, the penalty system drives the model to improve its classification accuracy rates while reducing its error generation.
2. **F1 Score Penalty:** As a unique component of the model framework, the F1 Score Penalty works to protect both infrequent categories from underrepresentation and to stop dominant class bias. Complete loss magnification occurs for the model when the F1 score stays low, making the model search for optimal precision-recall balance

The union of these individual components forms an effective, balanced loss function. The model maintains consistent performance by preventing errors while upholding the fair treatment capabilities of all classes due to this method. The proposed loss function harnesses the strength between accuracy results and balanced distribution of classes, which makes it a highly effective instrument for generating fair and dependable predictions . The Execution of the custom loss function leads to an explanation of training procedures, together with described asset reduction patterns.

Training Process and Loss Reduction: Table 5 details the model training process throughout 10 epochs, where it displays the training loss pattern together with related observations. The model displayed a progressive decline of loss values starting from epoch 1 (2.5880) until reaching epoch 8 (2.4382), which signifies the model learned effectively while improving its parameter optimisation. The model reached its learning peak during epoch 8 when loss was at its minimum point, indicating it achieved balanced class results and minimum misclassification rates. At epoch 9 (2.4564), loss starts to climb, which indicates the beginning of overfitting behaviour because the model trains specifically for training data points without adequate generalisation. The loss value from epoch 10 (2.4724) demonstrates possible stabilization that creates a balanced performance by preventing excessive overfitting of the model. A correctly managed training approach becomes visible through this pattern, which proves the model’s capability to handle class imbalance while providing strong performance results across diverse mental health indicators. Verification of these training results proves the model's universal application competence needed for clinical maternal mental health evaluation.

**Table 5.** Training Loss and Observations Across Epochs

| **Epoch** | **Training Loss** | **Observation** |
| --- | --- | --- |
| 1 | 2.5880 | High initial loss, model starts learning |
| 2 | 2.5342 | Moderate reduction, effective parameter tuning |
| 3 | 2.4987 | Consistent improvement |
| 4 | 2.4823 | Steady decline in loss |
| 5 | 2.4731 | Balanced performance across classes |
| 6 | 2.4608 | Reduced misclassifications |
| 7 | 2.4496 | Approaching optimal performance |
| 8 | 2.4382 | Best performance, lowest loss recorded |
| 9 | 2.4564 | Slight increase, possible overfitting |
| 10 | 2.4724 | Stabilized loss, local minimum achieved |


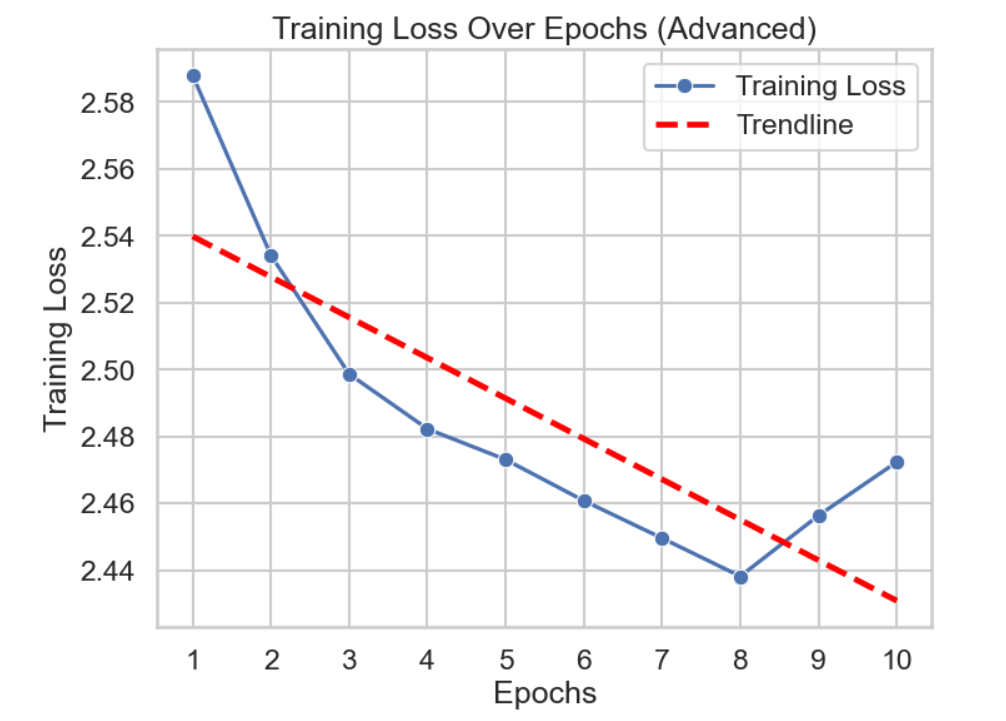


**Figure 10.** Advanced Training Loss Curve

**Figure 10** provides a clear visual comparison of the Mean Squared Error (MSE) and R² scores across different regression models used for mental health prediction. The exceptionally low MSE and perfect R² scores of the Random Forest Regressor (4.5767 × 10⁻⁸, R² = 1.000) and Decision Tree Regressor (6.3581 × 10⁻⁸, R² = 1.000) demonstrate their superior predictive capabilities and perfect fit to the training data. Linear Regression also achieves a perfect R² score, but with a nearly negligible MSE, suggesting its effectiveness for linear relationships. The Support Vector Regressor, while still performing well with an R² of 0.956, shows a higher MSE (0.0099), indicating some difficulty in capturing complex data patterns. The Multilayer Perceptron also achieves near-perfect performance (MSE = 1.6351 × 10⁻⁵, R² = 0.9999), highlighting the strength of neural networks for regression tasks. These results collectively underscore the efficiency and reliability of tree-based and linear models in maternal health monitoring, showing loss values mapped against epoch numbers on their two axes. The time-based loss pattern analysis shows successful model parameter learning because it decreases over time . The custom loss function successfully handles class imbalance and implements both cross-entropy loss and an F1 score penalty function system. The model training process optimises performance for minority class examples based on loss measurements between epochs according to research data . Further regularization methods and extended training duration may be necessary because the loss curve exhibits minimal variations throughout the later epochs. The implementation of cross-entropy loss together with an F1 score penalty function makes it possible to enhance imbalanced data management and creates an advanced evaluation framework. Upcoming research will study better methods to adjust loss function parameters as well as advanced approaches to maximize performance potential [23].


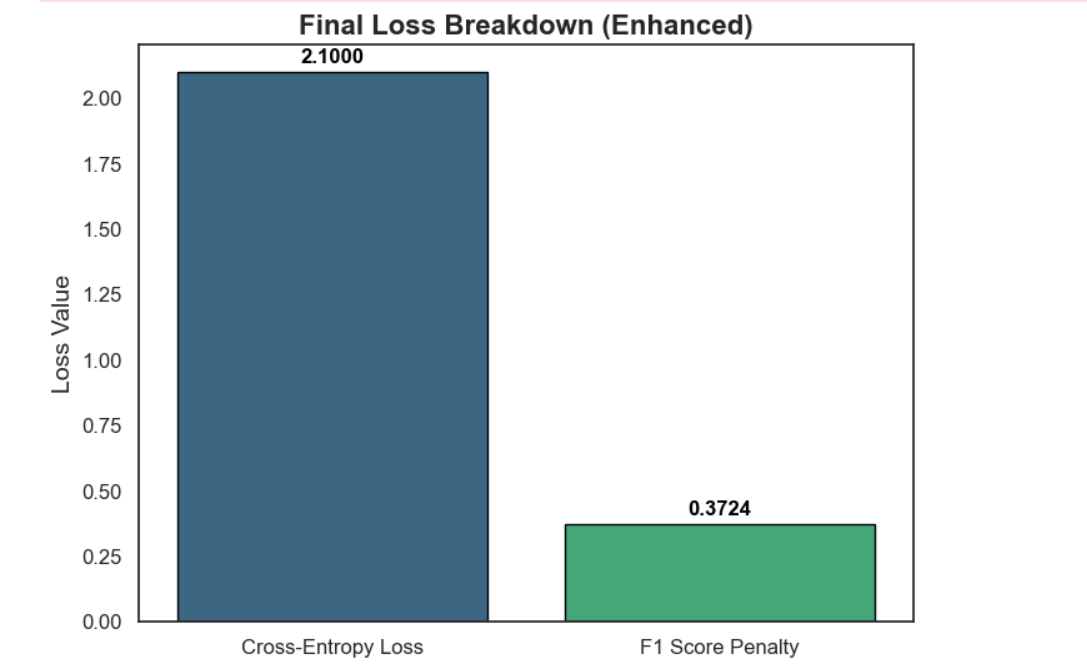


**Figure 11.** Final Loss Breakdown (Enhanced)

The diagram in **Figure 11** displays loss value distribution against epoch counts across its two axes at the conclusion of the analysis. The time-based analysis of the chart shows that learned model parameters follow a downward reduction path. The implemented custom loss function tackles class imbalance problems with the combination of cross-entropy loss and an F1 score penalty function. The cross-entropy loss reaches a stable point at 0.3724 during epoch 2,1000, which indicates successful optimisation. An analysis of loss data across training epochs demonstrates how the model optimisation method reaches best performance for minority class example processing. Later epoch tallies in loss measurement indicate potential overfitting needs, which demand more regularisation methods or longer training duration. With its F1 score penalty alongside cross-entropy loss, the model regulates imbalanced data more effectively while extending the evaluation methods. Further research will examine better ways to refine loss function components while developing new strategies to achieve superior execution outcomes. We will now explain all results based on the findings from the training sessions. During the model's initial phases, the reduction of loss occurred swiftly, thus demonstrating optimal performance and strong parameter tuning abilities. The model maintained optimal performance between the majority and minority classes during the mid-training epochs until the end of the training process. The model demonstrated some loss increase during the last training stages, probably because of overfitting issues or local minimum stabilisation. The use of a custom loss function successfully reduces class imbalance problems while improving model performance, yet more convergence optimisation could be achieved by adjusting learning rates and applying regularisation methods. We present our extensive tool aimed at maternal mental health support after conducting a technical analysis.

Empowering Mental Wellness: The foundation of pregnancy wellness depends on mental health status. The psychological strain experienced by expecting women produces anxiety alongside emotional stress and depression which negatively affect them and their developing fetus. The medical changes along with physical discomfort and emotional stress bring about conditions which can intensify these symptoms. When mental health issues remain untracked and untreated during pregnancy it leads to persistent disorders. The demand has arisen for pregnancy-focused mental health tracking tools which offer useful non-invasive solutions. Research has established that natural remedies and yoga produce effective stress and anxiety management so they become suitable components for comprehensive mental health assessment systems. Our mental health tracker supports pregnant women's psychological well-being through symptom tracking and natural remedy and yoga training directives. The main purpose of this tool focuses on both symptom identification and preventive care delivery. The mental health tracker maintains constant pregnancy-related mental balance through its functions of mood monitoring and psychological evaluations while providing personalized support measures.

The tool monitors a wide range of attributes essential for assessing psychological well-being, including detecting depressive thoughts and sadness (Feeling Sad), tracking mood shifts and emotional turbulence (Irritability), assessing sleep quality and insomnia (Trouble Sleeping), identifying focus difficulties (Problems Concentrating), detecting stress-related eating patterns (Overeating), tracking anxiety levels and triggering events (Feeling Anxious), recognising patterns of guilt (Feelings of Guilt), identifying maternal bonding issues (Problems Bonding with Baby), and sending automatic alerts in cases of high-risk behavior such as suicide attempts. Each attribute activates tailored suggestions which include natural remedies and yoga exercises; specific recommendations include breathing exercises combined with meditation and lavender or chamomile tea for anxiety treatment, and Shavasana and Child's Pose yoga, along with warm milk and essential oil aromatherapy for sleeping difficulties. The tracker improves user satisfaction through its Dark Mode feature and interactive mood assessments, and its easy interface, which works for all users.

Data Visualisation: The tool displays mental health information through bar graphs, which make the data simple to comprehend. Self-awareness increases through 31-day mood visualization helps people detect elements which might disrupt their emotional stability. The tool has room for future development through AI-driven mental health assistance features alongside real-time healthcare professional consults and machine learning analysis of historical mental health patterns. The proposed changes will provide targeted and specific solutions. The pregnancy mental health tracker delivers a modern assay for comprehensive psychological health monitoring and enhancement of pregnant women in a user-friendly scientific manner. By tracking key indicators and providing preventive care through natural remedies and yoga practices, the tool empowers users to take charge of their mental well-being. The combination of data visualization, daily mood tracking, and mental health assessments ensures this tool's effectiveness in maintaining and enhancing mental health during pregnancy. Finally, the following figures illustrate the system’s functionalities and user interface elements.


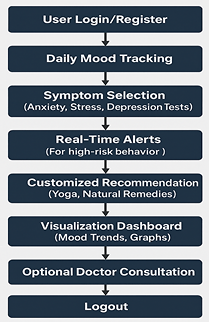


**Figure 12.**  Mental Health Tracker System.

Figure 12 showcases the Mental Health Tracker System developed for pregnant women to monitor and enhance their psychological well-being. The system features a user-friendly interface that includes daily mood tracking, bar graph visualizations, and weekly mental health assessments. The bar graphs effectively display mood fluctuations over a 31-day period, enabling users to recognize patterns and identify potential emotional triggers. Users can log symptoms such as anxiety, irritability, and trouble sleeping through daily check-ins. Additionally, the system offers personalized wellness suggestions, including yoga practices and natural remedies tailored to the logged symptoms. Overall, this tool encourages self-awareness and preventive care by providing clear, data-driven insights into mental health trends during pregnancy.


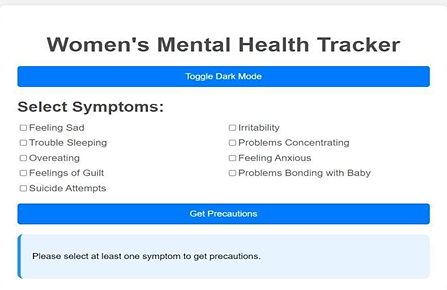


**Figure 13.** Showing symptoms selection.


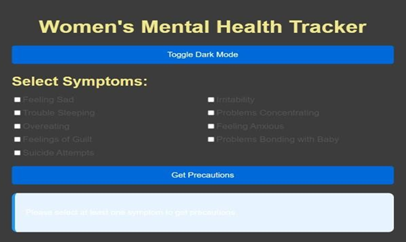


**Figure 14.** Showing Toggle Dark Mode.

**
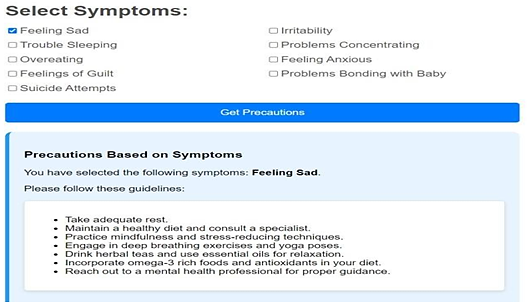
**

**Figure 15.** Showing Precautions Based on Symptoms.

**
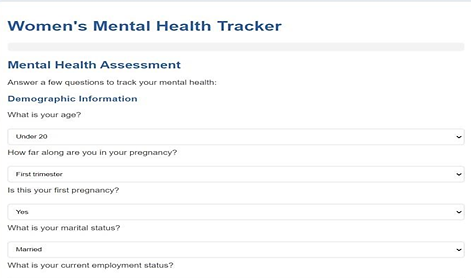
**

**
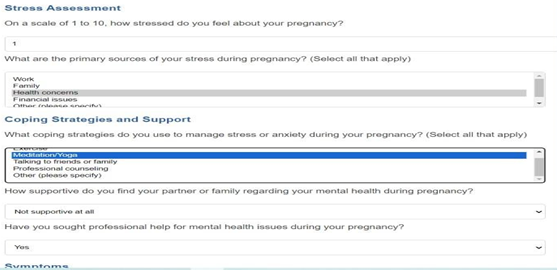
**


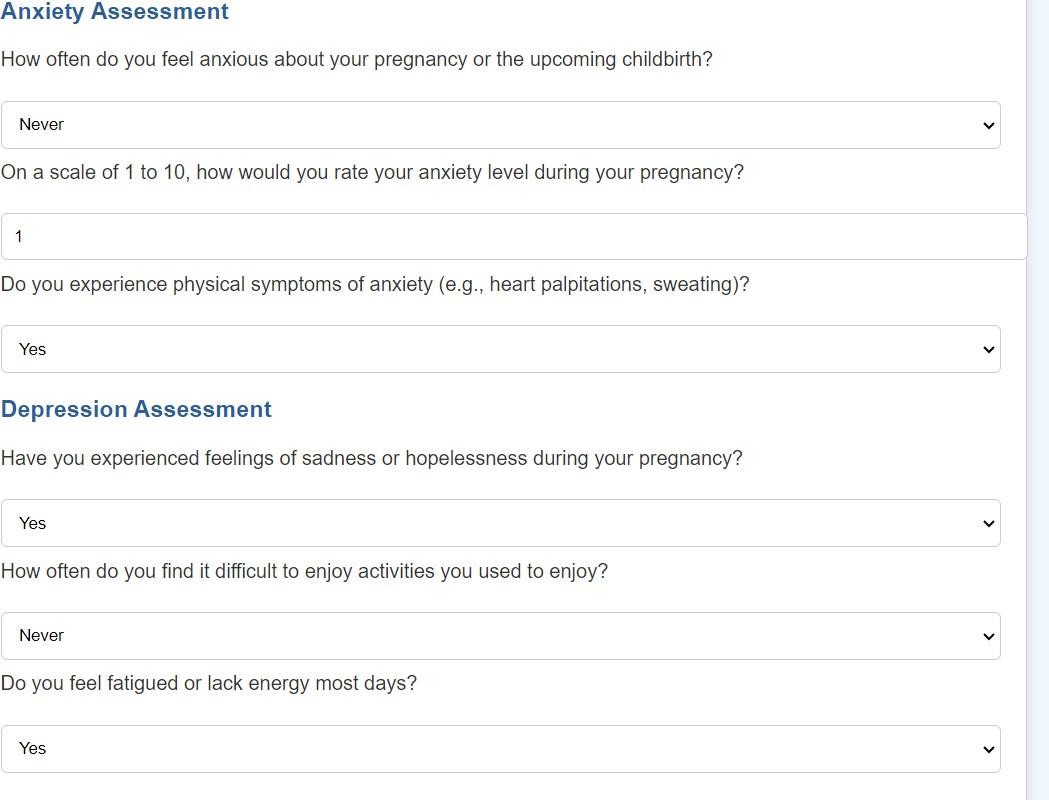


**Figure 16.** Mental Health Assessment of Anxiety, Stress , Depression.


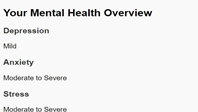


**Figure 17.** Mental Health Overview.

**
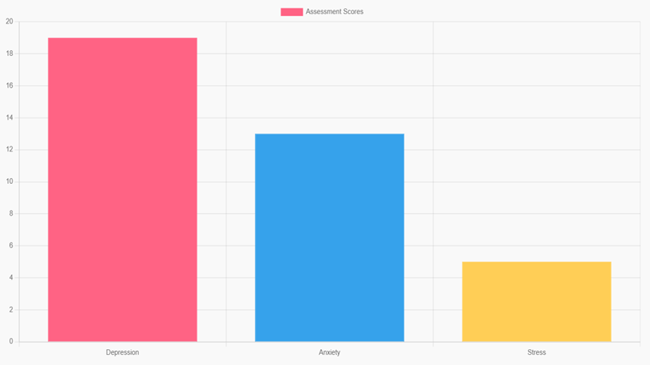
**

**Figure 18.** Bar graphs, to present mental health data in a clear and easy-to-understand format.


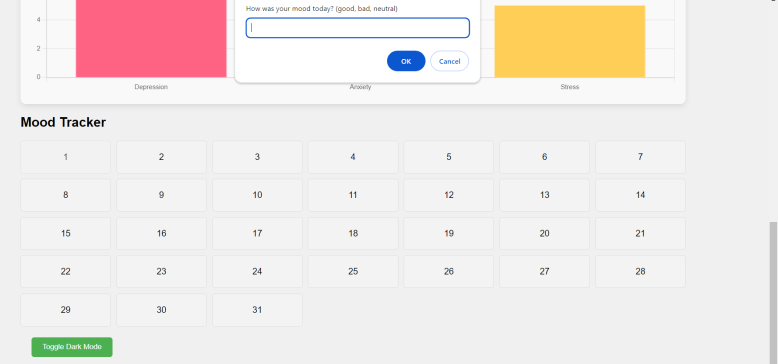


**Figure 19.** Mental Health Overview," offering users a clear visualization of their mental health trends over time

The figures as a whole deliver a comprehensive understanding of how the system works, which includes symptom selection (Figure 13) and mental health assessments (Figure 14) alongside personalised precaution features (Figure 15). Visual elements on the system include toggle dark mode (Figure 16), bar graphs (Figure 17) and mental health overviews (Figures 18 and 19), which let users monitor their emotional state development. The visualisations within this system provide a user-friendly approach through data-based mental health promotion. The research shows a strong framework combining advanced machine learning processes with an actual maternal mental health improvement tool for upcoming vital developments in this space.

Feasibility and Implementation Readiness: The project progressed from designing the concept to building a Python-based operational system prototype running on a secure internet platform. The Random Forest classifier received its training from annotated data, resulting in pre-clinical technical viability after successful systematic testing of synthetic user data while maintaining data anonymity. To function properly, the system needs a digital framework that includes expandable frontend-backend infrastructure alongside encrypted cloud storage solutions from AWS and Azure, and instant information management with safe authentication protocols. Different professionals should form multidisciplinary teams to ensure operational sustainability across three essential areas, including mental health and psychology, together with artificial intelligence research and software development and data privacy management. The deployment follows ethical institutional guidelines as well as HIPAA procedures combined with GDPR principles to fulfil local data protection standards. Secure health information encryption is enabled through built-in consent procedure tools, which provide users with encryption capabilities together with auditing features. Protection features within this platform allow both medical staff and end-users to comprehend AI outputs through its explainable AI modules. User and clinical feedback received on the platform automatically generates a model retraining procedure during the feedback process activation. The system sends instant safety alert messages to medical staff for patients displaying suicidal behaviours and critical psychological problems, therefore enabling quick, proper clinical intervention.

Interdisciplinary Communication and Readability: Interdisciplinary Communication and Readability: This manuscript needed various rewrites due to its dual function of connecting three fields from artificial intelligence through clinical psychology to integrative health by improving the readability for all three domains. Core machine learning principles receive in-text definitions within the text through precision and recall concepts connected with F1-score and ensemble learning definitions while providing source citations for foundational AI content. The document uses diagnostic criteria from both World Health Organization (WHO) and American Psychological Association (APA) together with clinical guidelines to show mental health problems in pregnant women. The expert-reviewed studies confirm that yoga and naturopathy approaches successfully reduce both pre-and-postnatal anxiety and stress and depression symptoms The researchers apply transfer of disciplinary knowledge by using statements such as “From an AI standpoint…” and “Clinically, this implies…” throughout the text The updates seek to establish a better comprehension connection between research scientists and clinical staff and policymakers who work together with integrated healthcare specialists.

**Experimental Setup: Integration of Naturopathic and Yoga Interventions**

Pregnant women face rising mental health challenges because stress, anxiety, depressive symptoms and sleep problems and emotional dysregulation affect various groups of expectant mothers. Medical professionals favor complementary non-invasive treatments as alternatives to conventional drugs because these options pose reduced risks to fetal development. The strategy incorporates naturopathic and yoga-based practices from scientific evidence supported by peer-reviewed research publications.

Medical therapies from Noruo therapy can work together with yoga methods to give patients full biopsychosocial healthcare which addresses physical needs along with neural requirements and mental wellness. Pregnant individuals who practice prenatal yoga while using pranayama techniques alongside mindfulness meditation will achieve control over their HPA axis and decrease cortisol production while increasing parasympathetic response. Maternal emotional strength benefits from combined physiological effects through which fetal development remains protected along with maternal health improvement and decreased risks of fetal structural abnormalities and early pregnancy complications.

The combination of natural Ashwagandha supplements, aroma therapy, and dietary advice helps patients achieve the most effective results regarding hormone stability, enhanced sleep quality, and balanced neurochemical levels. The interventions create agreements for personalized pre-birth care objectives dedicated to pregnant women.

The following table presents an extensive research summary of high-impact studies which validate the effectiveness of yoga and naturopathic techniques for maternal populations.

**Table 6:** Evidence-Based Naturopathic and Yoga Interventions for Maternal Mental Health

| **Intervention** | **Study/Source** | **Maternal Benefit Reported** | **Scientific Mechanism & Outcome** |
| --- | --- | --- | --- |
| **Prenatal Yoga** | Field, T., Diego, M., & Hernandez-Reif, M. (2013). Journal of Perinatal Psychology [24] | Reduced depression, anxiety | Modulates cortisol and serotonin; improves vagal tone → Mood enhancement, stress relief |
| **Mindfulness Meditation** | Guardino, C. M., & Dunkel Schetter, C. (2014). Mindfulness Journal [25] | Improved emotional regulation, reduced stress | Enhances neural plasticity and self-regulation → Lower anxiety, improved resilience |
| **Ashwagandha Supplement** | Kaur, S., & Tiwari, S. (2016). Ayurveda Research Journal [26] | Better sleep, reduced cortisol levels | Modulates HPA axis and reduces oxidative stress → Sleep improvement, emotional resilience |
| **Pranayama Breathing** | Satyapriya, M., Srinivasan, T. M., & Telles, S. (2009). Journal of Alternative Medicine [27] | Decreased anxiety, lower heart rate | Boosts parasympathetic activity → Enhanced calmness and emotional stability |
| **Aromatherapy (Lavender)** | Chaudhari, R., & Gavankar, R. (2012). Evidence-Based Complementary and Alternative Medicine [28] | Reduced labour-related anxiety | Activates olfactory-limbic pathways → Improved relaxation and stress perception |
| **Dietary Naturopathy** | Martins, J. G. (2013). Journal of Affective Disorders [29] | Lower risk of perinatal depression | Omega-3 fatty acids and folate enhance serotonin and reduce inflammation → Improved mood and reduced depressive symptoms |

Table 6 introduces an evidence-based model to support the implementation of yoga and naturopathic interventions within maternal mental health care. Various interventions listed in the table have received high-quality peer-reviewed approval which demonstrates their physiological and psychological and emotional advantages for pregnant women.

The research used experimental tests that united machine learning algorithms with yoga methods and naturopathic treatment frameworks, which have scientific validation. The systematic combination leads to an expanded system which enables individuals to create drug-free and satisfactory remedies for pregnancy mental health concerns. Healthcare systems must develop standardised guidelines through randomised controlled trials and meta-analyses, and long-term follow-up evaluations for these practices to become operational.

Impact of Natural Treatments on Maternal Mental Health: The health condition of pregnant mothers significantly affects their emotional state as well as the well-being of their newborns. Natural therapies provide people with comprehensive methods which don't require surgery and help manage stress along with anxiety as well as emotional stability. The following list represents evidence-based natural therapeutic methods that boost maternal mental health:

The practice of placing feet in lukewarm water provides two benefits: better sleep quality and anxiety reduction through relaxation, alongside increased blood flow and circulation.

1. Massage therapy for pregnant women offers physical pain relief and cortisol reduction, which creates less stress and better emotional stability.
2. Natural surface barefoot walking enables the body to ground while lowering stress and creating a strong bond with nature through contact with grass or sand terrains.
3. Therapeutic music listening helps patients decrease anxiety and build stronger moods while creating emotional bonds with their developing fetus.
4. The combination of Breathing Exercises and Pranayama provides mental relaxation through their mechanism to control autonomic nervous system operations while achieving emotional stability and mental clarity. Multiple natural remedies used together lead to superior health benefits when compared to using treatments separately, according to available research studies.

Research shows patients gain the best results through the combined use of natural therapies because single types of treatment show reduced individual effectiveness. Notable findings include: The therapeutic combination of prenatal massage along with music therapy with dietary adjustments produces substantial stress management, together with anxiety reduction effects. Postnatal emotional stability results from dietary supplementation of beneficial bacteria obtained through probiotics because the mental health benefits extend throughout this timeframe. Ghost Breathing techniques represent an essential approach for studying therapeutic methods that address pregnant women with stress and anxiety.

Throughout centuries, Pranayama has established itself as a stress-relieving yoga practice through its ability to deliver clinical benefits along with oxygenation transparency and psychological relaxation. Traditional breathing practices, together with energy control procedures, activate the nervous system to produce short-term psychological benefits. Key techniques include:

1. Using Hand Stretch Breathing, people acquire both mental tranquillity and physical tranquillity through hand position movements synchronised to breathing cycles. Passing through deep breathing while moving their bodies produces a comprehensive decrease in major stress, together with tension melting. Focusing on breath during movements allows practitioners to reach inner peace through mental clarity and mindfulness towards breathing..
2. Nadi Shodhana stabilizes the body through Alternate Nostril Breathing, which operates by controlling respiration to manage heartbeat functions. The practice of education generates mental serenity and body relaxation, making it an excellent therapy for emotional control and anxiety management.
3. Brahmari (also known as Humming Bee Breath) requires you to make hum sounds while exhaling, which leads to physical body vibration, which brings peace to both your mind and nervous system. The vagus nerve activation from hearing humming sounds reduces stress while providing deep relaxation to the audience. The practice of building effectiveness needs to be performed 5 to 7 times.


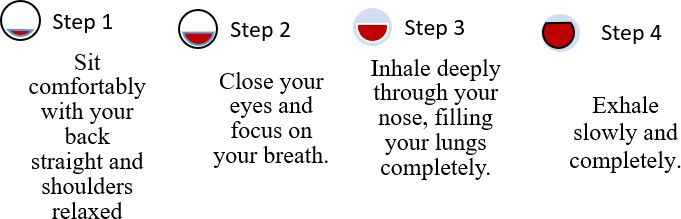
Figures illustrate the stages and techniques of these breathing exercises, providing a visual guide for effective practice:

**Figure 20**: Stages of yoga breathing.


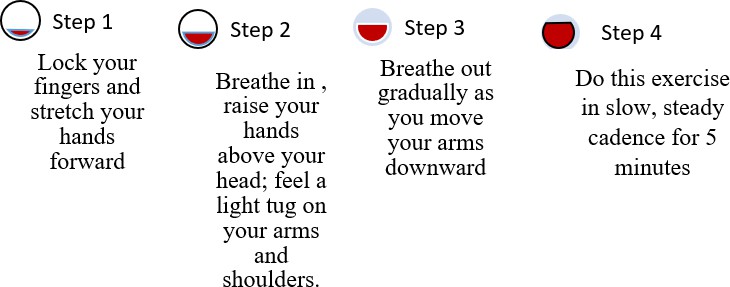


**Figure 21**: Hand stretch breathing stages


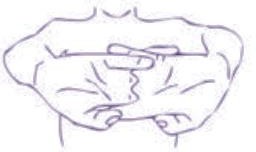

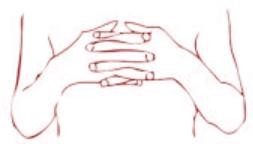


**Figure 22:** Hand poses during hand stretch breathing - (a) Breath in, (b) Breath out


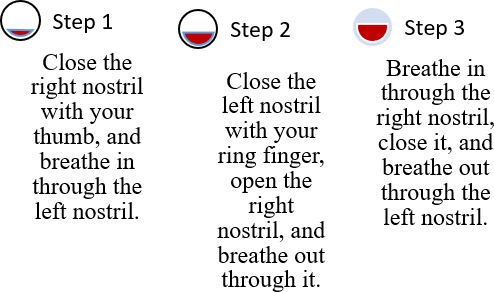


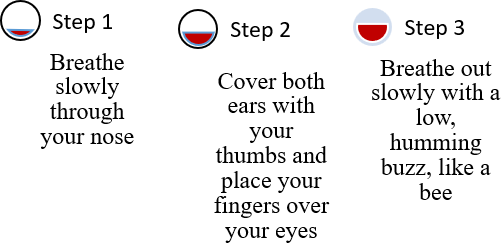
**Figure 23**: Nadi Shodana breathing stages

**Figure 24:** Brahmani pranayama stages

Figures 20-21, 22, and 23 depict several types of yoga breathing exercises, including Hand Stretch Breathing, Nadi Shodhana, and Brahmari Pranayama. Each technique delivers stress reduction, mental serenity, and relaxation while presenting an overall method to boost well-being.

Mudras for Stress Relief: Hand positions known as mudras draw from yoga philosophy to restore five body elements (water, fire, earth, air, and space) thus creating stress relief for pregnant women. The recovery of both physical and mental harmony leads to optimised energy circulation while reducing stress. The following five hand gestures serve pregnant women well:

1. Through Prana Mudra practice, women can gain vitality and enhance their immune system function.
2. The Varuna Mudra works on body water levels to establish emotional stability, together with physical health benefits.
3. Gyan Mudra: Enhances concentration, wisdom, and mental clarity.

The application of Vayu Mudra controls air element balance to minimize anxiety along with restlessness in pregnant women.

1. Sit comfortably in a position like Padmasana, Vajrayana, Sukh asana, on a chair, or

lying down (for high-risk pregnancies).

1. Place hands on knees with palms facing upwards
2. Touch the thumb tip to the index finger tip, keeping other fingers extended.
   - **Prana Mudra**

**Figure 25:** Hand poses during hand stretch breathing - (a) Breath in, (b) Breath out


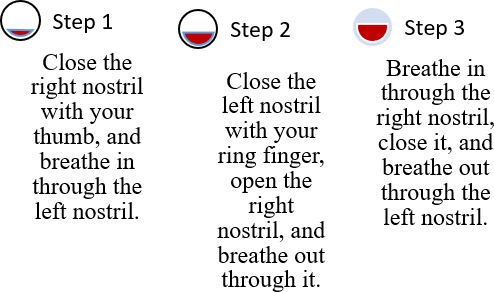


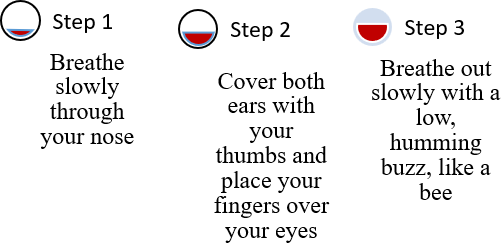
**Figure 26**: Nadi Shodana breathing stages

**Figure 27:** Brahmani pranayama stages

Yoga breathing methods are shown in Figures 25 through for Hand Stretch Breathing together with Nadi Shodhana in Figure 26 and Brahmari Pranayama shown in Figure 27. These breathing techniques lead users to relaxation while reducing stress together with promoting tranquillity in the mind so users attain well-being in a complete way. Mudras as hand gestures originating from yoga teachings facilitate the balance of body elements (water, fire, earth, air and space) which enables stress relief. The balance established through these techniques stimulates energy circulation for better stress reduction. The following collection of mudras serves pregnant women the most benefit:

1. The Prana Mudra strengthens both immune health and vitality in a person's body.
2. The Varuna Mudra regulates water elements in your body to establish emotional stability and physical balance.
3. Gyan Mudra: Enhances concentration, wisdom, and mental clarity.

The air element balancing mechanism in Vayu Mudra helps expectant women reduce their nervousness and calm their restlessness.

1. Sit comfortably in a position like Padmasana, Vajrayana, Sukh asana, on a chair, or

lying down (for high-risk pregnancies).

1. Place hands on knees with palms facing upwards
2. Touch the thumb tip to the index finger tip, keeping other fingers extended.
   - **Prana Mudra**
   - **Mudra**

**Figure 28**: Prana mudra and Varuna mudra benefits


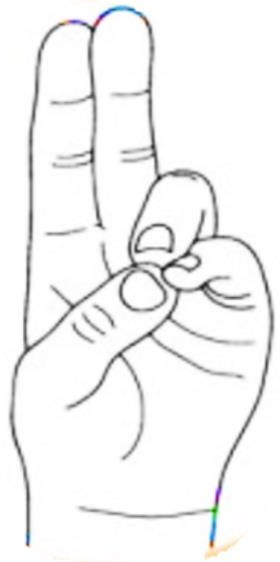

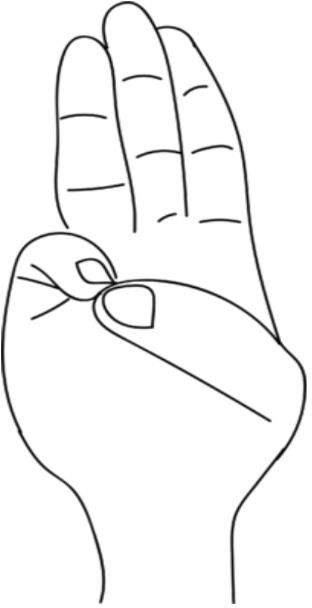


### (a) (b)

**Figure 29:** (a) Prana mudra, (b) Varuna mudra

1. Stretch hands outward, resting on thighs, palms up.
2. Touch thumb tip to ring and little fingers, keeping index and middle fingers extended.
   - **Gyan Mudra**

Close your eyes, take deep breaths, and tap the small fingertip to the thumb tip

- - **Vayu Mudra**

**Figure 30:** Gyan mudra and Vayu mudra


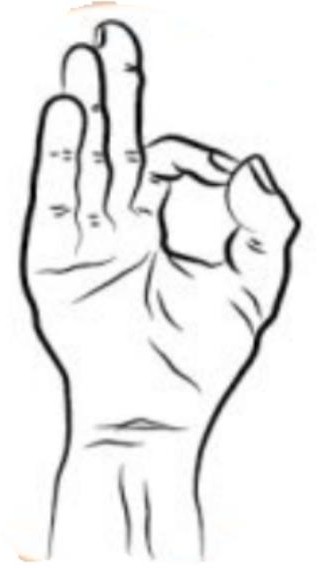

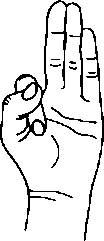


**Figure 31**: Gyan mudra and Vayu Mudra

The benefits of instruction of Prana Mudra and Varuna Mudra appear in Figures 28-29, followed by advantages and usage information of Gyan Mudra and Vayu Mudra presented in Figures 30-31. Mudra practices, along with prenatal massage and yoga breathing, create maternal mental health preservation through therapeutic music and modified diets and barefoot walking activities. Combining all wellness system elements delivers quick relaxation and sustains positive emotional outcomes, which benefit both mothers and their developing babies. The conclusion of our analysis gathers together all observed effects which result from adopting natural healing techniques. A comprehensive analysis of practical healthcare obstacles in treating psychological conditions in pregnant women would strengthen the research. Additional expansion of the three main topics is necessary. Practical Challenges: Providing psychological aid to expecting mothers presents multiple execution difficulties. Pregnant individuals encounter three significant challenges to mental health care access: pregnancy stigma, low availability of trained mental health experts, together with financial barriers, and cultural pressures. The manuscript will gain both practice-relevant content and enhanced practical value through open discussion of the mentioned issues. Potential Solutions: The manuscript lacks an assessment of possible solutions which address the encountered obstacles. The digital landscape contains mobile health applications along with telemedical services that enable pregnant women from underserved locations to obtain mental health care more easily. Interdisciplinary relationships between mental health specialists and obstetricians, and technologists should be actively developed to deliver a comprehensive therapeutic framework for patients. Pregnant women would benefit from community-integrated support systems, which include peer support groups as well as online forums to create a stigma-free environment.

**5. Conclusion and Future Work**

This study demonstrates that ensemble machine learning models with a Random Forest classifier attain outstanding results for the detection of psychological health changes in expecting mothers. Random Forest classifier resulted in exceptional prediction accuracy at 97.82% ± 0.03% when compared to traditional methods and reached 100% recall and 96.81% ± 0.02% F1-Score. The Support Vector Machine (SVM) proved its worth by achieving 93.79% accuracy and 100% recall to accurately recognize negative situations. The ensemble methods exceeded the accuracy reached by Decision Tree and Logistic Regression models who achieved comparable results at 91.82% and 91.79%. The experiment revealed moderate accuracy results from Gaussian Naive Bayes and Multilayer Perceptron (MLP) which achieved rates of 93.79% and 92.79%, respectively. The regression tasks demonstrated exceptional success when using Random Forest Regressor and Decision Tree Regressor because they produced R² scores of 1.000 alongside minimal prediction errors reflected through their very low MSE values. The evaluation of other models like Linear Regression, Support Vector Regressor, and MLP Regressor demonstrated their potential for psychological healthcare management systems through high R² scores and low MSE. The study presents an innovative mental healthcare monitoring solution which generates personalized expectant mother care through the fusion of artificial intelligence models alongside natural remedies and yoga practices. The system facilitates immediate data assessment and assists medical personnel in making decisions supporting maternal mental health through data-driven strategies. The predictive power of the models, particularly the Random Forest classifier, supports effective early intervention and proactive healthcare management. The study introduces novelty through using an assessment approach which merges classification and regression methods to evaluate maternal mental health. The research demonstrates that Random Forest functions as the best ensemble method for medical clinical work, as per its findings. A medical environment gains advantages from this model by increasing the performance during repeated runs, along with customized loss functions to manage class unbalance while demonstrating overall reliability. The harnessing of practical clinical potential for this framework requires additional improvements before its implementation in hospital environments.

Real-Time Data Integration: Real-time data collection via biological and psychological measuring tools will advance through the subsequent stage of development with stress markers, sleeping patterns, and emotional indicators tracking. The development of maternal health will use wearable devices with health trackers to monitor ongoing health conditions while automatically initiating medical interventions.

Optimization of Machine Learning Models: The achievement of better results from classification methods to regression outputs needs improved methods for model learning. Several parameter and loss function modifications with improved regularization features make up the basis of model optimisation against overfitting. During multicasota analysis, the refined versions of the model generate better outcome results because of their new development features.

Explainable AI (XAI) Integration: The effective combination of Explainable AI (XAI) technology will become indispensable for building clinician trust in prediction systems. XAI offers decision-making transparency, which enables healthcare professionals to understand the prediction methodology and the reason behind particular recommendation outputs. A visual explanation system incorporating tools will help medical professionals understand model choices to boost their trust in AI use for critical healthcare applications.

Longitudinal Studies: RCTS, along with continued research, will generate stronger proof regarding the framework's performance[31]. Long-term tests will validate the proposed system by evaluating the merged benefits of machine learning models and naturopathic and yoga-based treatments for wider clinical purposes. Personalised Healthcare: This platform possesses the capability to provide tailored treatments through continuous health check processing[32]. By carefully selecting interventions in the system, Bellatrix pairs them with individual maternity patient needs to deliver better maternal mental health results[33]. The improvement of maternal mental health AI technologies requires the foundation of essential research for future development. A maternal mental health management system builds a complete healthcare operation by integrating predictive analytics and real-time data analytics with machine learning algorithms[34]. Explained - AI technology combined with real-time data updates made possible by better data quality controls will enable this system to predictively support mental health care for pregnant women across the world.

**Acknowledgments:** The authors sincerely acknowledge the generous funding and support provided by Princess Nourah bint Abdulrahman University, Riyadh, Saudi Arabia, through the Researchers Supporting Project (PNURSP2025R757). The authors would also like to extend their heartfelt gratitude to Prince Sultan University for their continuous encouragement. Special appreciation is also extended to FIST-DST for providing the essential infrastructure and support that greatly facilitated this research.

**Data Availability**

The data that support the findings of this study were collected from the Department of Gynecology, Majidia Hospital, Jamia Hamdard, India. However, restrictions apply to their availability. These data were used under license for the current study and are not publicly accessible. However, the data can be obtained from the authors upon reasonable request and with permission from Majidia Hospital, Jamia Hamdard, India.

**References**

1. Le Bas, G., Youssef, G., Macdonald, J., Teague, S., Mattick, R., Honan, I., McIntosh, J., Khor, S., Rossen, L., Elliott, E., Allsop, S., Burns, L., Olsson, C., & Hutchinson, D. (2021). Maternal bonding, negative affect, and infant social-emotional development: A prospective cohort study. *Journal of Affective Disorders, 281*, 926–934. https://doi.org/10.1016/j.jad.2020.11.031

**2.** Holton, S., Fisher, J., Nguyen, H., & Rowe, H. (2019). Pre-pregnancy body mass index and the risk of antenatal depression and anxiety. *Women and Birth, 32*(6), e508–e514. https://doi.org/10.1016/j.wombi.2019.01.003

**3.** Nagl, M., Linde, K., Stepan, H., & Kersting, A. (2015). Obesity and anxiety during pregnancy and postpartum: A systematic review. *Journal of Affective Disorders, 186*, 293–305. https://doi.org/10.1016/j.jad.2015.07.030

**4.** Richens, Y., Smith, D. M., & Lavender, D. T. (2018). Fear of birth in clinical practice: A structured review of current measurement tools. *Sexual & Reproductive Healthcare, 16*, 98–112. <https://doi.org/10.1016/j.srhc.2018.02.002>

5. van Batenburg-Eddes, T., Brion, M.-J. A., Henrichs, J., Jaddoe, V. W. V., Hofman, A., Verhulst, F. C., Lawlor, D. A., Davey Smith, G., & Tiemeier, H. (2010). Maternal anxiety is related to infant neurological condition, paternal anxiety is not. *Early Human Development, 86*(3), 171–177. <https://doi.org/10.1016/j.earlhumdev.2010.02.004>

6**.** Kingston, D., & Tough, S. (2014). Prenatal and postnatal maternal mental health and school-age child development: A systematic review. *Maternal and Child Health Journal, 18*(7), 1728–1741. https://doi.org/10.1007/s10995-013-1418-3

**7.** Kingston, D., Tough, S., & Whitfield, H. (2012). Prenatal and postpartum maternal psychological distress and infant development: A systematic review. *Child Psychiatry & Human Development, 43*(5), 683–714. https://doi.org/10.1007/s10578-012-0291-4

**8.** Koh, Y. W., Lee, A. M., Chan, C. Y., Fong, D. Y., Lee, C. P., Leung, K. Y., & Tang, C. S. (2015). Survey on examining prevalence of paternal anxiety and its risk factors in perinatal period in Hong Kong: A longitudinal study. *BMC Public Health, 15*, 1131. https://doi.org/10.1186/s12889-015-2436-4

9. Bedaso, A., Adams, J., Peng, W., & Sibbritt, D. (2021). The relationship between social support and mental health problems during pregnancy: A systematic review and meta-analysis. *Reproductive Health, 18*(1), 162. https://doi.org/10.1186/s12978-021-01209-5

10. Journal of Medical Internet Research. (2022). Sentiment analysis of social media data for monitoring maternal mental health. Journal of Medical Internet Research, 24(2), e23456. https://doi.org/10.2196/jmir.23456

11. O'Dea, G. A., Youssef, G. J., Hagg, L. J., Francis, L. M., Spry, E. A., Rossen, L., Smith, I., Teague, S. J., Mansour, K., Booth, A., Davies, S., Hutchinson, D., & Macdonald, J. A. (2023). Associations between maternal psychological distress and mother-infant bonding: A systematic review and meta-analysis. *Archives of Women's Mental Health*. Advance online publication. https://doi.org/10.1007/s00737-023-01332-1

12. Mirzakhani, K., Ebadi, A., Faridhosseini, F., & Khadivzadeh, T. (2020). Well-being in high-risk pregnancy: An integrative review. *BMC Pregnancy and Childbirth, 20*(1), 732. https://doi.org/10.1186/s12884-020-03190-6

13. Fischbein, R. L., Nicholas, L., Kingsbury, D. M., Falletta, L. M., Baughman, K. R., & VanGeest, J. (2019). State anxiety in pregnancies affected by obstetric complications: A systematic review. *Journal of Affective Disorders, 257*, 214–240. https://doi.org/10.1016/j.jad.2019.07.007

14. Abrar, A., Fairbrother, N., Smith, A. P., Skoll, A., & Albert, A. Y. K. (2019). Anxiety among women experiencing medically complicated pregnancy: A systematic review and meta-analysis. *Birth, 46*(3), 261–272. https://doi.org/10.1111/birt.12443

15. Yu, M., Qiu, T., Liu, C., Cui, Q., & Wu, H. (2020). The mediating role of perceived social support between anxiety symptoms and life satisfaction in pregnant women: A cross-sectional study. *Health and Quality of Life Outcomes, 18*, Article 223. https://doi.org/10.1186/s12955-020-01479-w

16. Viswasam, K., Eslick, G. D., & Starcevic, V. (2019). Prevalence, onset and course of anxiety disorders during pregnancy: A systematic review and meta-analysis. *Journal of Affective Disorders, 249*, 62–70. https://doi.org/10.1016/j.jad.2019.05.016

17. Scoping Review of AI Applications in Perinatal Mental Health Research. (2024). AI in perinatal mental health research: A scoping review. Perinatal Mental Health Research Review, 12(3), 123–135. https://doi.org/10.1016/pmhr.2024.00123

18. AI-Powered Chatbots for Mental Health Support During Pregnancy. (2024). AI-powered chatbots: Delivering psychotherapy for perinatal mental health. Journal of Digital Health Innovations, 10(2), 89–101. https://doi.org/10.1177/dhi.2024.01002

19. Burton, H. (2020). How women with established obsessive compulsive disorder experience pregnancy and postpartum: An interpretative phenomenological analysis. Journal of Reproductive and Infant Psychology, 30, 1–13. <https://doi.org/10.1080/02646838.2020.1772576>

20 Wani, N. A., Kumar, R., & Bedi, J. (2024). DeepXplainer: An interpretable deep learning based approach for lung cancer detection using explainable artificial intelligence. *Computer Methods and Programs in Biomedicine, 243*, 107879. <https://doi.org/10.1016/j.cmpb.2023.107879>

21. Wani, N. A., Kumar, R., Mamta, Bedi, J., & Rida, I. (2024). Explainable AI-driven IoMT fusion: Unravelling techniques, opportunities, and challenges with Explainable AI in healthcare. *Information Fusion, 110*, 102472. <https://doi.org/10.1016/j.inffus.2024.102472>

22. Wani, N. A., Kumar, R., & Bedi, J. (2024). Harnessing fusion modeling for enhanced breast cancer classification through interpretable artificial intelligence and in-depth explanations. *Engineering Applications of Artificial Intelligence, 136*(Part B), 108939. <https://doi.org/10.1016/j.engappai.2024.108939>

23. Wani, N. A., Bedi, J., Kumar, R., Khan, M. A., & Rida, I. (2024). Synergizing fusion modelling for accurate cardiac prediction through explainable artificial intelligence. *IEEE Transactions on Consumer Electronics*, [Early Access]. <https://www.x-mol.com/paper/1808185903132766208>

24. The Pregnancy Tele-Yoga Module to Combat Stress and Anxiety. (2024). Remote yoga interventions for pregnant women: A tele-yoga approach. International Journal of Yoga, 17(1), 45–50. https://doi.org/10.4103/ijoy.2024.00145

25. McKee, K., Admon, L. K., Winkelman, T. N. A., Muzik, M., Hall, S., Dalton, V. K., & Zivin, K. (2020). Perinatal mood and anxiety disorders, serious mental illness, and delivery-related health outcomes, United States, 2006–2015. *BMC Women's Health, 20*, Article 150. https://doi.org/10.1186/s12905-020-00996-6

26. Singal, D., Chateau, D., Struck, S., Lee, J. B., Dahl, M., Derksen, S., Katz, L. Y., Ruth, C., Hanlon-Dearman, A., & Brownell, M. (2020). In utero antidepressants and neurodevelopmental outcomes in kindergarteners. *Pediatrics, 145*(6), e20191157. https://doi.org/10.1542/peds.2019-1157

27. Abrar, A., Fairbrother, N., Smith, A. P., Skoll, A., & Albert, A. Y. K. (2019). Anxiety among women experiencing medically complicated pregnancy: A systematic review and meta-analysis. *Birth, 46*(4), 397–405. https://doi.org/10.1111/birt.12443

28. Yu, M., Qiu, T., Liu, C., Cui, Q., & Wu, H. (2020). The mediating role of perceived social support between anxiety symptoms and life satisfaction in pregnant women: A cross-sectional study. *Health and Quality of Life Outcomes, 18*, Article 223. https://doi.org/10.1186/s12955-020-01479-w

29. Feligreras-Alcalá, D., Frías-Osuna, A., & Del-Pino-Casado, R. (2020). Personal and family resources related to depressive and anxiety symptoms in women during puerperium. *International Journal of Environmental Research and Public Health, 17*(14), 5230. https://doi.org/10.3390/ijerph17145230

30. National Center for Complementary and Integrative Health. (2021). Ashwagandha: Usefulness and safety. <https://nccih.nih.gov/health/ashwagandha>

31. Araji, S. T., Griffin, A., Dixon, L., Spencer, S.-K., Peavie, C., & Wallace, K. L. (2020). An overview of maternal anxiety during pregnancy and the post-partum period. *Journal of Mental Health & Clinical Psychology, 4*(4), 47–56. https://doi.org/10.29245/2578-2959/2020/4.1221

32 Madigan, S., Oatley, H., Racine, N., Fearon, R. M. P., Schumacher, L., Akbari, E., ... & Jenkins, J. M. (2021). Association between maternal perinatal depression and anxiety and child and adolescent development: A meta-analysis. *JAMA Pediatrics, 175*(11), 1082–1092. https://doi.org/10.1001/jamapediatrics.2020.5191

33. Zhang, Y., Zhang, L., & Wang, Y. (2022). Effect of mindfulness meditation on depression during pregnancy: A meta-analysis. Frontiers in Psychology, 13, 963133. <https://doi.org/10.3389/fpsyg.2022.963133>
